# Modular cytokine receptor-targeting chimeras for targeted degradation of cell surface and extracellular proteins

**DOI:** 10.1038/s41587-022-01456-2

**Published:** 2022-09-22

**Authors:** Katarina Pance, Josef A. Gramespacher, James R. Byrnes, Fernando Salangsang, Juan-Antonio C. Serrano, Adam D. Cotton, Veronica Steri, James A. Wells

**Affiliations:** 1 Department of Pharmaceutical Chemistry, University of California San Francisco, San Francisco, CA USA; 2 Helen Diller Family Comprehensive Cancer Center, University of California San Francisco, San Francisco, CA USA; 3 Preclinical Therapeutics Core, University of California San Francisco, San Francisco, CA USA; 4 Department of Cellular and Molecular Pharmacology, University of California San Francisco, San Francisco, CA USA; 5 Present Address: EpiBiologics, Inc., San Carlos, CA USA

**Keywords:** Antibody therapy, Protein design, Chemokines

## Abstract

Targeted degradation of cell surface and extracellular proteins via lysosomal delivery is an important means to modulate extracellular biology. However, these approaches have limitations due to lack of modularity, ease of development, restricted tissue targeting and applicability to both cell surface and extracellular proteins. We describe a lysosomal degradation strategy, termed cytokine receptor-targeting chimeras (KineTACs), that addresses these limitations. KineTACs are fully genetically encoded bispecific antibodies consisting of a cytokine arm, which binds its cognate cytokine receptor, and a target-binding arm for the protein of interest. We show that KineTACs containing the cytokine CXCL12 can use the decoy recycling receptor, CXCR7, to target a variety of target proteins to the lysosome for degradation. Additional KineTACs were designed to harness other CXCR7-targeting cytokines, CXCL11 and vMIPII, and the interleukin-2 (IL-2) receptor-targeting cytokine IL-2. Thus, KineTACs represent a general, modular, selective and simple genetically encoded strategy for inducing lysosomal delivery of extracellular and cell surface targets with broad or tissue-specific distribution.

## Abstract (web summary)

KineTACs are modular bispecific antibodies for degradation of extracellular and cell-surface proteins.

## Main

Targeted degradation of cell surface and extracellular proteins through the process of lysosomal delivery is an important recent approach to modulate extracellular biology^1–4^. Targeted protein degradation has emerged over the last two decades as a promising therapeutic strategy with advantages over conventional inhibition, which relies on occupancy-driven pharmacology^5^. Unlike inhibitors, degraders can enable catalytic and durable knockdown of protein levels. Degraders also offer advantages in targeting proteins with resistance mutations^6^ and difficult to drug proteins^7,8^, along with inhibiting both catalytic and scaffolding functions^9,10^. Most degrader technologies, such as proteolysis-targeting chimeras (PROTACs)^11^ and immunomodulatory imide drugs, also known as molecular glues^12^, co-opt the ubiquitin proteasome system to degrade traditionally challenging proteins. Intracellular small-molecule degraders have demonstrated success in targeting over 60 proteins, and many are currently being tested in clinical trials^13^. However, due to their intracellular mechanism of action, these approaches are limited to targeting proteins with ligandable intracellular domains.

Given that most drug targets are located at the cell surface or secreted, there is emerging interest for developing strategies to degrade extracellular proteins. Serendipitously, some antibodies can induce target receptor internalization and subsequent degradation due to receptor clustering or through binding Fc receptors. For example, MEDI5752, a programmed cell death protein 1 (PD-1) × CTLA4 bispecific antibody, causes degradation of PD-1, and avelumab, a programmed death ligand 1 (PD-L1) monoclonal antibody, causes rapid internalization of PD-L1 due to Fc-γ receptor binding^14,15^. A more deliberate approach, termed sweeping antibodies^16,17^, uses pH-dependent antibody binders that co-opt the neonatal Fc receptor for internalization, followed by pH-dependent release and delivery of target proteins to the lysosome. This strategy requires engineering each target binder for pH dependence, which is a time-consuming process and limits the modularity of this approach. Another approach, lysosome-targeting chimeras (LYTACs), uses IgG–glycan bioconjugates to co-opt lysosome shuttling receptors^2,18^. LYTAC production requires chemical synthesis and in vitro bioconjugation of large glycans at multiple sites for effective target clearance. A third extracellular degradation platform, called antibody-based PROTACs, uses bispecific IgGs to hijack cell surface E3 ligases^1^. Due to the dependence on intracellular ubiquitin transfer, antibody-based PROTACs are limited to targeting cell surface proteins, leaving the secreted proteome out of reach. Thus, there remains a critical need to develop additional extracellular degradation technologies that are fully genetically encoded.

**Fig. 1:**
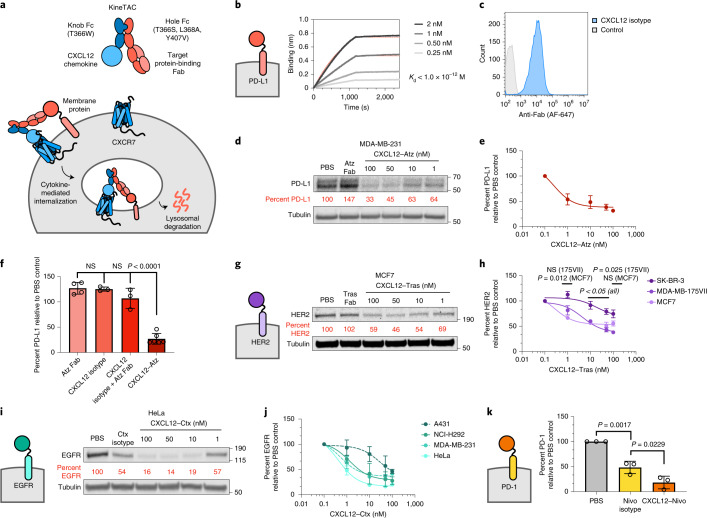
KineTAC platform for targeted protein degradation of therapeutically relevant cell surface proteins. **a**, Schematic of the KineTAC concept for targeting cell surface proteins for lysosomal degradation via CXCL12-mediated endocytosis. **b**, Multipoint BLI measurement of CXCL12–Atz shows high affinity to PD-L1 Fc fusion. **c**, Flow cytometry showing CXCL12 isotype binding on MDA-MB-231 cells endogenously expressing CXCR7. **d**–**e**, Dose escalation experiment showing PD-L1 degradation in MDA-MB-231 cells after 24 h of treatment with CXCL12–Atz; *n* = 3 biologically independent experiments. **f**, PD-L1 levels were significantly reduced after a 24-h treatment of MDA-MB-231 cells with 100 nM CXCL12–Atz (*P* < 0.0001) compared to Atz Fab alone but not with CXCL12 isotype (*P* = 0.1946) or a combination of Atz Fab and CXCL12 isotype (*P* = 0.8262); *n* = 4, 3, 3 or 6 biologically independent experiments, respectively; NS, not significant. **g**, Dose escalation experiment showing HER2 degradation in MCF7 cells after 24-h treatment with CXCL12–Tras or 100 nM Tras Fab. **h**, Summary of HER2 degradation in various HER2-expressing cell lines following 24-h treatment with CXCL12–Tras. Data show significantly greater HER2 degradation in MDA-MB-175VII (*P* = 0.0248) than for SK-BR-3 cells, but not for MCF7 (*P* = 0.1003); *n* = 2 biologically independent experiments. **i**, Dose escalation showing EGFR degradation in HeLa cells after 24-h treatment with CXCL12–Ctx or 100 nM Ctx isotype. **j**, Summary of EGFR degradation in various EGFR-expressing cell lines following 24-h treatment with CXCL12–Ctx; *n* = 2 biologically independent experiments for A431, NCI-H292 and MDA-MB-231 cells, and *n* = 3 biologically independent experiments for HeLa cells. **k**, Summary of flow cytometry data demonstrating significant degradation of cell surface PD-1 on activated primary human CD8^+^ T cells after treatment with 100 nM CXCL12–Nivo compared to treatment with Nivo isotype (*P* = 0.0229). Percent PD-1 was determined by median fluorescence intensity (MFI) of the PE fluorescence channel of live cells; *n* = 10,000 live cells analyzed over three biologically independent experiments. Densitometry was used to calculate protein levels, and data were normalized to PBS control. Data are represented as mean values, and error bars represent the standard deviation of biological replicates. *P* values were determined by one-way analysis of variance (ANOVA) with Sidak’s multiple comparisons test. Source data

Here, we have developed a targeted degradation platform, termed cytokine receptor-targeting chimeras (KineTACs). KineTACs are fully recombinant bispecific antibodies built on human scaffolds that use endogenous cytokine-mediated internalization of cognate receptors to enable broad and efficient lysosomal delivery of both cell surface and extracellular proteins. To exemplify the utility of this platform, we focused first on the chemokine CXCL12, which binds the decoy receptor CXCR7 and is constitutively internalized due to β-arrestin recruitment without downstream signaling^19^. CXCL12 also binds the signaling receptor CXCR4, which is internalized following G-protein and β-arrestin recruitment and shuttles to the lysosome. However, CXCL12 binds with tenfold higher affinity to CXCR7 than to CXCR4 (dissociation constant (*K*_d_) values of 0.014 and 2.1 nM, respectively)^20^, making CXCR7 the dominant recycling receptor. Here, we show that KineTACs bearing CXCL12 can efficiently use CXCR7 internalization for lysosomal degradation applications and are generalizable against eight therapeutically relevant extracellular membrane and soluble proteins (Fig. 1a).

## Results

Overexpression of PD-L1 on cancer cells leads to engagement of T cell checkpoint protein PD-1 and suppression of cytotoxic T cell activity^21^. To demonstrate proof of concept, we generated a KineTAC targeting PD-L1 using generic knob-in-hole bispecific antibody–cytokine fusions^22^, in which the human CXCL12 chemokine was N-terminally fused to the knob Fc domain, and the second arm contained the antigen-binding fragment (Fab) antibody sequence for atezolizumab (Atz; Tecentriq), a Food and Drug Administration (FDA)-approved inhibitor of PD-L1, fused to the hole Fc domain (Fig. 1a). The T336W mutation and T366S, L368A and Y407V mutations were introduced into the IgG1 Fc domain to generate the knob and hole Fc domains, respectively. For mechanistic simplification, the N297G mutation was also introduced to remove effector functions.

**Extended Data Fig. 1:**
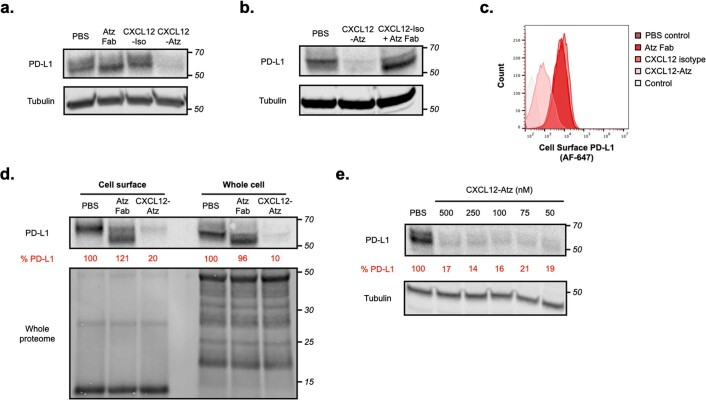
KineTACs target cell surface protein PD-L1 for degradation. **a**, Representative western blot showing PD-L1 levels after 24 hr treatment of MDA-MB-231 cells with 100 nM of atezolizumab control, CXCL12 isotype, or CXCL12-Atz. **b**, Representative western blot showing PD-L1 levels after 24 hr treatment of MDA-MB-231 cells with 100 nM CXCL12-Atz or 100 nM CXCL12 isotype + 100 nM atezolizumab Fab. **c**, Flow cytometry showing degradation of surface PD-L1 on MDA-MB-231 cells after 24 hr treatment with 100 nM CXCL12-Atz, but not after addition of controls. **d**, Levels of cell surface and whole cell PD-L1 after 24 hr treatment of MDA-MB-231 cells with 100 nM CXCL12-Atz or atezolizumab Fab shows marginal differences between cell surface and whole cell PD-L1 levels. **e**, Representative western blot showing PD-L1 levels after 24 hr treatment of MDA-MB-231 cells with high concentrations (50–500 nM) of CXCL12-Atz shows that no ‘hook effect’ is observed in this concentration range. Data are representative of at least three independent biological replicates. Densitometry was used to calculate protein levels and normalized to PBS control. Source data

One advantage of KineTACs is that production of cytokine-bearing bispecifics is not complicated by light chain–heavy chain mispairing problems, which are common to bispecific IgGs containing Fabs on both arms. Moreover, the KineTAC platform only requires engineering a binder to the target protein, as the natural cytokine serves to recruit the degrading receptor. Given these advantages, we were able to express both arms in a single mammalian construct to allow complete assembly of the KineTAC^23,24^. A His tag was introduced on the knob arm to allow purification of the knob–hole bispecific from contaminants of unwanted hole–hole homodimers that may form. Next, we confirmed that the PD-L1-targeting KineTAC (termed CXCL12–Atz) retains binding to PD-L1 by using biolayer interferometry (BLI; Fig. 1b). Furthermore, an isotype control of the CXCL12 KineTAC, which incorporates a control Fab arm targeting the severe acute respiratory syndrome coronavirus 2 spike protein^25^, retained binding to endogenous CXCR7 expressed on MDA-MB-231 cells, a breast cancer cell line (Fig. 1c). These data suggest that both anti-PD-L1 and CXCL12 arms of the KineTAC are functional in the bispecific context. To determine the potency of CXCL12–Atz to degrade PD-L1, MDA-MB-231 cells were treated with varying concentrations of the KineTAC. After 24 h of treatment, levels of PD-L1 were quantified by western blotting. Both glycosylated forms of PD-L1 were substantially degraded, with a maximal percent degradation (*D*_max_) of roughly 70% (Fig. 1d,e). The single-arm Atz Fab or the CXCL12 isotype did not induce the degradation of PD-L1 either alone or in combination, indicating that PD-L1 degradation is dependent on the bispecific KineTAC scaffold (Fig. 1f and Extended Data Fig. 1a,b). Finally, flow cytometry and western blotting of whole-cell lysates verified that PD-L1 is depleted and degraded from the cell surface (Extended Data Fig. 1c,d). Bivalent molecules can induce a ‘hook effect’ at very high concentrations where both target proteins are engaged singly with the bivalent molecule, which could reduce the degradation efficiency^26^. However, we did not observe reduction of degradation after treatment with high concentrations of CXCL12–Atz (up to 500 nM), which indicates potent degradation over a large dose range (Extended Data Fig. 1e).

**Extended Data Fig. 2:**
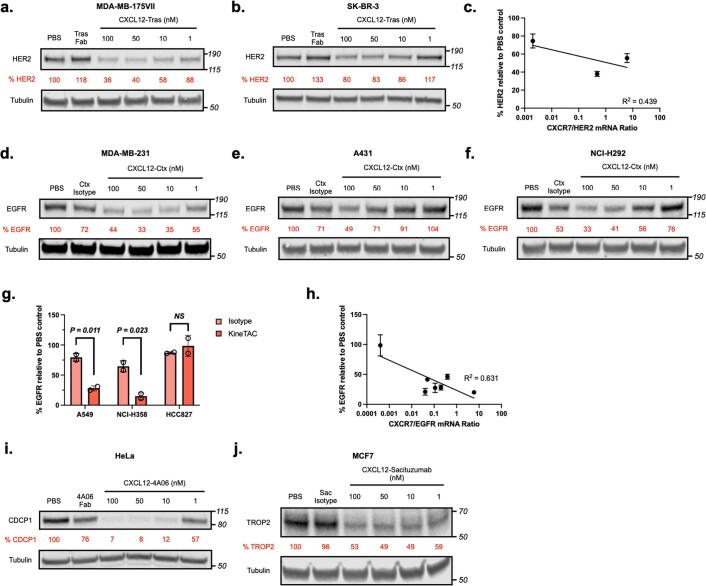
KineTACs mediate degradation of additional therapeutically relevant cell surface proteins. Dose escalation showing HER2 degradation in **a**, MDA-MB-175VII and **b**, SK-BR-3 cells following treatment with CXCL12-Tras or 100 nM trastuzumab Fab. **c**, Trend of HER2 levels to CXCR7/HER2 transcript level ratio as calculated by densitometry after treatment with 100 nM CXCL12-Tras for 24 hrs in MCF7, MDA-MB-175VII, and SK-BR-3 cells. *N* = 2 biologically independent experiments. Dose escalation showing EGFR degradation in **d**, MDA-MB-231, **e**, A431, and **f**, NCI-H292 cells following treatment with CXCL12-Ctx or 100 nM Cetuximab isotype. **g**, EGFR levels after treatment with 100 nM CXCL12-Ctx were significantly reduced compared to Ctx isotype control for 24 hrs in non-small cell lung cancer cell lines A549 (*P* = 0.011) and NCI-H358 (*P* = 0.023) but were unchanged in HCC827 (*P* = 0.4559) where EGFR is substantially overexpressed. *N* = 2 biologically independent experiments. **h**, Trend of EGFR levels to CXCR7/EGFR transcript level ratio as calculated by densitometry after treatment with 100 nM CXCL12-Ctx for 24 hrs in HeLa, A431, NCI-H292, MDA-MB-231, A549, NCI-H358, and HCC827 cells. *N* = 3 biologically independent experiments for HeLa and *N* = 2 biologically independent experiments for remaining cell lines. **i**, Dose escalation showing CDCP1 degradation in HeLa cells following treatment with CXCL12-4A06 or 100 nM 4A06 Fab. **j**, Dose escalation showing TROP2 degradation in MCF7 cells following treatment with CXCL12-Sacituzumab or sacituzumab isotype. Densitometry was used to calculate protein levels and normalized to PBS control. Data are represented as mean values and error bars represent the standard deviation of biological replicates. *P*-values were determined by unpaired two-tailed *t-*tests. Source data

We next sought to determine whether the KineTAC degradation platform could be extended to other therapeutically relevant cell surface proteins. First, we targeted human epidermal growth factor receptor 2 (HER2), which is frequently upregulated in cancer and linked to breast cancer invasiveness and tumor progression^27^. A KineTAC targeting HER2 was produced by incorporating the Fab arm from trastuzumab (Tras; Herceptin), an FDA-approved HER2 inhibitor, into the KineTAC scaffold (termed CXCL12–Tras). Various breast cancer cell lines expressing HER2 were incubated for 24 h with CXCL12–Tras. Substantial degradation of HER2 was observed in MCF7 and MDA-MB-175VII cells, with *D*_max_ values of 51 and 62%, respectively (Fig. 1g,h and Extended Data Fig. 2a). A lower percent degradation was observed in SK-BR-3 cells, likely because HER2 is substantially overexpressed relative to CXCR7 in this cell line^28^ (Fig. 1h and Extended Data Fig. 2b). Generally, we find that the maximal percent degradation mediated by KineTACs across different cell lines was inversely related to the expression of CXCR7 relative to HER2 (*R*^2^ = 0.439; Extended Data Fig. 2c).

Next, we targeted epidermal growth factor receptor (EGFR) for degradation. EGFR is implicated as a driver of cancer progression, and EGFR inhibitors are approved for use in non-small cell lung, colorectal and gastric cancers^29–31^. We developed a KineTAC targeting EGFR by incorporating cetuximab (Ctx; Erbitux), an FDA-approved EGFR inhibitor, into the KineTAC scaffold (termed CXCL12–Ctx). Following 24 h of treatment with CXCL12–Ctx, EGFR levels were dramatically reduced in HeLa cells, with a *D*_max_ of 84% (Fig. 1i,j). This result was recapitulated in various breast and lung cancer cell lines, including MDA-MB-231, A431, NCI-H292, A549 and NCI-H358 cells. As with HER2, the maximum percent degradation was inversely related to the expression of CXCR7 relative to EGFR (*R*^2^ = 0.631; Fig. 1j and Extended Data Fig. 2d–h).

**Extended Data Fig. 3:**
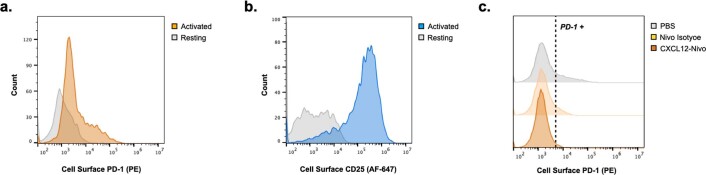
KineTACs mediate degradation of PD-1 on primary human CD8 + T cells. Representative flow cytometry showing presence of CD8 + T cell activation markers, **a**, PD-1 or **b**, CD25, following 4 day incubation with activation cocktail (IL-2, IL-15, anti-CD3, anti-CD28). **c**, Representative flow demonstrating cell surface PD-1 degradation on activated primary human CD8 + T cells following 24 hr treatment with 100 nM CXCL12-Nivo or nivolumab isotype.

We next determined if the KineTACs could be expanded to other tumor-associated membrane proteins . To this end, a KineTAC was constructed from an antibody targeting CUB domain-containing protein 1 (CDCP1)^32^, which is upregulated in many cancers at levels to over 1 million copies per cell. We observed near complete degradation of CDCP1 after 24 h of treatment in HeLa cells, with a *D*_max_ of 93% (Extended Data Fig. 2i). KineTACs also enabled the degradation of tumor-associated calcium signal transducer 2 (TROP2), the overexpression of which has been linked to tumor progression in a variety of tumors^33,34^. In MCF7 cells, we observed a *D*_max_ of 51% after treatment with a TROP2-targeting KineTAC (Extended Data Fig. 2j). We then tested whether KineTACs can degrade the checkpoint protein PD-1 in CD8^+^ T cells isolated from primary human peripheral blood mononuclear cells (PBMCs). T cells were first activated to induce upregulation of PD-1 on the cell surface along with other activation markers (Extended Data Fig. 3a,b). The activated T cells were then treated for 24 h with a PD-1-targeting KineTAC, which incorporated the antibody sequence for nivolumab (Nivo; Opdivo), an FDA-approved PD-1 inhibitor (termed CXCL12–Nivo). Following treatment with CXCL12–Nivo, the proportion of PD-1 on the surface of T cells was reduced by 82% compared to treatment with the Nivo isotype control, which is known to induce slight internalization of PD-1 (Fig. 1k and Extended Data Fig. 3c)^35^. Overall, these results on six different membrane proteins demonstrate the generality of the KineTAC platform for degrading a variety of cell surface proteins (Supplementary Table 1).

**Fig. 2:**
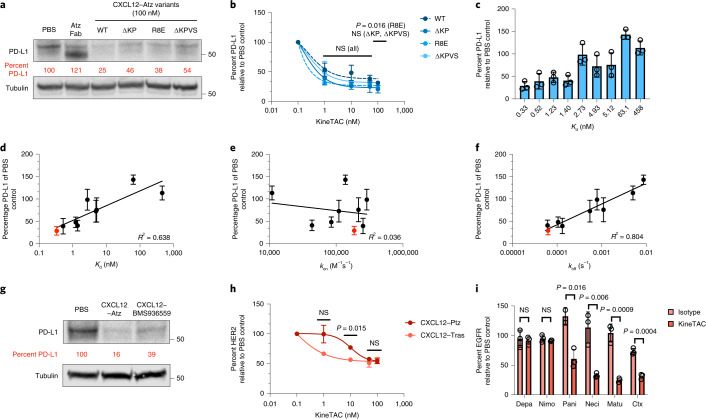
Requirements for efficient KineTAC-mediated degradation of target proteins. **a**,**b**, Treatment with 100 nM antagonistic (∆KP, ∆KPVS and R8E) CXCL12–Atz variants for 24 h in MDA-MB-231 cells shows no significant difference in PD-L1 levels compared to agonistic CXCL12^WT^–Atz at 50 nM (*P* = 0.1432, 0.1222 and 0.5016, respectively). At 100 nM, CXCL12^R8E^–Atz showed significantly greater PD-L1 degradation than CXCL12^WT^–Atz (*P* = 0.0162), while CXCL12^∆KP^–Atz and CXCL12^∆KPVS^–Atz were unchanged (*P* = 0.0609 and 0.7538, respectively); *n* = 3 biologically independent experiments for CXCL12^WT^–Atz, and *n* = 2 for CXCL12–Atz variants. **c**, PD-L1 levels in MDA-MB-231 cells after treatment with CXCL12–Atz wild type or alanine mutants (100 nM) for 24 h. **d**–**f**, Correlation of PD-L1 levels as calculated by densitometry and *K*_d_ (**d**), *k*_on_ (**e**) or *k*_off_ (**f**). Wild-type Atz is indicated in red; *n* = 3 biologically independent experiments. **g**, PD-L1 levels after treatment with 100 nM CXCL12–Atz or pH-sensitive binder CXCL12–BMS936559 for 24 h in MDA-MB-231 cells. **h**, HER2 levels after treatment with 100 nM CXCL12–Tras or CXCL12–Ptz in MCF7 cells demonstrate that different epitope binders affect the half-maximum degradation concentration (DC_50_) of HER2 degradation, while the *D*_max_ at 100 nM was unchanged (*P* = 0.7758); *n* = 2 biologically independent experiments. **i**, EGFR levels after treatment with 100 nM CXCL12–Ctx, CXCL12–Depa, CXCL12–Nimo, CXCL12–Matu, CXCL12–Neci or CXCL12–Pani in HeLa cells demonstrate that there is dependence on EGFR binding epitope for degradation efficiency. CXCL12–Ctx, CXCL12–Pani, CXCL12–Neci and CXCL12–Matu significantly degrade EGFR compared to isotype controls (*P* = 0.0004, 0.016, 0.006 and 0.009, respectively), while CXCL12–Depa and CXCL12–Nimo do not cause significant EGFR degradation (*P* = 0.7619 and 0.3573, respectively); *n* = 3 biologically independent experiments. Densitometry was used to calculate protein levels, and data were normalized to PBS control. Data are represented as mean values, and error bars represent the standard deviation of biological replicates. *P* values were determined by unpaired two-tailed *t-*tests. Linear regression analysis using GraphPad Prism was used to calculate the coefficient of determination (*R*^2^) to determine the strength of linear correlation. Source data

Next, we evaluated how properties, such as receptor signaling, binding affinity and binding epitope, of the KineTAC affected degradation. In addition to binding the recycling receptor CXCR7, CXCL12 binds the signaling receptor CXCR4, which causes downstream signaling followed by receptor internalization and degradation. It is possible that signaling through CXCR4 could be counterproductive when using KineTACs to target cancer drivers because CXCR4 overexpression and agonism are linked to tumor metastasis^36^. To test if CXCR4 signaling is critical for the KineTAC mechanism of action, we produced known antagonistic variants of CXCL12 (∆KP, ∆KPVS and R8E) that retain high binding affinity to both CXCR7 and CXCR4 but prevent CXCR4 signaling^19,37^. These were incorporated into the KineTAC scaffold with Atz. Following 24-h treatment in MDA-MB-231 cells, all three antagonistic variants retained the ability to degrade PD-L1 to a similar degree as CXCL12 wild type (CXCL12^WT^; Fig. 2a,b). These data suggest that CXCL12 signaling through CXCR4 is not critical for degradation.

**Extended Data Fig. 4:**
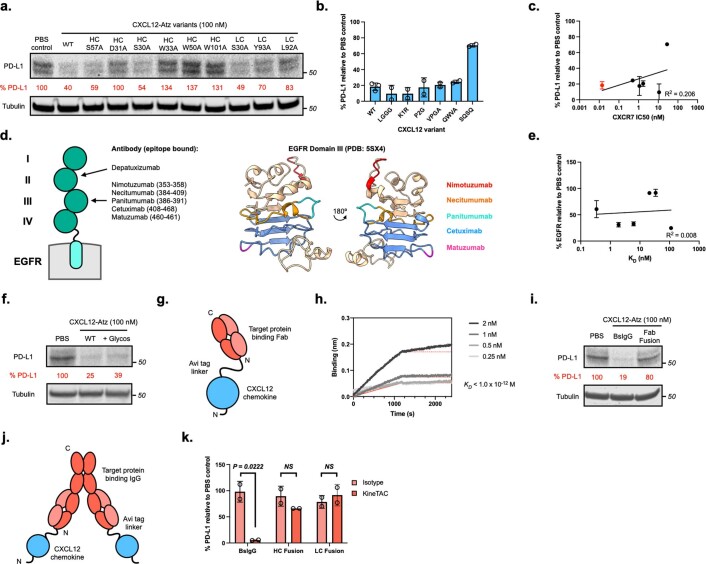
Requirements for efficient KineTAC-mediated degradation. **a**, Representative western blot showing PD-L1 levels after treatment with 100 nM CXCL12-Atz wild-type or alanine mutants for 24 hr in MDA-MB-231 cells. **b**, PD-L1 levels after treatment with CXCL12-Atz wild-type or CXCL12 N-terminal variants (100 nM) for 24 hr in MDA-MB-231 cells. **c**, Trend of PD-L1 levels to CXCR7 IC50 (nM) of CXCL12 variants after treatment with 100 nM CXCL12-Atz variants for 24 hrs in MDA-MB-231 cells. Wild-type CXCL12 is indicated in red. *N* = 3 biologically independent experiments. **d**, Schematic of EGFR extracellular domains (I-IV) with locations of anti-EGFR antibody epitopes (left) and crystal structure of domain III (PDB: 5SX4) with the epitopes of anti-EGFR binders highlighted in their respective colors. **e**, Trend of EGFR levels to K_D_ after treatment with 100 nM CXCL12-Depa, Nimo, Pani, Neci, Matu, or Ctx for 24 hrs in HeLa cells. *N* = 3 biologically independent experiments. **f**, PD-L1 levels after treatment with 100 nM aglycosylated or glycosylated CXCL12-Atz for 24 hr in MDA-MB-231 cells. **g**, Schematic of CXCL12-Atz Fab fusion construct where CXCL12 chemokine is fused to the N-terminus of atezolizumab Fab via an Avi tag linker. **h**, Multipoint BLI measurement of CXCL12-Atz Fab fusion shows high affinity to PD-L1 Fc fusion. **i**, PD-L1 levels after treatment with 100 nM CXCL12-Atz bispecific or Fab fusion for 24 hr in MDA-MB-231 cells. **j**, Schematic of CXCL12-Atz IgG fusion construct where CXCL12 chemokine is fused to the N-terminus of the heavy chain (HC) or light chain (LC) of atezolizumab IgG via an Avi tag linker. **k**, PD-L1 levels were significantly reduced in MDA-MB-231 cells after 24 hr treatment with 100 nM CXCL12-Atz bispecific (*P* = 0.0222) but not with HC or LC IgG fusions (*P* = 0.2239 and 0.5202, respectively) compared to isotype controls. *N* = 2 biologically independent experiments. Densitometry was used to calculate protein levels and normalized to PBS control. Data are represented as mean values and error bars represent the standard deviation of biological replicates. *P*-values were determined by unpaired two-tailed *t-*tests. Source data

To test how the affinity for target protein PD-L1 affects degradation efficiency, we systematically produced disruptive alanine mutations in the complementary-determining regions of Atz known to contact PD-L1 (ref. ^38^). These mutant Atz variants bound with a wide range of binding affinities (*K*_D_ values from 0.33 to 458 nM) and corresponding kinetic parameters (*k*_on_ and *k*_off_), as measured with BLI (Supplementary Table 2). The Atz mutants were then introduced into the KineTAC scaffold with CXCL12^WT^ and tested for their ability to degrade PD-L1 (Fig. 2c and Extended Data Fig. 4a). We found that degradation inversely correlates with the *K*_d_ (*R*^2^ = 0.638) and even more strongly to dissociation rate (*k*_off_, *R*^2^ = 0.804) but not to the association rate (*k*_on_, *R*^2^ = 0.036; Fig. 2d–f). The wild-type Atz had the highest binding affinity and induced the greatest level of PD-L1 degradation. To test the importance of CXCL12–CXCR7 binding affinity, we produced known N-terminal antagonistic variants of CXCL12 with a range of binding affinities to CXCR7 (*K*_d_ or half-maximum inhibitory concentration (IC_50_) values ranging from 0.014 to 28 nM). These were incorporated into the KineTAC scaffold with Atz and tested for their ability to degrade PD-L1 (Extended Data Fig. 4b)^39^. Remarkably, the degradation potency showed only a weak trend to the IC_50_ against CXCR7 (*R*^2^ = 0.206; Extended Data Fig. 4c). Thus, the levels of degradation appear more dependent on binding affinity of the antibody arm to the target protein than on binding affinity of the CXCL12 variant to CXCR7. Because the effects of these mutations are almost entirely on *k*_off_, this suggests that higher residence time on the target is more important than higher residence time on CXCR7 for robust degradation.

To determine whether a pH-dependent antibody binder against the target protein would affect degradation, BMS936559, a PD-L1 antibody reported to release PD-L1 under acidic conditions (pH < 6.0)^40^, was incorporated into the KineTAC scaffold. The maximal degradation efficiency with CXCL12–BMS936559 was slightly reduced compared to CXCL12–Atz, suggesting that unlike sweeping antibodies, the CXCL12-based KineTAC is not advantaged by low-pH release (Fig. 2g). This result is not due to differences in *K*_d_ because Atz and BMS936559 are reported to have similar binding affinities to PD-L1 (ref. ^41^).

To investigate how the binding epitope on the protein of interest impacts degradation, we generated KineTACs with different HER2- and EGFR-targeting antibodies. For HER2, pertuzumab (Ptz; Perjeta), which binds a distinct epitope from Tras on HER2 (ref. ^42^), was introduced into the KineTAC scaffold (termed CXCL12–Ptz). Following 24-h treatment of MCF7 cells, we found that the IC_50_ for CXCL12–Tras is significantly lower than that for CXCL12–Ptz while reaching the same *D*_max_, indicating that the binding epitope can alter the dose response to KineTACs (Fig. 2h). This effect is not due to differences in binding affinity, as Tras and Ptz have virtually identical *K*_d_ values of 1.43 and 1.92 nM, respectively^43^. For EGFR, we introduced five additional anti-EGFR binders (depatuxizumab (Depa), nimotuzumab (Nimo), panitumumab (Pani; Vectibix), necitumumab (Neci; Portrazza) and matuzumab (Matu))^44^ into the KineTAC scaffold that bind different epitopes on EGFR domains 2 and 3 but have similar affinities (Extended Data Fig. 4d). Following 24-h treatment of HeLa cells, we observed that KineTACs containing Neci, Matu and Pani caused significant degradation of EGFR similar to CXCL12–Ctx, while those containing Depa and Nimo did not substantially degrade EGFR (Fig. 2i). The degradation efficiency of these antibodies was not correlated to their respective binding affinities (*R*^2^ = 0.008; Extended Data Fig. 4e). It is also known that Depa and Nimo bind further from the cell surface than the binders that produced more effective KineTACs. These data highlight the dependence on the binding epitope on the target for KineTAC-mediated degradation.

Glycosylation at N297 in the Fc region can impart greater circulating half-life into IgG1 (ref. ^45^). To test the impact of glycosylation on KineTACs, we introduced N297 into the CXCL12–Atz scaffold and tested the degradation efficiency between the glycosylated and aglycosylated forms. We found that glycosylation at N297 does not substantially impact PD-L1 degradation levels (Extended Data Fig. 4f). Thus, it is possible to incorporate N297 glycosylation for improved pharmacokinetic properties of IgG-containing KineTACs without impacting degradation potency.

Lastly, we determined the sensitivity to orientation of the two binding arms in the KineTAC construct by comparing the parallel Fc-based bispecific to head–tail-linked tandem binders. The latter was produced by fusing CXCL12 to the N terminus of the Atz Fab heavy chain via a flexible avidin tag linker and coexpressing with Atz Fab light chain (Extended Data Fig. 4g). The CXCL12–Atz Fab fusion retained high binding to PD-L1, as measured by BLI (Extended Data Fig. 4h). After 24 h of treatment in MDA-MB-231 cells, the levels of PD-L1 degradation for the tandem Fab fusion construct were substantially less (*D*_max_ = 20%; Extended Data Fig. 4i) than for the Fc-based bispecific (*D*_max_ = 70%; Fig. 1d,e). We also tested an IgG fusion construct where CXCL12 was N-terminally fused to either the heavy chain or light chain of the Fab arm (Extended Data Fig. 4j). The bispecific knob-in-hole IgG construct was significantly better than the bivalent IgG fusions (Extended Data Fig. 4k). Our data suggest that the parallel orientations were more effective than the tandem orientations. Overall, our data highlight that construct geometry is an important variable for KineTAC degradation efficiency.

**Fig. 3:**
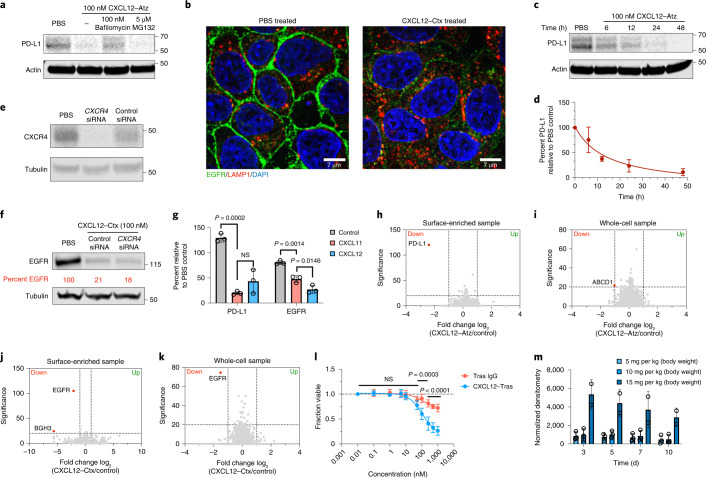
KineTACs mediate target degradation in a highly selective, lysosomal-, time- and CXCR7-dependent manner. **a**, Pretreatment for 1 h with either 100 nM bafilomycin or 5 µM MG132 in MDA-MB-231 cells followed by 24 h of treatment with 100 nM CXCL12–Atz indicates that CXCL12–Atz degrades PD-L1 in a lysosome-dependent manner. **b**, Confocal microscopy images of HeLa cells treated for 24 h with 100 nM CXCL12–Ctx show near complete removal of EGFR. **c**,**d**, Time-course experiment shows increased PD-L1 degradation over time after treatment with 100 nM CXCL12–Atz. **e**, siRNA knockdown of *CXCR4* in HeLa cells after a 48-h transfection. **f**, EGFR levels are unchanged after siRNA knockdown of *CXCR4* followed by 24 h of treatment with 100 nM CXCL12–Ctx in HeLa cells. **g**, PD-L1 in MDA-MB-231 cells or EGFR in HeLa cells is significantly degraded after 24 h of treatment with 100 nM CXCL11–Atz (*P* = 0.0002) or CXCL11–Ctx (*P* = 0.0014) compared to isotype controls. No significant difference was observed between CXCL11–Atz and CXCL12–Atz (*P* = 0.2643), while CXCL12–Ctx caused greater EGFR degradation than CXCL11–Ctx (*P* = 0.0146); *n* = 3 biologically independent experiments. **h**,**i**, Fold change in abundance of surface-enriched (**h**) or whole-cell (**i**) MDA-MB-231 proteins detected using quantitative proteomics analysis after 48 h of treatment with 100 nM CXCL12–Atz reveals highly selective PD-L1 degradation. **j**,**k**, Fold change in abundance of surface-enriched (**j**) or whole-cell (**k**) HeLa proteins detected using quantitative proteomics analysis after 48 h of treatment with 100 nM CXCL12–Ctx reveals highly selective EGFR degradation. Data are the mean of *n* = 2 biological independent experiments and two technical replicates. Proteins showing a greater than twofold change from PBS control with a significance of *P* < 0.01 were considered significantly changed. **l**, In vitro potency of CXCL12–Tras in MDA-MB-175VII cells demonstrates superior cell killing versus Tras IgG; *n* = 2 biologically independent experiments and three technical replicates. **m**, Quantification of CXCL12–Tras plasma levels in male nude mice injected intravenously at 5, 10 or 15 mg per kg (body weight); *n* = 3 different mice per group. Densitometry was used to calculate protein levels, and data were normalized to whole protein levels. Data are represented as mean values, and error bars represent the standard deviation of biological replicates. *P* values for CXCL11 data were determined by one-way ANOVA with Sidak’s multiple comparisons test. *P* values for cell viability assays were determined by unpaired two-tailed *t-*tests at each indicated dose. Source data

We next sought to evaluate the mechanism of KineTAC-mediated degradation. To determine whether KineTACs catalyze degradation via the lysosome or proteasome, MDA-MB-231 cells were pretreated with either medium alone, bafilomycin (an inhibitor of lysosome acidification) or MG132 (a proteasome inhibitor). After 1 h of drug treatment, cells were treated with CXCL12–Atz for 24 h. Bafilomycin pretreatment inhibited degradation of PD-L1, while MG132 had no effect, suggesting that KineTACs mediate degradation via delivery of target proteins to the lysosome (Fig. 3a). Immunofluorescence microscopy revealed virtually complete removal of EGFR from the cell surface following 24 h of CXCL12–Ctx treatment compared to treatment with PBS control, further highlighting that KineTACs induce robust internalization of target proteins (Fig. 3b). KineTAC-mediated degradation occurs in a time-dependent fashion, beginning 6 h after treatment with CXCL12–Atz, with the levels of PD-L1 continuing to decrease over time to near complete degradation by 48 h (Fig. 3c,d).

**Extended Data Fig. 5:**
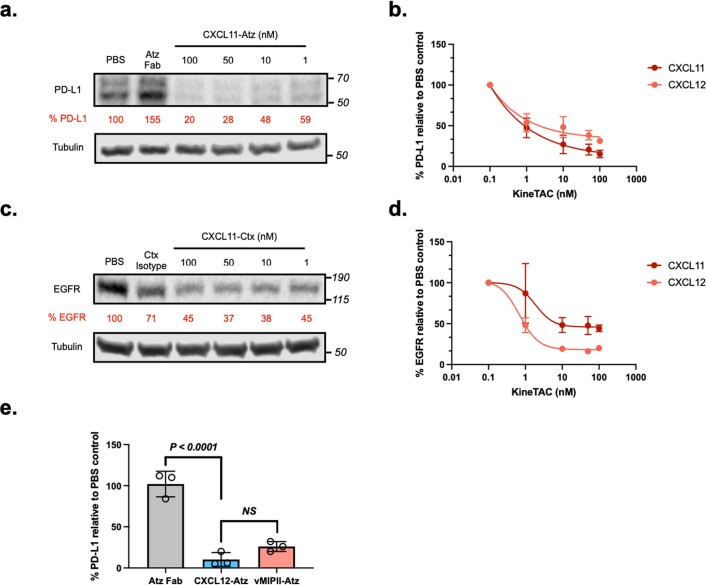
CXCL11 and vMIPII are alternative CXCR7-targeting KineTACs that degrade PD-L1 and EGFR. **a**, Representative western blot showing PD-L1 degradation in MDA-MB-231 cells following 24 hr treatment with various doses of CXCL11-Atz or 100 nM atezolizumab Fab. **b**, Comparison of dose response of PD-L1 degradation in MDA-MB-231 cells following 24 hr treatment with CXCL11- or CXCL12-Atz. **c**, Representative western blot showing EGFR degradation in HeLa cells following 24 hr treatment with various doses of CXCL11-Ctx or 100 nM cetuximab isotype. **d**, Comparison of dose response of EGFR degradation in HeLa cells following 24 hr treatment with CXCL11- or CXCL12-Ctx. **e**, PD-L1 in MDA-MB-231 cells is degraded after 24 hr treatment with 100 nM vMIPII-Atz at levels similar to CXCL12-Atz (*P* = 0.0581). *N* = 3 biologically independent experiments. Data are represented as mean values and error bars represent the standard deviation of biological replicates. *P*-values were determined by one-way ANOVA with Sidak’s multiple comparisons test. Densitometry was used to calculate protein levels and normalized to PBS control. Source data

We next wished to determine which of the two receptors for CXCL12 is more important for degrading surface proteins in the context of KineTACs. We directly probed the role of CXCR4 by using RNA interference to knockdown the levels of *CXCR4* in HeLa cells. After a 48-h transfection with short interfering RNA (siRNA) pools targeting *CXCR4* or a nonspecific control, cells were treated with CXCL12–Ctx for 24 h (Fig. 3e). Western blotting analysis revealed that EGFR degradation levels were unchanged with *CXCR4* knockdown. These data indicate that CXCR4 is not necessary for efficient degradation and suggest that KineTACs operate through CXCR7-mediated internalization (Fig. 3f). Furthermore, KineTACs bearing CXCL11, a chemokine that specifically binds CXCR7 and CXCR3 but not CXCR4 (ref. ^46^), are capable of efficiently degrading both PD-L1 and EGFR (Fig. 3g and Extended Data Fig. 5a–d). A KineTAC containing vMIPII, a viral chemokine that targets CXCR7 along with other chemokine receptors, also efficiently degraded PD-L1 (Extended Data Fig. 5e). Our results suggest that CXCR7 is the prime receptor responsible for KineTAC-mediated degradation and demonstrate the exciting opportunity for using alternative cytokines, such as CXCL11 and vMIPII, in the KineTAC scaffold to degrade target proteins.

Next, we used quantitative mass spectrometry to determine changes across the proteome that occur following KineTAC treatment. Both the surface-enriched and whole-cell lysates were analyzed following 48 h of treatment with the PD-L1-targeting KineTAC CXCL12–Atz or PBS (control) in MDA-MB-231 cells. Remarkably, PD-L1 was virtually the only protein downregulated after CXCL12–Atz treatment compared to after control treatment for the surface-enriched sample (Fig. 3h). Whole-cell proteomics also revealed that no major changes occurred (Fig. 3i and Supplementary Table 3). PD-L1 was not detected in the whole-cell sample, likely due to low abundance of cell surface proteins relative to cytosolic proteins.

**Extended Data Fig. 6:**
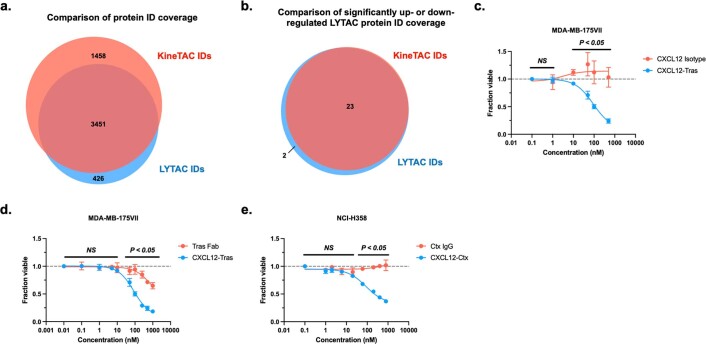
KineTACs are highly selective and functionally active *in vitro*. **a**, Comparison between KineTAC and LYTAC whole cell quantitative proteomics experiments in HeLa cells shows large overlap in total proteins identified. **b**, 23 of 25 proteins that were significantly up- or down-regulated in the LYTAC dataset were identified in the KineTAC whole cell dataset. **c**, *In vitro* potency of CXCL12-Tras in HER2-expressing breast cancer cell line MDA-MB-175VII demonstrates superior cell killing compared to CXCL12 isotype, which is functionally inactive. *N* = 3 biologically independent experiments. **d**, *In vitro* potency of CXCL12-Tras in MDA-MB-175VII cells demonstrates superior cell killing compared to trastuzumab Fab alone. *N* = 3 biologically independent experiments. **e**, *In vitro* potency of CXCL12-Ctx in EGFR-expressing non-small cell lung cancer cell line NCI-H358 demonstrates superior cell killing compared to cetuximab IgG. *N* = 2 biologically independent experiments. Data are represented as mean values and error bars represent the standard deviation of biological replicates. *P*-values were determined by unpaired two-tailed *t-*tests at each indicated dose. Source data

Similar results were observed for HeLa cells treated with the EGFR-targeting KineTAC CXCL12–Ctx compared to cells treated with PBS control (Fig. 3j,k). There were no major changes across the proteome, except for a dramatic reduction in EGFR in both the surface-enriched and whole-cell lysates (Fig. 3j,k and Supplementary Table 4). Moreover, CXCR4 and CXCR7 peptide ID numbers were not altered in the surface-enriched sample, and CXCR4 ID numbers were also unchanged in the whole-cell sample, indicating that treatment with KineTAC does not significantly impact CXCR4 or CXCR7 levels. CXCR7 peptide ID numbers were not identified in the whole-cell sample, likely due to low abundance relative to cytosolic proteins. Furthermore, protein levels of GRB2 and SHC1, which are known interacting partners of EGFR^47,48^, were also not significantly changed. However, we did observe the significant downregulation of ABCD1 and BGH3 in the PD-L1 and EGFR datasets, respectively. These appear to be unrelated to either target protein or CXCR7, suggesting that their reduction was independent of the KineTAC mechanism of action. Together, these data suggest that CXCL12-based KineTACs are highly selective for the target protein without substantially altering levels of the degrading receptor or known interacting partners to EGFR. Interestingly, a previously published proteomics dataset of LYTAC-mediated EGFR degradation identified 25 proteins significantly up- or downregulated following LYTAC treatment^2^. In our HeLa cell dataset, we observed that 89% of the proteins identified in the total LYTAC dataset, including 23 of the 25 proteins seen to significantly change after LYTAC treatment; these 23 proteins were unchanged in the KineTAC dataset (Extended Data Fig. 6a,b). These data suggest that the CXCL12-based KineTAC exhibits more selective EGFR degradation.

Next, we sought to elucidate whether KineTAC-mediated degradation could impart functional cellular consequences. Tras inhibits HER2 function, and MDA-MB-175VII breast cancer cells, which overexpress HER2, will undergo apoptosis after 5 d of treatment with Tras^49^. We compared the abilities of CXCL12–Tras and Tras IgG to kill MDA-MB-175VII cells by using a modified MTT assay. We found that the CXCL12–Tras KineTAC induced significantly greater cell death than Tras IgG, Tras Fab or CXCL12 isotype alone (Fig. 3l and Extended Data Fig. 6c,d). Additionally, non-small cell lung cancer NCI-H358 cells, which overexpress EGFR, undergo significantly greater apoptosis when treated with the CXCL12–Ctx KineTAC than when treated with Ctz IgG (Extended Data Fig. 6e). These data demonstrate that KineTAC-mediated degradation can provide functional advantages over traditional antibody therapeutics, which bind and inhibit but do not degrade.

**Extended Data Fig. 7:**
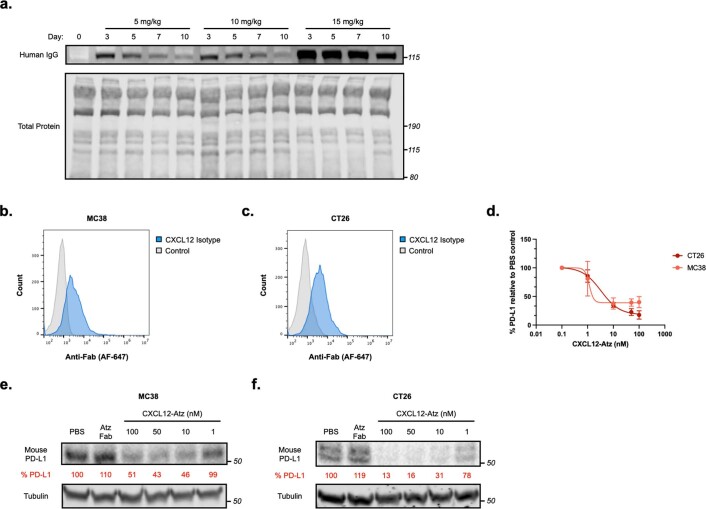
CXCL12-Atz is cross-reactive to mouse cell lines and stable *in vivo*. Flow cytometry showing that human CXCL12 is cross reactive and binds to the surface of **a**, MC38 and **b**, CT26 mouse cell lines. **c**, Dose escalation showing mouse PD-L1 degradation in MC38 and CT26 cells following 24 hr treatment with CXCL12-Atz. Representative western blot of dose escalation showing mouse PD-L1 degradation in **d**, MC38 or **e**, CT26 mouse cells following 24 hr treatment with CXCL12-Atz and mouse IFNg. **f**, Representative western blot showing plasma levels of CXCL12-Tras in male nude mice injected intravenously with 5, 10, or 15 mg/kg. Data are representative of three independent biological replicates or mice. Densitometry was used to calculate protein levels and normalized to PBS control. Data are represented as mean values and error bars represent the standard deviation of biological replicates. Source data

We next asked whether KineTACs would have similar antibody clearance to IgGs in vivo. To this end, male nude mice were injected intravenously with 5, 10 or 15 mg per kg (body weight) CXCL12–Tras (N297 glycosylation present), which is a typical dose range for antibody xenograft studies. Western blotting analysis of plasma antibody levels revealed that the KineTAC remained in plasma up to 10 d after injection with a half-life of 8.7 d, which is comparable to the reported half-life of IgGs in mice (Fig. 3m and Extended Data Fig. 7a)^50^. Given the high homology between human and mouse CXCL12, we tested whether human CXCL12 isotype was cross-reactive. In fact, human CXCL12 isotype bound to mouse cell lines MC38 and CT26 that endogenously express mouse CXCR7 (Extended Data Fig. 7b,c). Together, these results demonstrate that KineTACs have favorable pharmacokinetics and are not being rapidly cleared despite cross-reactivity with mouse CXCR7 receptors. Because Atz is also known to be cross-reactive, the ability of CXCL12–Atz to degrade mouse PD-L1 was tested in both MC38 and CT26 cells. Indeed, CXCL12–Atz mediated near complete degradation of mouse PD-L1 in both cell lines (Extended Data Fig. 7d–f). Thus, we observe a long in vivo half-life of KineTACs despite cross-reactivity and binding to mouse CXCR7 receptors.

**Fig. 4:**
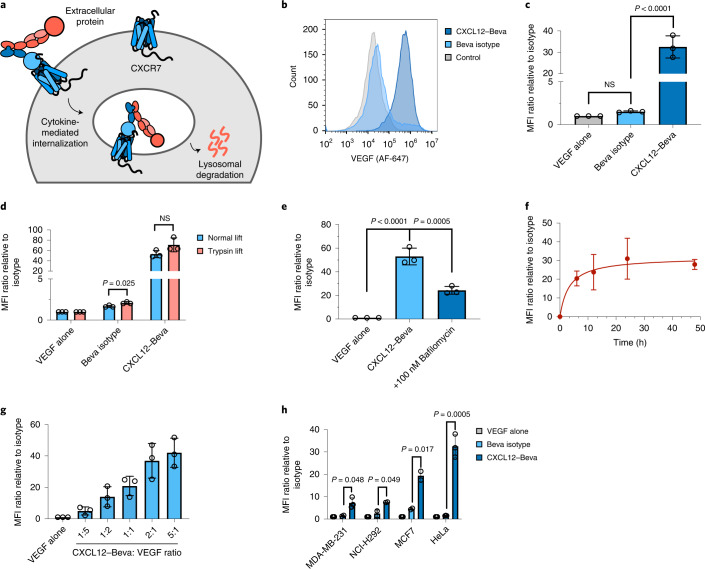
KineTACs enable intracellular uptake of soluble extracellular proteins. **a**, Schematic of KineTAC concept for targeting extracellular proteins for lysosomal degradation. The red ball represents an extracellular protein bound to the KineTAC bi-specific. **b**, Representative flow cytometry results showing a shift in MFI in HeLa cells treated for 24 h with 50 nM CXCL12–Beva and 25 nM VEGF–647 compared to treatment with VEGF alone. **c**, Bar graph of flow cytometry results demonstrating significant uptake of VEGF–647 in HeLa cells following 24 h of treatment with 50 nM CXCL12–Beva compared to treatment with isotype control (*P* < 0.0001). Beva isotype control showed no difference compared to treatment with VEGF–647 alone (*P* = 8421); *n* = 3 biologically independent experiments. **d**, Comparison of HeLa cells lifted with versene (normal lift) or 0.25% trypsin-EDTA (trypsin lift) following 24 h of treatment with 50 nM CXCL12–Beva or isotype controls and 25 nM VEGF–647. No significant change in MFI for CXCL12–Beva treatment (*P* = 0.1121) suggests that fluorescence shift represents accumulation of intracellular VEGF–647 and not simple surface binding; *n* = 3 biologically independent experiments. **e**, Summary of flow cytometry results demonstrating a significant decrease in MFI in HeLa cells following pretreatment with 100 nM bafilomycin and 24 h of treatment with 50 nM CXCL12–Beva and 25 nM VEGF–647 (*P* = 0.0005) compared to no pretreatment with bafilomycin; *n* = 3 biologically independent experiments. **f**, Time-course experiment showing increase in VEGF–647 uptake over time in HeLa cells treated with 50 nM CXCL12–Beva and 25 nM VEGF–647; *n* = 3 biologically independent experiments. **g**, HeLa cells treated for 24 h with varying ratios of CXCL12–Beva to VEGF at constant 25 nM VEGF–647 demonstrate that increasing the KineTAC:VEGF ratio increases VEGF uptake; *n* = 3 biologically independent experiments. **h**, Panel of cell lines for VEGF–647 uptake experiments demonstrating significant VEGF–647 uptake following CXCL12–Beva treatment compared to following treatment with Beva isotype or VEGF alone in MDA-MB-231 (*P* = 0.048), NCI-H292 (*P* = 0.049), MCF7 (*P* = 0.017) and HeLa (*P* = 0.0005) cells; *n* = 3 biologically independent experiments. MFI was measured using live-cell flow cytometry. Data are represented as mean values, and error bars represent the standard deviation of biological replicates. *P* values were determined by unpaired two-tailed *t-*tests or one-way ANOVA with Sidak’s multiple comparisons test. Fold changes are reported relative to incubation with soluble ligand alone. Source data

**Extended Data Fig. 8:**
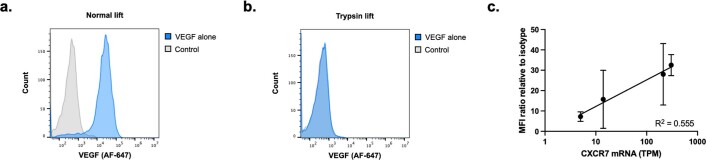
KineTACs mediate uptake of extracellular VEGF. **a**, Representative flow cytometry showing levels of VEGF-647 cell surface labeling after incubation with HeLa cells for 1 hr at 4 °C and normal versene lift. **b**, Representative flow cytometry showing reduction of VEGF-647 cell surface labeling after incubation with HeLa cells for 1 hr at 4 °C and lift with 0.25% trypsin-EDTA (trypsin lift). **c**, Trend of VEGF uptake as calculated by flow cytometry to CXCR7 transcript levels. *N* = 3 biologically independent experiments. Data are represented as mean values and error bars represent the standard deviation of biological replicates. Linear regression analysis using GraphPad Prism was used to calculate the coefficient of determination (R^2^) to determine trend. Data are representative of two biological replicates. Source data

Having demonstrated the ability of KineTACs to mediate cell surface protein degradation, we next asked whether KineTACs could also be applied toward the degradation of soluble extracellular proteins. Antibodies binding to soluble ligands, such as vascular endothelial growth factor (VEGF) and tumor necrosis factor-α (TNF-α), are of tremendous therapeutic importance^51^. Thus, we investigated whether KineTACs could promote cellular uptake of either VEGF or TNF-α (Fig. 4a). VEGF was targeted by incorporating bevacizumab (Beva; Avastin), an FDA-approved VEGF inhibitor, into the KineTAC scaffold (termed CXCL12–Beva). HeLa cells were incubated with Alexa Fluor 647-labeled VEGF (VEGF–647) alone or VEGF–647 plus CXCL12–Beva for 24 h. Following treatment, flow cytometry analysis showed a robust 32-fold increase in cellular fluorescence when VEGF–647 was co-incubated with CXCL12–Beva but not when co-incubated with Beva isotype IgG (Fig. 4b,c). To ensure that the increased cellular fluorescence was due to intracellular uptake of VEGF–647 and not surface binding, we determined the effect of a trypsin lift after treatment, which degrades any cell surface-bound VEGF–647 (Extended Data Fig. 8a,b). We found that there was no significant difference in cellular fluorescence levels between trypsin- and normal-lifted cells treated for 24 h with CXCL12–Beva (Fig. 4d). We did observe a difference between Beva isotype-treated cells, which may reflect slight nonspecific internalization that occurs with the trypsin lift. However, this difference was not observed for CXCL12–Beva. Pretreatment with bafilomycin, a lysosome inhibitor, also impaired the ability of CXCL12–Beva to take up extracellular VEGF–647, as observed by a decrease in cellular fluorescence (Fig. 4e). VEGF–647 uptake was not completely impaired, likely due to bafilomycin blocking trafficking from the endosome to the lysosome, which would still allow some VEGF–647 to be endocytosed but not degraded. Together, these data suggest that KineTACs successfully mediate the intracellular uptake of extracellular VEGF and delivery to the lysosome. KineTAC-mediated uptake of VEGF occurs in a time-dependent manner, similar to membrane protein degradation, with robust internalization beginning at 6 h and reaching steady state by 24 h (Fig. 4f). Furthermore, the levels of VEGF uptake are dependent on the KineTAC:ligand ratio and saturate at ratios greater than 1:1 (Fig. 4g). We next tested the ability of CXCL12–Beva to promote uptake in other cell lines, including breast and lung cancer lines, and found that these cells also significantly take up VEGF (Fig. 4h). Moreover, the extent of uptake was positively correlated with *ACKR3* transcript levels in these cells (*R*^2^ = 0.555; Extended Data Fig. 8c). These data suggest that KineTACs directed against soluble ligands can promote uptake of extracellular soluble ligands and that cell lines expressing higher levels of CXCR7 lead to greater uptake.

**Extended Data Fig. 9:**
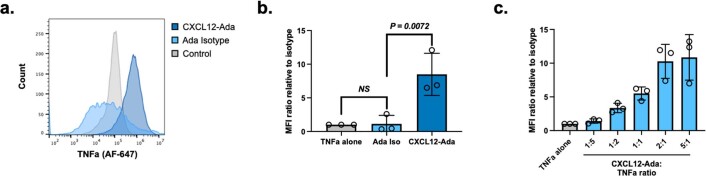
KineTACs mediate the uptake of extracellular TNFa. **a**, Representative flow cytometry showing shift in median fluorescence intensity in HeLa cells treated for 24 hr with 50 nM CXCL12-Ada and 25 nM TNFa-647 compared to TNFa alone. **b**, Summary of flow cytometry demonstrating significant uptake of TNFa-647 in HeLa cells following 24 hr treatment with 50 nM CXCL12-Ada compared to adalimumab isotype (*P* = 0.0072). Adalimumab isotype showed no significant uptake of TNFa-647 compared to TNFa-647 alone (*P* = 0.8701). *N* = 3 biologically independent experiments. **c**, HeLa cells treated for 24 hr with varying ratios of CXCL12-Ada to TNFa, at constant 25 nM TNFa-647, demonstrate that increasing the KineTAC:TNFa ratio increases TNFa uptake. Data are represented as mean values and error bars represent the standard deviation of biological replicates. *P*-values were determined by one-way ANOVA with Sidak’s multiple comparisons test. Fold changes are reported relative to incubation with soluble ligand alone. Source data

We next targeted TNF-α by incorporating adalimumab (Ada; Humira), an FDA-approved TNF-α inhibitor, into the KineTAC scaffold (termed CXCL12–Ada). Following 24 h of treatment in HeLa cells, a significant 8.5-fold increase in cellular fluorescence was observed when TNF-α–647 was co-incubated with CXCL12–Ada compared to when TNF-α–647 was co-incubated with Ada isotype (Extended Data Fig. 9a,b). Consistent with the VEGF uptake experiments, TNF-α uptake was dependent on the KineTAC:ligand ratio (Extended Data Fig. 9c). Thus, KineTACs can robustly mediate the intracellular uptake of soluble ligands, substantially expanding the target scope of KineTAC-mediated targeted degradation.

**Extended Data Fig. 10:**
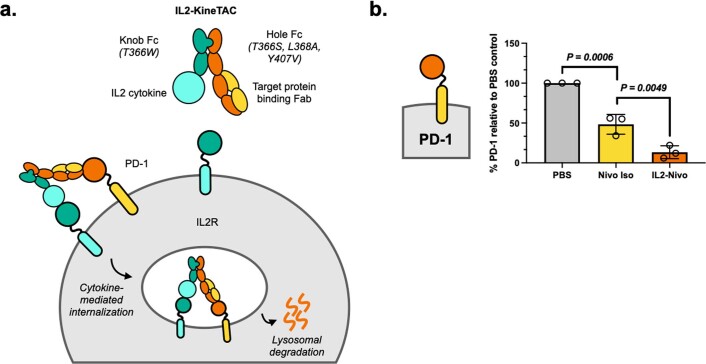
IL2-bearing KineTACs can co-opt IL2R to degrade cell surface PD-1. **a**, Schematic of the KineTAC concept for targeting cell surface proteins for lysosomal degradation via IL2-mediated endocytosis. **b**, Summary of flow cytometry data demonstrating significant degradation of cell surface PD-1 on activated primary human CD8 + T cells following 24 hr treatment with 100 nM IL2-Nivo compared to nivolumab isotype (*P* = 0.0049). % PD-1 was determined by median fluorescence intensity (MFI) of the PE fluorescence channel of live cells. *N* = 3 biologically independent experiments. Data are represented as mean values and error bars represent the standard deviation of biological replicates. *P*-values were determined by one-way ANOVA with Sidak’s multiple comparisons test. Source data

Finally, we asked whether alternative cytokine receptors could be co-opted by KineTACs to mediate the clearance of target proteins. We hypothesized that KineTACs bearing interleukin-2 (IL-2) cytokine could engage the IL-2 receptor complex (IL-2R), which comprises CD25, IL-2RB and IL-2RG, as a degrading receptor. CD25 is known to internalize and recycle back to the cell surface after heterotrimer formation, while IL-2RB and IL-2RG are internalized and degraded via the lysosome after IL-2 binding (Extended Data Fig. 10a)^52^. We generated knob-in-hole KineTACs in which human IL-2 cytokine was N-terminally fused to the knob IgG1 Fc domain, and the hole Fc arm contained the Fab antibody sequence for Nivo, which binds PD-1. Activated T cells were then treated for 24 h with the IL-2-bearing PD-1 KineTAC (termed IL-2–Nivo). Following treatment with IL-2–Nivo, cell surface PD-1 levels were significantly reduced, with a *D*_max_ of 86.7%, compared to those observed after treatment with Nivo isotype control (Extended Data Fig. 10b). These data suggest that other cytokines can be co-opted with KineTACs, expanding the repertoire for cell-selective targeted protein degradation. We note that bispecific constructs currently called immunocytokines link a cytokine to a targeting antibody with the intent to deliver cytokines such as IL-2 to T cells in the tumor microenvironment. In addition to selective cytokine delivery, our data suggest that these may also inadvertently degrade the target protein if it is also present on the T cells, which could be an advantage in targeting PD-1 with an IL-2-bearing KineTAC.

## Discussion

In summary, our studies show that KineTACs are a versatile and modular targeted degradation platform that can enable robust lysosomal degradation of both cell surface and extracellular proteins on various cell types. We find that KineTAC-mediated degradation is driven by recruitment of both the recycling receptor CXCR7 and the target protein. Factors such as binding affinity, epitope, construct design and ratio of degrading receptor to target protein can substantially affect efficiency. Other factors, such as signaling competence and pH dependency for the protein of interest, did not substantially impact degradation in the case of CXCL12-bearing KineTACs. These results provide valuable insights into how to engineer, optimize and expand effective KineTACs going forward. We further show that CXCL12-based KineTACs operate via time, lysosome and CXCR7 dependence and are exquisitely selective in degrading target proteins with minimal off-target effects. Experiments with alternative cytokines CXCL11, vMIPII and IL-2 highlight the versatility and generalizability of the KineTAC platform to co-opting alternative cytokine receptors as degraders. KineTACs are built from simple genetically encoded parts, do not require complex synthesis or bioconjugation for production and are more easily expressed than two Fab-based bispecifics having different light chains. Given differences in selectivity and target scope that we and others have observed between degradation pathways, there is an ongoing need to co-opt novel receptors for lysosomal degradation, such as CXCR7, that may offer advantages in terms of tissue selectivity or degradation efficiency. Thus, we anticipate that the KineTAC platform will impact targeted protein degradation for targeting the extracellular proteome for both therapeutic and research applications.

## Methods

### Cell lines

Cell lines were grown and maintained in T75 (Thermo Fisher Scientific) flasks at 37 °C and 5% CO_2_. MDA-MB-231, MDA-MB-175VII, HeLa, A431 and HCC827 cells were grown in DMEM supplemented with 10% fetal bovine serum (FBS) and 1% penicillin/streptomycin. MCF7, NCI-H292, NCI-H1975 and NCI-H358 cells were grown in RPMI-1640 supplemented with 10% FBS and 1% penicillin/streptomycin. SK-BR-3 cells were grown in McCoy’s 5A supplemented with 10% FBS and 1% penicillin/streptomycin. A549 cells were grown in F12K supplemented with 10% FBS and 1% penicillin/streptomycin.

### Protein expression

Fabs were expressed in *Escherichia coli* C43 (DE3) Pro+ grown in an optimized TB autoinduction medium at 37 °C for 6 h, cooled to 30 °C for 18 h and purified by Protein A affinity chromatography. IgGs and bispecifics were expressed and purified from Expi293 BirA cells using transient transfection (Expifectamine, Thermo Fisher Scientific). Enhancers were added 20 h after transfection. Cells were incubated for 5 d at 37 °C and 8% CO_2_. Medium was then collected by centrifugation at 4,000*g* for 20 min. IgGs were purified by Protein A affinity chromatography, buffer exchanged into PBS by spin concentration and flash frozen for storage at −80 °C. Bispecifics were purified by Ni-NTA affinity chromatography, buffer exchanged into PBS containing 20% glycerol, concentrated and flash frozen for storage at −80 °C. Purity and integrity of all proteins were assessed by SDS–PAGE.

### BLI

BLI data were measured using an Octet RED384 (ForteBio) instrument using ForteBio Octet data acquisition software (v12.0.2.11). Biotinylated antigens were immobilized on a streptavidin biosensor and loaded until 0.4-nm signal was achieved. After blocking with 10 µM biotin, purified antibodies in solution were used as the analyte. PBS + 0.05% Tween + 0.2% bovine serum albumin (BSA) was used for all buffers. Data were analyzed using the ForteBio Octet data analysis software (v12.0), and kinetic parameters were determined using a 1:1 monovalent binding model.

### Degradation experiments

Cells were plated in 6- or 12-well plates and grown to ~70% confluency before treatment. Medium was aspirated, and cells were treated with bispecifics or control antibodies in complete growth medium. For soluble ligand uptake experiments, biotinylated soluble ligand was preincubated with streptavidin–647 at 37 °C for 30 min, mixed with bispecific or control antibodies and added to cells. After incubation at 37 °C for the designated amount of time, cells were washed with PBS, lifted with versene and collected by centrifugation at 300*g* for 5 min at 4 °C. Samples were then tested by western blotting or flow cytometry to quantify protein levels.

### Western blotting

Cell pellets were lysed with 1× RIPA buffer containing cOmplete mini protease inhibitor cocktail (Sigma-Aldrich) at 4 °C for 40 min. Lysates were centrifuged at 16,000*g* for 10 min at 4 °C, and protein concentrations were normalized by bicinchoninic acid assay (Pierce). Then, 4× NuPAGE LDS sample buffer (Invitrogen) and 2-mercaptoethanol were added to the lysates and boiled for 10 min. Equal amounts of lysates were loaded onto a 4–12% Bis-Tris gel and run at 200 V for 37 min. The gel was incubated in 20% ethanol for 10 min and transferred onto a polyvinylidene difluoride membrane. The membrane was blocked in PBS with 0.1% Tween-20 + 5% BSA for 30 min at room temperature with gentle shaking. Membranes were incubated overnight with primary antibodies at respective dilutions at 4 °C with gentle shaking in PBS + 0.2% Tween-20 + 5% BSA. Membranes were washed four times with TBS + 0.1% Tween-20 and co-incubated with horseradish peroxidase–anti-rabbit IgG (Cell Signaling Technologies, 7074A; 1:2,000) and 680RD goat anti-mouse IgG (LI-COR, 926-68070; 1:10,000) in PBS + 0.2% Tween-20 + 5% BSA for 1 h at room temperature. Membranes were washed four times with TBS + 0.1% Tween-20 and then washed with PBS. Membranes were imaged using an OdysseyCLxImager (LI-COR). SuperSignal West Pico Plus chemiluminescent substrate (Thermo Fisher Scientific) was then added and imaged using a ChemiDoc Imager (Bio-Rad). Band intensities were quantified using Image Studio software (LI-COR, v5.2.5).

### Primary human CD8^+^ T cell isolation

Primary human T cells were isolated from leukoreductin chamber residuals following Trima Apheresis (Blood Centers of the Pacific) using established protocols^53^. Briefly, PBMCs were isolated by Ficoll separation in SepMate tubes (STEMCELL Technologies) according to manufacturer’s instructions. EasySep Human CD8^+^ T cell isolation kits (STEMCELL Technologies) were then used to isolate CD8^+^ T cells from PBMCs following the manufacturer’s protocol. Isolated cells were then analyzed for purity by flow cytometry on a Beckman Coulter CytoFlex flow cytometer using anti-CD3, anti-CD4 and anti-CD8a (BioLegend). CytExpert software (v2.3.1.22) was used for data acquisition, and FlowJo (v10.8.0) was used for data analysis.

### CD8^+^ T cell activation

Following CD8^+^ T cell isolation, cells were stimulated with recombinant IL-2 (GoldBio) and IL-15 (GoldBio) and ImmunoCult Human CD3/CD28 T cell Activation (STEMCELL Technologies) for 4 d at 37 °C. Activated CD8^+^ T cells were then analyzed for the upregulation of activation markers CD25 and PD-1 by flow cytometry using anti-CD25 and anti-PD-1 (BioLegend). Once activation was confirmed, cells were dosed as described above, and levels of target protein were analyzed by flow cytometry on a Beckman Coulter CytoFlex flow cytometer. CytExpert software (v2.3.1.22) was used for data acquisition, and FlowJo (v10.8.0) was used for data analysis.

### Flow cytometry

Cell pellets were washed with cold PBS and centrifuged at 300*g* for 5 min. Cells were blocked with cold PBS + 3% BSA and centrifuged (300*g* for 5 min). Cells were incubated with primary antibodies diluted in PBS + 3% BSA for 30 min at 4 °C. Cells were washed three times with cold PBS + 3% BSA, and secondary antibodies (if applicable) diluted in PBS + 3% BSA were added and incubated for 30 min at 4 °C. Cells were washed three times with cold PBS + 3% BSA and resuspended in cold PBS. Flow cytometry was performed on a CytoFLEX cytometer (Beckman Coulter) using CytExpert software (v2.3.1.22) for data acquisition. Gating was performed on single cells and live cells before acquisition of 10,000 cells. Analysis was performed using FlowJo software (v10.8.0).

### Flow cytometry for soluble ligand uptake

Cell pellets were washed three times with cold PBS and centrifuged at 300*g* for 5 min. Cells were then resuspended in cold PBS. Flow cytometry was performed on a CytoFLEX cytometer (Beckman Coulter) using CytExpert software (v2.3.1.22) for data acquisition. Gating was performed on single cells and live cells before acquisition of 10,000 cells. Analysis was performed using FlowJo software (v10.8.0).

### Trypsin lift for soluble ligand uptake

Following 24-h dosing of cells with soluble ligand and bispecific antibodies at 37 °C, cells were washed with cold PBS two times. Cells were then lifted with either versene (normal lift) or 0.25% trypsin-EDTA (trypsin lift) at 37 °C for 3 min and collected by centrifugation at 300*g* for 5 min. Cells were washed three times with cold PBS and centrifuged at 300*g* for 5 min before resuspension in cold PBS. Flow cytometry was performed on a CytoFLEX cytometer (Beckman Coulter) using CytExpert software (v2.3.1.22) for data acquisition. Gating was performed on single cells and live cells before acquisition of 10,000 cells. Analysis was performed using FlowJo software (v10.8.0).

### Confocal microscopy

Cells were plated onto Mat-Tek 35-mm glass-bottom Petri dishes pretreated with poly-d-lysine and grown to ~70% confluency before treatment. Medium was aspirated, and cells were treated with bispecifics or control antibodies in complete growth medium. For soluble ligand uptake experiments, biotinylated soluble ligand was preincubated with streptavidin–647 at 37 °C for 30 min, mixed with bispecific or control antibodies and added to cells. After 24 h of incubation at 37 °C, medium was aspirated, and cells were washed with PBS. Cells were then stained using standard protocols for LysoTracker Deep Red (Invitrogen), DAPI (Cell Signaling Technologies) and primary antibody. Samples were imaged using a Nikon Ti Microscope with a Yokogawa CSU-22 spinning disk confocal and a ×100 objective lens; 405-, 488- and 647-nm lasers were used to image DAPI, primary antibody and LysoTracker, respectively. Images were deconvoluted and processed using NIS-Element (v5.21.03) and Fiji software (v2.1.0) packages.

### siRNA knockdown

HeLa cells were plated in a six-well plate and grown to confluency. Cells were transfected with 20 pmol of siRNA (ON-TARGETplus siRNA SMARTPool, Dharmacon) and DharmaFECT 4 reagent (Dharmacon) according to manufacturer’s instructions. Cells were incubated for 48–72 h at 37 °C and 5% CO_2_, and siRNA knockdown was validated by western blotting.

### Cell culture/stable isotope labeling using amino acids in cell culture (SILAC) labeling and treatment

MDA-MB-231 cells were grown in DMEM for SILAC (Thermo Fisher) with 10% dialyzed FBS (Gemini). Medium was also supplemented with either light l-[^12^C_6_,^14^N_2_]-lysine/l-[^12^C_6_,^14^N_4_]-arginine (Sigma) or heavy l-[^13^C_6_,^15^N_2_]-lysine/L-[^13^C_6_,^15^N_4_]-arginine (Cambridge Isotope Laboratories). Cells were maintained in SILAC medium for five passages to ensure complete isotopic labeling. Cells were then treated with either PBS control or 100 nM bispecific for 48 h before cells were collected, and heavy/light-labeled cells were mixed at a 1:1 ratio in both forward and reverse mode. A small portion of these cells was set aside for whole-cell proteomic analysis, and the remainder was used to prepare surface-proteome enrichment.

### Mass spectrometry sample preparation

Cell surface glycoproteins were captured largely as previously described^54^ but using a modified protocol to facilitate small sample input. Briefly, cells were first washed in PBS (pH 6.5) before the glycoproteins were oxidized with 1.6 mM sodium periodate (Sigma) in PBS (pH 6.5) for 20 min at 4 °C. Cells were then biotinylated via the oxidized vicinal diols with 1 mM biocytin hydrazide (Biotium) in the presence of 10 mM aniline (Sigma) in PBS (pH 6.5) for 90 min at 4 °C. Cell pellets were lysed with a 2× dilution of commercial RIPA buffer (Millipore) supplemented with 1× protease inhibitor cocktail (Sigma) and 2 mM EDTA (Sigma) for 10 min at 4 °C. Cells were further disrupted with probe sonication, and the cell lysates were then incubated with NeutrAvidin-coated agarose beads (Thermo) in Poly-Prep chromatography columns (Bio-Rad) for 2 h at 4 °C to isolate biotinylated glycoproteins. After this incubation, cells were washed sequentially with 1× RIPA (Millipore) plus 1 mM EDTA, high-salt PBS (PBS (pH 7.4) and 2 M NaCl (Sigma)) and denaturing urea buffer (50 mM ammonium bicarbonate and 2 M urea). All wash buffers were heated to 42 °C before use. Proteins on the beads were next digested and desalted using the Preomics iST mass spectrometry sample preparation kit (Preomics) per the manufacturer’s recommendations with few modifications. First, samples were resuspended in the ‘LYSE’ solution and transferred to fresh tubes. After incubating in LYSE for 10 min at 55 °C, the ‘DIGEST’ solution was added, and beads were incubated for 90 min at 37 °C with mixing at 500 r.p.m. Following on-bead digest, the peptide eluate was isolated using SnapCap spin columns (Pierce), and the ‘STOP’ solution was added. The sample was then transferred to the Preomics cartridge and desalted using the manufacturer’s protocol. Samples were dried, resuspended in 0.1% formic acid and 2% acetonitrile (Fisher) and quantified using the Pierce peptide quantification kit before liquid chromatography–tandem mass spectrometry analysis. Whole-cell lysate samples were prepared using the Preomics kit protocol for whole-lysate samples. Resulting peptides were dried and quantified in the same manner as the surface-enriched samples.

### Mass spectrometry

Liquid chromatography–tandem mass spectrometry was performed using a Bruker NanoElute chromatography system coupled to a Bruker timsTOF Pro mass spectrometer. Peptides were separated using a prepacked IonOpticks Aurora (25 cm × 75 μm) C18 reversed-phase column (1.6-µm pore size, Thermo) fitted with a CaptiveSpray emitter for the timsTOF Pro CaptiveSpray source. For all samples, 200 ng of resuspended peptides was injected and separated using a linear gradient of 2–23% solvent B (solvent A: 0.1% formic acid and 2% acetonitrile; solvent B: acetonitrile with 0.1% formic acid) over 90 min at 400 µl min^–1^ with a final ramp to 34% B over 10 min. Separations were performed at a column temperature of 50 °C. Data-dependent acquisition was performed using a timsTOF PASEF tandem mass spectrometry method (TIMS mobility scan range of 0.70–1.50 V•s cm^–2^, mass scan range of 100–1,700 *m*/*z*, ramp time of 100 ms, 10 PASEF scans per 1.17 s, active exclusion of 24 s, charge range of 0–5 and minimum MS1 intensity of 500). The normalized collision energy was set at 20.

### Data analysis/statistics

SILAC proteomics data were analyzed using PEAKSOnline (v1.5). For all samples, searches were performed with a precursor mass error tolerance of 20 ppm and a fragment mass error tolerance of 0.03 Da. The digest was considered semispecific, and up to three missed cleavages were allowed. For whole-cell proteome data, the reviewed SwissProt database for the human proteome (downloaded 12 December 2020) was used. For surface-enriched samples, a database composed of SwissProt proteins annotated ‘plasma membrane’ was used to ensure accurate unique peptide identification for surface proteins. Carbamidomethylation of cysteine was used as a fixed modification, whereas the isotopic labels for arginine and lysine, acetylation of the N terminus, oxidation of methionine and deamidation of asparagine and glutamine were set as variable modifications. Only peptide-spectrum matches (PSMs) and protein groups with a false discovery rate of less than 1% were considered for downstream analysis. SILAC analysis was performed using the forward and reverse samples, and at least two labels for the ID and features were required. Proteins showing a greater than twofold change from PBS control with a significance of *P* < 0.01 were considered to be significantly changed.

### Cell viability experiments

Cell viability assays were performed using an MTT modified assay. Briefly, on day 0, 15,000 MDA-MB-175VII or 7,000 NCI-H358 cells were plated in each well of a 96-well plate. On day 1, bispecifics or control antibodies were added in a dilution series. Cells were incubated at 37 °C and 5% CO_2_ for 5 d. On day 6, 40 µl of 2.5 mg ml^–1^ thiazolyl blue tetrazolium bromide (GoldBio) was added to each well and incubated at 37 °C and 5% CO_2_ for 4 h; 100 µl of 10% SDS in 0.01 M HCl was then added to lyse cells and release MTT product. After 4 h at room temperature, absorbance at 600 nm was quantified using an Infinite M200 PRO plate reader (Tecan). Data were plotted using GraphPad Prism software (v9.2.0), and curves were generated using non-linear regression with sigmoidal 4PL parameters.

### Antibody in vivo stability study

Male nude nu/nu mice (8–10 weeks old, bred at the University of California San Francisco (UCSF) MZ Breeding Facility) were treated with 5, 10 or 15 mg per kg (body weight) CXCL12–Tras via intravenous injection (three mice per group). Blood was collected from the lateral saphenous vein using EDTA capillary tubes at day 0 before intravenous injection and at days 3, 5, 7 and 10 after injection. Plasma was separated after centrifugation at 700*g* at 4 °C for 15 min. To determine the levels of CXCL12–Tras, 1 µl of plasma was diluted into 30 µl of NuPAGE LDS sample buffer (Invitrogen), loaded onto a 4–12% Bis-Tris gel and run at 200 V for 37 min. The gel was incubated in 20% ethanol for 10 min and transferred onto a polyvinylidene difluoride membrane. The membrane was washed with water followed by incubation for 5 min with REVERT 700 total protein stain (LI-COR). The blot was then washed twice with REVERT 700 wash solution (LI-COR) and imaged using an OdysseyCLxImager (LI-COR). The membrane was then blocked in PBS with 0.1% Tween-20 + 5% BSA for 30 min at room temperature with gentle shaking. Membranes were incubated overnight with 800 CW goat anti-human IgG (LI-COR, 1:10,000) at 4 °C with gentle shaking in PBS + 0.2% Tween-20 + 5% BSA. Membranes were washed four times with TBS + 0.1% Tween-20 and washed with PBS. Membranes were imaged using an OdysseyCLxImager (LI-COR). Band intensities were quantified using Image Studio Software (LI-COR, v5.2). All animal care and experimentation were conducted in full accordance with UCSF Institutional Animal Care and Use Committee protocol AN179937.

### Reporting summary

Further information on research design is available in the Nature Research Reporting Summary linked to this article.

## Online content

Any methods, additional references, Nature Research reporting summaries, source data, extended data, supplementary information, acknowledgements, peer review information; details of author contributions and competing interests; and statements of data and code availability are available at 10.1038/s41587-022-01456-2.

## Supplementary information

Supplementary Information Supplementary Tables 1 and 2, representative flow cytometry gating strategy figure and antibodies and detection reagents table.

Reporting Summary

Supplementary Table 3 Full dataset for quantitative proteomics of whole-cell and cell surface MDA-MB-231 cells after CXCL12–Atz treatment.

Supplementary Table 4 Full dataset for quantitative proteomics of whole-cell and cell surface HeLa cells after CXCL12–Ctx treatment.

## Data Availability

Quantitative proteomics data for PD-L1 and EGFR degradation experiments are provided in Supplementary Tables 3 and 4, respectively. All other raw data supporting the results are available upon reasonable request to the corresponding author. Source data are provided with this paper.

## Source data

Source Data Fig. 1 Statistical source data.
Source Data Fig. 1 Full-length, unprocessed gels or blots.
Source Data Fig. 2 Statistical source data.
Source Data Fig. 2 Full-length, unprocessed gels or blots.
Source Data Fig. 3 Statistical source data.
Source Data Fig. 3 Full-length, unprocessed gels or blots.
Source Data Fig. 4 Statistical source data.
Source Data Extended Data Fig. 1 Full-length, unprocessed gels or blots.
Source Data Extended Data Fig. 2 Statistical source data.
Source Data Extended Data Fig. 2 Full-length, unprocessed gels or blots.
Source Data Extended Data Fig. 4 Statistical source data.
Source Data Extended Data Fig. 4 Full-length, unprocessed gels or blots.
Source Data Extended Data Fig. 5 Statistical source data.
Source Data Extended Data Fig. 5 Full-length, unprocessed gels or blots.
Source Data Extended Data Fig. 6 Statistical source data.
Source Data Extended Data Fig. 7 Statistical source data.
Source Data Extended Data Fig. 7 Full-length, unprocessed gels or blots.
Source Data Extended Data Fig. 8 Statistical source data.
Source Data Extended Data Fig. 9 Statistical source data.
Source Data Extended Data Fig. 10 Statistical source data.

## References

[1] Cotton AD, Nguyen DP, Gramespacher JA, Seiple IB, Wells JA (2021). Development of antibody-based PROTACs for the degradation of the cell-surface immune checkpoint protein PD-L1. J. Am. Chem. Soc..

[2] Banik SM (2020). Lysosome-targeting chimaeras for degradation of extracellular proteins. Nature.

[3] Caianiello DF (2021). Bifunctional small molecules that mediate the degradation of extracellular proteins. Nat. Chem. Biol..

[4] Zhou Y, Teng P, Montgomery NT, Li X, Tang W (2021). Development of triantennary *N*-acetylgalactosamine conjugates as degraders for extracellular proteins. ACS Cent. Sci..

[5] Pettersson M, Crews CM (2019). PROteolysis TArgeting Chimeras (PROTACs)—past, present and future. Drug Discov. Today Technol..

[6] Jang J (2020). Mutant-selective allosteric EGFR degraders are effective against a broad range of drug-resistant mutations. Angew. Chem. Int. Ed..

[7] Ma L (2021). Abstract 1265: Discovery of GT19077, a c-Myc/Max protein–protein Interaction (PPI) small molecule inhibitor, and GT19506 a c-Myc PROTAC molecule, for targeting c-Myc-driven blood cancers and small cell lung cancers. Cancer Res..

[8] Lee GT (2021). Effects of MTX-23, a novel PROTAC of androgen receptor splice variant-7 and androgen receptor, on CRPC resistant to second-line antiandrogen therapy. Mol. Cancer Ther..

[9] Fu L (2021). Discovery of highly potent and selective IRAK1 degraders to probe scaffolding functions of IRAK1 in ABC DLBCL. J. Med. Chem..

[10] Zhang J (2020). Assessing IRAK4 functions in ABC DLBCL by IRAK4 kinase inhibition and protein degradation. Cell Chem. Biol..

[11] Sakamoto KM (2001). PROTACs: chimeric molecules that target proteins to the Skp1–Cullin–F box complex for ubiquitination and degradation. Proc. Natl Acad. Sci. USA.

[12] Krönke J (2014). Lenalidomide causes selective degradation of IKZF1 and IKZF3 in multiple myeloma cells. Science.

[13] Mullard, A. First targeted protein degrader hits the clinic. *Nat. Rev. Drug Discov*. 10.1038/d41573-019-00043-6 (2019). 10.1038/d41573-019-00043-6 30936511

[14] Dovedi SJ (2021). Design and efficacy of a monovalent bispecific PD-1/CTLA4 antibody that enhances CTLA4 blockade on PD-1^+^ activated T cells. Cancer Discov..

[15] Jin H (2021). Avelumab internalization by human circulating immune cells is mediated by both Fc γ receptor and PD-L1 binding. OncoImmunology.

[16] Igawa T (2010). Antibody recycling by engineered pH-dependent antigen binding improves the duration of antigen neutralization. Nat. Biotechnol..

[17] Igawa T, Haraya K, Hattori K (2016). Sweeping antibody as a novel therapeutic antibody modality capable of eliminating soluble antigens from circulation. Immunol. Rev..

[18] Ahn G (2021). LYTACs that engage the asialoglycoprotein receptor for targeted protein degradation. Nat. Chem. Biol..

[19] Janssens R, Struyf S, Proost P (2018). The unique structural and functional features of CXCL12. Cell. Mol. Immunol..

[20] Costantini S, Raucci R, De Vero T, Castello G, Colonna G (2013). Common structural interactions between the receptors CXCR3, CXCR4 and CXCR7 complexed with their natural ligands, CXCL11 and CXCL12, by a modeling approach. Cytokine.

[21] Freeman GJ (2000). Engagement of the PD-1 immunoinhibitory receptor by a novel B7 family member leads to negative regulation of lymphocyte activation. J. Exp. Med..

[22] Ridgway JBB, Presta LG, Carter P (1996). ‘Knobs-into-holes’ engineering of antibody CH3 domains for heavy chain heterodimerization. Protein Eng..

[23] Joshi KK (2019). Elucidating heavy/light chain pairing preferences to facilitate the assembly of bispecific IgG in single cells. mAbs.

[24] Ovacik AM (2019). Single cell-produced and in vitro-assembled anti-FcRH5/CD3 T-cell dependent bispecific antibodies have similar in vitro and in vivo properties. mAbs.

[25] Lim SA (2021). Bispecific VH/Fab antibodies targeting neutralizing and non-neutralizing spike epitopes demonstrate enhanced potency against SARS-CoV-2. mAbs.

[26] Douglass EF, Miller CJ, Sparer G, Shapiro H, Spiegel DA (2013). A comprehensive mathematical model for three-body binding equilibria. J. Am. Chem. Soc..

[27] Wang J, Xu B (2019). Targeted therapeutic options and future perspectives for HER2-positive breast cancer. Signal Transduct. Target. Ther..

[28] Barretina J (2012). The Cancer Cell Line Encyclopedia enables predictive modeling of anticancer drug sensitivity. Nature.

[29] Sharma SV, Bell DW, Settleman J, Haber DA (2007). Epidermal growth factor receptor mutations in lung cancer. Nat. Rev. Cancer.

[30] Spano J-P (2005). Impact of EGFR expression on colorectal cancer patient prognosis and survival. Ann. Oncol..

[31] Arienti, C., Pignatta, S. & Tesei, A. Epidermal Growth Factor Receptor Family and its Role in Gastric Cancer. *Front. Oncol*. **9**, 1308 (2019). 10.3389/fonc.2019.01308 PMC6901979 31850207

[32] Martinko AJ (2018). Targeting RAS-driven human cancer cells with antibodies to upregulated and essential cell-surface proteins. eLife.

[33] Goldenberg DM, Stein R, Sharkey RM (2018). The emergence of trophoblast cell-surface antigen 2 (TROP-2) as a novel cancer target. Oncotarget.

[34] Hsu E-C (2020). Trop2 is a driver of metastatic prostate cancer with neuroendocrine phenotype via PARP1. Proc. Natl Acad. Sci. USA.

[35] Saad EB, Oroya A, Rudd CE (2020). Abstract 6528: Anti-PD-1 induces the endocytosis of the co-receptor from the surface of T-cells: nivolumab is more effective than pembrolizumab. Cancer Res..

[36] Chatterjee S, Azad BB, Nimmagadda S (2014). The intricate role of CXCR4 in cancer. Adv. Cancer Res..

[37] Stephens, B. S., Ngo, T., Kufareva, I. & Handel, T. M. Functional anatomy of the full-length CXCR4–CXCL12 complex systematically dissected by quantitative model-guided mutagenesis. *Sci. Signal*. **13**, eaay5024 (2020). 10.1126/scisignal.aay5024 PMC7437921 32665413

[38] Lee HT (2017). Molecular mechanism of PD-1/PD-L1 blockade via anti-PD-L1 antibodies atezolizumab and durvalumab. Sci Rep..

[39] Hanes MS (2015). Dual targeting of the chemokine receptors CXCR4 and ACKR3 with novel engineered chemokines. J. Biol. Chem..

[40] Liu H (2020). Identification of a hotspot on PD-L1 for pH-dependent binding by monoclonal antibodies for tumor therapy. Signal Transduct. Target. Ther..

[41] Tan S (2018). Distinct PD-L1 binding characteristics of therapeutic monoclonal antibody durvalumab. Protein Cell.

[42] Fuentes G, Scaltriti M, Baselga J, Verma CS (2011). Synergy between trastuzumab and pertuzumab for human epidermal growth factor 2 (Her2) from colocalization: an in silico based mechanism. Breast Cancer Res..

[43] Fu W (2014). Insights into HER2 signaling from step-by-step optimization of anti-HER2 antibodies. mAbs.

[44] Cai W-Q (2020). The latest battles between EGFR monoclonal antibodies and resistant tumor cells. Front. Oncol..

[45] Jefferis R (2009). Glycosylation as a strategy to improve antibody-based therapeutics. Nat. Rev. Drug Discov..

[46] Naumann U (2010). CXCR7 functions as a scavenger for CXCL12 and CXCL11. PLoS ONE.

[47] Yamazaki T (2002). Role of Grb2 in EGF-stimulated EGFR internalization. J. Cell Sci..

[48] Zheng Y (2013). Temporal regulation of EGF signalling networks by the scaffold protein Shc1. Nature.

[49] Ginestier C (2007). ERBB2 phosphorylation and trastuzumab sensitivity of breast cancer cell lines. Oncogene.

[50] Vieira P, Rajewsky K (1988). The half-lives of serum immunoglobulins in adult mice. Eur. J. Immunol..

[51] Attwood MM, Jonsson J, Rask-Andersen M, Schiöth HB (2020). Soluble ligands as drug targets. Nat. Rev. Drug Discov..

[52] Hémar A (1995). Endocytosis of interleukin 2 receptors in human T lymphocytes: distinct intracellular localization and fate of the receptor α, β, and γ chains. J. Cell Biol..

[53] Roth TL (2018). Reprogramming human T cell function and specificity with non-viral genome targeting. Nature.

[54] Leung KK (2019). Multiomics of azacitidine-treated AML cells reveals variable and convergent targets that remodel the cell-surface proteome. Proc. Natl Acad. Sci. USA.

