# Supplementary material for: Modular cytokine receptor-targeting chimeras for targeted degradation of cell surface and extracellular proteins
Source: Nat Biotechnol. 2022 Sep 22;41(2):273–81. doi: 10.1038/s41587-022-01456-2 (PMC9931583; doi:10.1038/s41587-022-01456-2)
Supplement: Supplementary file 1 — Supplementary Tables 1 and 2, representative flow cytometry gating strategy figure and antibodies and detection reagents table. [file 41587_2022_1456_MOESM1_ESM.pdf]

nature portfolio

https://doi.org/10.1038/s41587-022-01456-2

Supplementary information

Modular cytokine receptor-targeting chimeras for targeted degradation of cell surface and extracellular proteins

In the format provided by the authors and unedited

Supplementary Information for:

Cytokine-receptor targeting chimeras for targeted degradation of extracellular proteins

Authors & Affiliations:

Katarina Pance[1,2], Josef A. Gramespacher[1,2], James R. Byrnes[1], Fernando Salangsang[3,4], Juan-Antonio C. Serrano[3,4], Adam D. Cotton[1], Veronica Steri[3,4], James A. Wells[1,5*]

[1] *Department of Pharmaceutical Chemistry, University of California San Francisco, San Francisco, California, 94158, USA.*

[2] *Current Affiliation: EpiBiologics, Inc., San Carlos, California, 94070, USA.*

[3] *Helen Diller Family Comprehensive Cancer Center, University of California San Francisco, San Francisco, California, 94158, USA.*

[4] *Preclinical Therapeutics Core, University of California San Francisco, San Francisco, California, 94158, USA.*

[5] *Department of Cellular and Molecular Pharmacology, University of California San Francisco, San Francisco, California, 94158, USA.*

[*] *Corresponding Author: jim.wells@ucsf.edu*

Supplementary Data Table 1: Summary of CXCR7:target ratios and maximal degradation (D_{max}) for each cell surface KineTAC target.

Target Protein	Cell Line	CXCR7:Target mRNA Ratio[1]	D_{max} (%)
PD-L1	MDA-MB-231	0.5	73
HER2	MCF7	8.6	51
HER2	MDA-MB-175VII	1.3	62
HER2	SK-BR-3	0.03	26
EGFR	HeLa	6.0	84
EGFR	A431	0.4	54
EGFR	MDA-MB-231	0.2	66
EGFR	NCI-H292	0.1	74
EGFR	A549	0.2	72
EGFR	NCI-H358	0.04	79
EGFR	HCC827	0.0004	N/A
PD-1	Primary CD8+ T cells	N/A	82
CDCP1	HeLa	5.6	94
TROP2	MCF7	1.8	51

[1] mRNA levels are from the Cancer Cell Line Encyclopedia RNA-seq of 934 cancer cell lines and Genentech's RNA-seq of 675 commonly used human cancer cells.

Supplementary Data Table 2: ***In vitro*** **binding affinities of atezolizumab alanine mutants to PD-L1.**

Mutant	K_D (nM)	k_{on} ($M^{-1}s^{-1}$)	k_{off} (s^{-1})
WT	0.33	1.87×10^5	6.24×10^{-5}
S57A HC	0.52	2.53×10^5	1.31×10^{-4}
D31A HC	4.93	1.10×10^5	5.44×10^{-4}
S30A HC	1.40	4.36×10^4	6.09×10^{-5}
W33A HC	N.D.	N.D.	N.D.
W50A HC	63.1	1.38×10^5	8.69×10^{-3}
W101A HC	458	1.10×10^4	5.02×10^{-3}
S30A LC	1.23	8.48×10^4	1.05×10^{-4}
Y93A LC	5.12	2.16×10^5	1.10×10^{-3}
L92A LC	2.73	2.88×10^5	7.86×10^{-4}

Representative Flow Cytometry Gating Strategy: VEGF uptake in HeLa cells

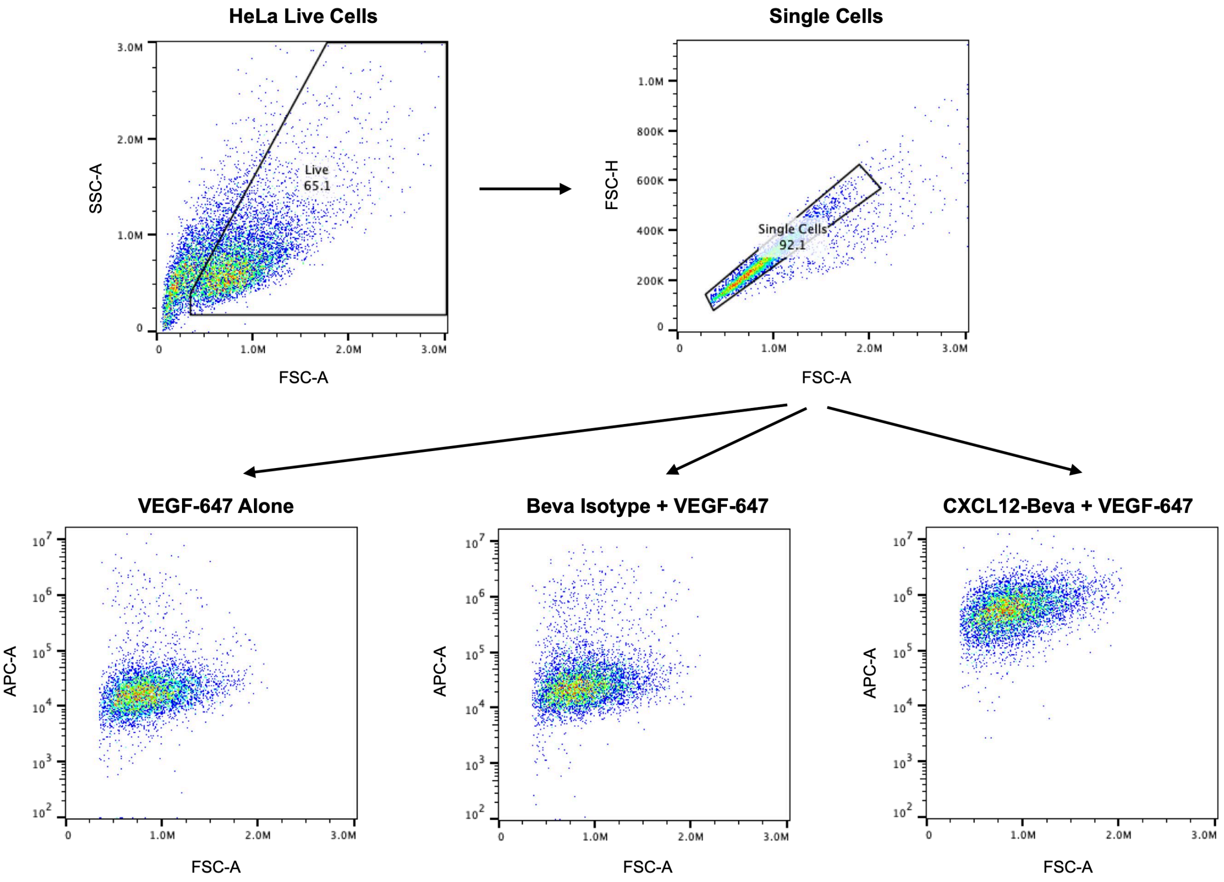

Antibodies and Detection Reagents

Antibody	Source (Cat. #)	Usage, Dilution
Rabbit anti-PD-L1	Cell Signaling Technologies (13684S)	WB, 1:1000
Rabbit anti-PD-L1 (mouse reactive)	Cell Signaling Technologies (60475S)	WB, 1:1000
Rabbit anti-EGFR	Cell Signaling Technologies (4267S)	WB, 1:1000
Rabbit anti-HER2	Cell Signaling Technologies (4290S)	WB, 1:1000
Rabbit anti-CDCP1	Cell Signaling Technologies (13794S)	WB, 1:1000
Rabbit anti-TROP2	Cell Signaling Technologies (E8Y8S)	WB, 1:1000
Rabbit anti-PD-1	Cell Signaling Technologies (86163S)	WB, 1:1000
Mouse anti-Tubulin	Cell Signaling Technologies (3873S)	WB, 1:1600

Mouse anti-β-Actin	Cell Signaling Technologies (3700S)	WB, 1:1000
Rabbit anti-CXCR7	Abcam (ab138509)	WB, 1:1000
Rabbit anti-CXCR4	Abcam (ab124824)	WB, 1:1000
680RD Goat anti-Mouse IgG	LI-COR (926-68070)	WB, 1:10000
800CW Goat anti-Rabbit IgG	LI-COR (926-32211)	WB, 1:10000
800CW Goat anti-Human IgG	LI-COR (926-32232)	WB, 1:10000
HRP anti-Rabbit IgG	Cell Signaling Technologies (7074A)	WB, 1:2000
Rabbit anti-PD-L1 Alexa 488 Conjugate	Cell Signaling Technologies (25048S)	IF, 1:50
Rabbit anti-EGFR Alexa 488 Conjugate	Cell Signaling Technologies (5616S)	IF, 1:100
Rabbit anti-EGFR Alexa 647 Conjugate	Cell Signaling Technologies (5588S)	IF, 1:50
Rabbit anti-LAMP1 Alexa 647 Conjugate	Cell Signaling Technologies (73589S)	IF, 1:50
Rabbit anti-Rab7 Alexa 647 Conjugate	Cell Signaling Technologies (94298S)	IF, 1:50
Rabbit anti-GM130 Alexa 647 Conjugate	Cell Signaling Technologies (59890S)	IF, 1:50
Rabbit anti-PD-L1 Alexa 647 Conjugate	Cell Signaling Technologies (41726S)	FC, 1:50; IF, 1:400
Anti-CD25 APC Conjugate	BioLegend (302610)	FC, 1:25
Anti-PD-1 PE Conjugate	BioLegend (329906)	FC, 1:25
Anti-CD3 PerCP/Cy5.5 Conjugate	BioLegend (300429)	FC, 1:25
Anti-CD8 APC/Fire 750 Conjugate	BioLegend (301066)	FC, 1:25
Rabbit IgG Isotype Control Alexa 647 Conjugate	Cell Signaling Technologies (3452S)	FC, 1:50
Mouse IgG2a Isotype Control PE Conjugate	BioLegend (400211)	FC, 1:25
Mouse IgG1 Isotype Control APC Conjugate	BioLegend (400119)	FC, 1:25
Cetuximab	Selleck Chemicals (A2000)	Functional
Biotinylated Human TNF-alpha	Acro Biosystems (TNA-H82E3)	Functional
Biotinylated Human VEGF-165	Acro Biosystems (VE5-H82Q0)	Functional
Streptavidin Alexa 647 Conjugate	Thermo Fisher Scientific (S21374)	FC, 1:500; IF, 1:500
Streptavidin Alexa 488 Conjugate	Thermo Fisher Scientific (S11223)	FC, 1:500; IF, 1:500

Human IL-2	Gold Biotechnologies (1110-02-1000)	Functional
Human IL-15	Gold Biotechnologies (1110-15-10)	Functional
Mouse IFN-gamma	R&D Systems (485-MI-100/CF)	Functional
Immunocult Human CD3/CD28 T cell Activator	STEMCELL Technologies (10971)	Functional
