# Supplementary material for: Modular cytokine receptor-targeting chimeras for targeted degradation of cell surface and extracellular proteins
Source: Nat Biotechnol. 2022 Sep 22;41(2):273–81. doi: 10.1038/s41587-022-01456-2 (PMC9931583; doi:10.1038/s41587-022-01456-2)
Supplement: Supplementary file 2 — Reporting Summary [file 41587_2022_1456_MOESM2_ESM.pdf]

nature portfolio

Corresponding author(s): James A. Wells

Last updated by author(s): Jul 21, 2022

Reporting Summary

Nature Portfolio wishes to improve the reproducibility of the work that we publish. This form provides structure for consistency and transparency in reporting. For further information on Nature Portfolio policies, see our Editorial Policies and the Editorial Policy Checklist.

Statistics

For all statistical analyses, confirm that the following items are present in the figure legend, table legend, main text, or Methods section.

n/a	Confirmed	
☐	☒	The exact sample size (*n*) for each experimental group/condition, given as a discrete number and unit of measurement
☐	☒	A statement on whether measurements were taken from distinct samples or whether the same sample was measured repeatedly
☐	☒	The statistical test(s) used AND whether they are one- or two-sided<br>*Only common tests should be described solely by name; describe more complex techniques in the Methods section.*
☒	☐	A description of all covariates tested
☒	☐	A description of any assumptions or corrections, such as tests of normality and adjustment for multiple comparisons
☐	☒	A full description of the statistical parameters including central tendency (e.g. means) or other basic estimates (e.g. regression coefficient) AND variation (e.g. standard deviation) or associated estimates of uncertainty (e.g. confidence intervals)
☐	☒	For null hypothesis testing, the test statistic (e.g. *F*, *t*, *r*) with confidence intervals, effect sizes, degrees of freedom and *P* value noted<br>*Give P values as exact values whenever suitable.*
☒	☐	For Bayesian analysis, information on the choice of priors and Markov chain Monte Carlo settings
☒	☐	For hierarchical and complex designs, identification of the appropriate level for tests and full reporting of outcomes
☒	☐	Estimates of effect sizes (e.g. Cohen's *d*, Pearson's *r*), indicating how they were calculated

Our web collection on statistics for biologists contains articles on many of the points above.

Software and code

Policy information about availability of computer code

Data collection	CytExpert (v 2.3.1.22) was used for flow cytometry acquisition. ForteBio Octet Data Acquisition (v 12.0.2.11) was used for biolayer interferometry acquisition. Image Studio Software (v 5.2) and Image Lab (v 5.0) was used for western blot scanning. Bruker Compass HyStar (v 5.1) was used for mass spectrometry data acquisition. NIS Elements (v 5.21.03) was used for fluorescence microscopy image acquisition).
Data analysis	FlowJo (v 10.8.0) was used for flow cytometry data analysis. ForteBio Data Analysis (v 12.0) was used for biolayer interferometry data analysis. Image Studio Software (v 5.2.5) was used for western blot quantification. PEAKS Online (v 1.5) was used for mass spectrometry data analysis. ImageJ (v 2.1.0) was used for fluorescence microscopy image analysis. GraphPad Prism (v 9.2.0) was used for data analysis.

For manuscripts utilizing custom algorithms or software that are central to the research but not yet described in published literature, software must be made available to editors and reviewers. We strongly encourage code deposition in a community repository (e.g. GitHub). See the Nature Portfolio guidelines for submitting code & software for further information.

Data

Policy information about availability of data

All manuscripts must include a data availability statement. This statement should provide the following information, where applicable:

- Accession codes, unique identifiers, or web links for publicly available datasets
- A description of any restrictions on data availability
- For clinical datasets or third party data, please ensure that the statement adheres to our policy

Quantitative proteomics data for PD-L1 and EGFR degradation experiments are provided in Supplementary Tables 3 and 4, respectively. Source data and raw gels are provided with the paper. All other raw data supporting the results are available upon reasonable request. Correspondence and requests for materials should be addressed to jim.wells@ucsf.edu.

Field-specific reporting

Please select the one below that is the best fit for your research. If you are not sure, read the appropriate sections before making your selection.

☒ Life sciences ☐ Behavioural & social sciences ☐ Ecological, evolutionary & environmental sciences

For a reference copy of the document with all sections, see nature.com/documents/nr-reporting-summary-flat.pdf

Life sciences study design

All studies must disclose on these points even when the disclosure is negative.

Sample size	For mouse experiments, calculation of sample size was performed by power analysis: experiment has been designed aiming at 80% power, using alpha = 0.5, to find effects that are significant at the 95% confidence level. We estimated a standard deviation of 20% due to technical noise and effect size from 20% to 35% (https://doi.org/10.1093/ilar.43.4.244). For all other experiments, sample size was chosen based on previous lab experience with the variability of different assays (i.e. western blotting, flow cytometry) and the ability to detect meaningful changes. In our experience, there is low variability between western blot and flow cytometry experiments. A minimum of N=2 replicates is therefore needed for all experiments.
Data exclusions	There were no data exclusions from this study.
Replication	All experiments were successfully replicated with at least two biological replicates.
Randomization	Initial choice of mice for injection of different concentrations of bispecific was random. No other formal randomization was employed. Randomization was not relevant to other experiments as this is an observational study which would not be affected by the relevant bias.
Blinding	As per UCSF PTC standard operating procedure, animal technicians performing in vivo procedures, drug dosing, bodyweight and tumor volume measurements are not aware of the experimental design of the study. Experimental cohorts are simply identified by numbers.<br><br>For non-mouse studies, both the samples and the controls were measured in the same sample plates under the same conditions. Data was first processed in a blinded mode, followed by sample/control assignments.

Reporting for specific materials, systems and methods

We require information from authors about some types of materials, experimental systems and methods used in many studies. Here, indicate whether each material, system or method listed is relevant to your study. If you are not sure if a list item applies to your research, read the appropriate section before selecting a response.

Materials & experimental systems

n/a	Involved in the study	
☐	☒	Antibodies
☐	☒	Eukaryotic cell lines
☒	☐	Palaeontology and archaeology
☐	☒	Animals and other organisms
☒	☐	Human research participants
☒	☐	Clinical data
☒	☐	Dual use research of concern

Methods

n/a	Involved in the study	
☒	☐	ChIP-seq
☐	☒	Flow cytometry
☒	☐	MRI-based neuroimaging

Antibodies

Antibodies used

Rabbit anti-PD-L1 Cell Signaling Technologies (13684S) WB, 1:1000
Rabbit anti-PD-L1 (mouse reactive) Cell Signaling Technologies (60475S) WB, 1:1000
Rabbit anti-EGFR Cell Signaling Technologies (4267S) WB, 1:1000
Rabbit anti-HER2 Cell Signaling Technologies (4290S) WB, 1:1000
Rabbit anti-CDCP1 Cell Signaling Technologies (13794S) WB, 1:1000
Rabbit anti-TROP2 Cell Signaling Technologies (E8Y8S) WB, 1:1000
Rabbit anti-PD-1 Cell Signaling Technologies (86163S) WB, 1:1000
Mouse anti-Tubulin Cell Signaling Technologies (3873S) WB, 1:1600
Mouse anti-?-Actin Cell Signaling Technologies (3700S) WB, 1:1000
Rabbit anti-CXCR7 Abcam (ab138509) WB, 1:1000
Rabbit anti-CXCR4 Abcam (ab124824) WB, 1:1000
680RD Goat anti-Mouse IgG LI-COR (926-68070) WB, 1:10000
800CW Goat anti-Rabbit IgG LI-COR (926-32211) WB, 1:10000
800CW Goat anti-Human IgG LI-COR (926-32232) WB, 1:10000
HRP anti-Rabbit IgG Cell Signaling Technologies (7074A) WB, 1:2000

Rabbit anti-PD-L1 Alexa 488 Conjugate Cell Signaling Technologies (25048S) IF, 1:50
Rabbit anti-EGFR Alexa 488 Conjugate Cell Signaling Technologies (5616S) IF, 1:100
Rabbit anti-EGFR Alexa 647 Conjugate Cell Signaling Technologies (5588S) IF, 1:50
Rabbit anti-LAMP1 Alexa 647 Conjugate Cell Signaling Technologies (73589S) IF, 1:50
Rabbit anti-Rab7 Alexa 647 Conjugate Cell Signaling Technologies (94298S) IF, 1:50
Rabbit anti-GM130 Alexa 647 Conjugate Cell Signaling Technologies (59890S) IF, 1:50
Rabbit anti-PD-L1 Alexa 647 Conjugate Cell Signaling Technologies (41726S) FC, 1:50; IF, 1:400
Anti-CD25 APC Conjugate BioLegend (302610) FC, 1:25
Anti-PD-1 PE Conjugate BioLegend (329906) FC, 1:25
Anti-CD3 PerCP/Cy5.5 Conjugate BioLegend (300429) FC, 1:25
Anti-CD8 APC/Fire 750 Conjugate BioLegend (301066) FC, 1:25
Rabbit IgG Isotype Control Alexa 647 Conjugate Cell Signaling Technologies (3452S) FC, 1:50
Mouse IgG2a Isotype Control PE Conjugate BioLegend (400211) FC, 1:25
Mouse IgG1 Isotype Control APC Conjugate BioLegend (400119) FC, 1:25
Cetuximab Selleck Chemicals (A2000) Functional

Validation

All antibodies used were validated by antibody suppliers per quality assurance as detailed on each supplier's website.

Rabbit anti-PD-L1 (13684S, Cell Signaling Technologies): https://www.cellsignal.com/products/primary-antibodies/pd-l1-e1l3n-xp-rabbit-mab/13684. Validated from manufacturer's website and citations therein. Validation included upregulation of PD-L1 in A549 cells following treatment with IFN-gamma.
Rabbit anti-PD-L1 (mouse reactive) (60475S, Cell Signaling Technologies): https://www.cellsignal.com/products/primary-antibodies/pd-l1-d4h1z-rabbit-mab/60475. Validated from manufacturer's website and citations therein.
Rabbit anti-EGFR (4267S, Cell Signaling Technologies): https://www.cellsignal.com/products/primary-antibodies/egf-receptor-d38b1-xp-rabbit-mab/4267. Validated from manufacturer's website and citations therein. Validation included knockdown of EGFR in HeLa cells to show absence of EGFR.
Rabbit anti-HER2 (4290S, Cell Signaling Technologies): https://www.cellsignal.com/products/primary-antibodies/her2-erbb2-d8f12-xp-rabbit-mab/4290. Validated from manufacturer's website and citations therein. Validation included blotting for HER2 on known expressing cell lines, SK-BR-3 and MCF7.
Rabbit anti-CDCP1 (13794S, Cell Signaling Technologies): https://www.cellsignal.com/products/primary-antibodies/cdcp1-d1w9n-rabbit-mab/13794. Validated from manufacturer's website and citations therein. Validation included blotting for CDCP1 presence on ZR-75-30 (CDCP1+) and MCF7 (CDCP1-) cells.
Rabbit anti-TROP2 (E8Y8S, Cell Signaling Technologies): https://www.cellsignal.com/products/primary-antibodies/tacstd2-trop2-e8y8s-rabbit-mab/47866. Validated from manufacturer's website and citations therein.
Rabbit anti-PD-1 (86163S, Cell Signaling Technologies): https://www.cellsignal.com/products/primary-antibodies/pd-1-d4w2j-xp-rabbit-mab/86163. Validated from manufacturer's website and citations therein. Validation included blotting for PD-1 presence on MOLT-4 (PD-1+) and Jurkat (PD-1-) cells.
Mouse anti-Tubulin (3873S, Cell Signaling Technologies): https://www.cellsignal.com/products/primary-antibodies/a-tubulin-dm1a-mouse-mab/3873. Validated from manufacturer's website and citations therein.
Mouse anti-Beta-Actin (3700S, Cell Signaling Technologies): https://www.cellsignal.com/products/primary-antibodies/b-actin-8h10d10-mouse-mab/3700. Validated from manufacturer's website and citations therein.
Rabbit anti-CXCR7 (ab138509, Abcam): https://www.abcam.com/gpcr-rdc1cxcr-7-antibody-epr9321-ab138509.html. Validated from manufacturer's website and citations therein. Validation included blotting for CXCR7 presence on positive control cell lines THP-1, K562, Raji, C6, Raw 264.7, and NIH3T3 cell lysates.
Rabbit anti-CXCR4 (ab124824, Abcam): https://www.abcam.com/cxcr4-antibody-umb2-ab124824.html. Validated from manufacturer's website and citations therein. Validation included transfection of HEK293 cells with CXCR4.
680RD Goat anti-Mouse IgG (926-68070, LI-COR): https://www.licor.com/bio/reagents/irdye-680rd-goat-anti-mouse-igg-secondary-antibody. Validated from manufacturer's website and citations therein.
800CW Goat anti-Rabbit IgG (926-32211, LI-COR): https://www.licor.com/bio/reagents/irdye-800cw-goat-anti-rabbit-igg-secondary-antibody. Validated from manufacturer's website and citations therein.
800CW Goat anti-Human IgG (926-32232, LI-COR): https://www.licor.com/bio/reagents/irdye-800cw-goat-anti-human-igg-secondary-antibody. Validated from manufacturer's website and citations therein.
HRP anti-Rabbit IgG (7074A, Cell Signaling Technologies): https://www.cellsignal.com/products/secondary-antibodies/anti-rabbit-igg-hrp-linked-antibody/7074. Validated from manufacturer's website and citations therein.
Rabbit anti-PD-L1 Alexa 488 Conjugate (25048S, Cell Signaling Technologies): https://www.cellsignal.com/products/antibody-conjugates/pd-l1-extracellular-domain-specific-d8t4x-rabbit-mab-alexa-fluor-488-conjugate/25048. Validated from manufacturer's website and citations therein. Validation included presence of PD-L1 on positive control cell line MDA-MB-231 but not negative control cell line A549.
Rabbit anti-EGFR Alexa 488 Conjugate (5616S, Cell Signaling Technologies): https://www.cellsignal.com/products/antibody-conjugates/egf-receptor-d38b1-xp-rabbit-mab-alexa-fluor-488-conjugate/5616. Validated from manufacturer's website and citations therein. Validation included treatment of A549 cells with or without EGF.
Rabbit anti-EGFR Alexa 647 Conjugate (5588S, Cell Signaling Technologies): https://www.cellsignal.com/products/antibody-conjugates/egf-receptor-d38b1-xp-rabbit-mab-alexa-fluor-647-conjugate/5588. Validated from manufacturer's website and citations therein. Validation included treatment of A549 cells with or without EGF.
Rabbit anti-LAMP1 Alexa 647 Conjugate (73589S, Cell Signaling Technologies): https://www.cellsignal.com/products/antibody-conjugates/lamp1-d2d11-xp-rabbit-mab-alexa-fluor-647-conjugate/73589. Validated from manufacturer's website and citations therein.
Rabbit anti-Rab7 Alexa 647 Conjugate (94298S, Cell Signaling Technologies): https://www.cellsignal.com/products/antibody-conjugates/rab7-d95f2-xp-rabbit-mab-alexa-fluor-647-conjugate/94298. Validated from manufacturer's website and citations therein.
Rabbit anti-GM130 Alexa 647 Conjugate (59890S, Cell Signaling Technologies): https://www.cellsignal.com/products/antibody-conjugates/gm130-d6b1-xp-rabbit-mab-alexa-fluor-647-conjugate/59890. Validated from manufacturer's website and citations therein.
Rabbit anti-PD-L1 Alexa 647 Conjugate (41726S, Cell Signaling Technologies): https://www.cellsignal.com/products/antibody-conjugates/pd-l1-extracellular-domain-specific-d8t4x-rabbit-mab-alexa-fluor-647-conjugate/41726. Validated from manufacturer's website and citations therein.

Anti-CD25 APC Conjugate (302610, BioLegend): https://www.biolegend.com/en-us/products/apc-anti-human-cd25-antibody-614?GroupID=BLG1983. Validated from manufacturer's website and citations therein. Validation included upregulation of CD25 on the surface of PHA-stimulated human peripheral blood lymphocytes.
Anti-PD-1 PE Conjugate (329906, BioLegend): https://www.biolegend.com/en-us/products/pe-anti-human-cd279-pd-1-antibody-4412?GroupID=BLG5466. Validated from manufacturer's website and citations therein. Validation included upregulation of PD-1 on the surface of PHA-stimulated human peripheral blood lymphocytes.
Anti-CD3 PerCP/Cy5.5 Conjugate (300429, BioLegend): https://www.biolegend.com/en-us/products/percp-cyanine5-5-anti-human-cd3-antibody-4214?GroupID=BLG5900. Validated from manufacturer's website and citations therein.
Anti-CD8 APC/Fire 750 Conjugate (301066, BioLegend): https://www.biolegend.com/en-us/products/apc-fire-750-anti-human-cd8a-antibody-13580?GroupID=BLG5903. Validated from manufacturer's website and citations therein.
Rabbit IgG Isotype Control Alexa 647 Conjugate (3452S, Cell Signaling Technologies): https://www.cellsignal.com/products/antibody-conjugates/rabbit-igg-isotype-control-alexa-fluor-647-conjugate/3452. Validated from manufacturer's website and citations therein.
Mouse IgG2a Isotype Control PE Conjugate (400211, BioLegend): https://www.biolegend.com/en-us/search-results/pe-mouse-igg2a-kappa-isotype-ctrl-1401. Validated from manufacturer's website and citations therein.
Mouse IgG1 Isotype Control APC Conjugate (400119, BioLegend): https://www.biolegend.com/en-us/products/apc-mouse-igg1-kappa-isotype-ctrl-1404?GroupID=ImportedGROUP1. Validated from manufacturer's website and citations therein.
Cetuximab (A2000, Selleck Chemicals): https://www.selleckchem.com/products/cetuximab.html. Validated from manufacturer's website and citations therein.

Eukaryotic cell lines

Policy information about cell lines

Cell line source(s)
MDA-MB-231, HeLa, MDA-MB-175VII, MCF7, A431, A549, NCI-H292, NCI-H358, and SK-BR-3 cells were obtained from UCSF's Cell and Genome Engineering Core. Expi293 (Thermo Fisher) expressing and ER-BirA plasmid was used for protein expression only.

Authentication
Cell lines were authenticated by the supplier. Cell lines were validated by supplier for endotoxin levels using LAL gel clot assay and cell line authenticity validated by STR.

Mycoplasma contamination
The cell lines were not tested for mycoplasma contamination.

Commonly misidentified lines (See ICLAC register)
No commonly misidentified lines were used.

Animals and other organisms

Policy information about studies involving animals; ARRIVE guidelines recommended for reporting animal research

Laboratory animals
Male nude nu/nu mice, 8-10 weeks old, bred at the UCSF MZ Breeding Facility. Mice are housed in the UCSF Animal Care Facility LARC at the Helen Diller Family Cancer Center at UCSF Mission Bay. They are housed in an individual specific-pathogen free suite. They are housed up to 5 per cage in ventilator cages, with ad libitum food and water on a 12-hour light cycle and controlled temperature and humidity conditions (67-74F and 30-70%, respectively).

Wild animals
No wild animals were involved in this study.

Field-collected samples
There were no field collected samples involved in this study.

Ethics oversight
All animal care and experimentation were conducted in full accordance with UCSF Institutional Animal Care and Use Committee (IACUC) protocol AN179937.

Note that full information on the approval of the study protocol must also be provided in the manuscript.

Flow Cytometry

Plots

Confirm that:

☒ The axis labels state the marker and fluorochrome used (e.g. CD4-FITC).

☒ The axis scales are clearly visible. Include numbers along axes only for bottom left plot of group (a 'group' is an analysis of identical markers).

☒ All plots are contour plots with outliers or pseudocolor plots.

☒ A numerical value for number of cells or percentage (with statistics) is provided.

Methodology

Sample preparation
For surface staining experiments, cell pellets were washed with cold PBS and centrifuged at 300xg for 5 min. Cells were blocked with cold PBS + 3% BSA and centrifuged (300xg for 5 min). Cells were incubated with primary antibodies diluted in PBS + 3% BSA for 30 min at 4ºC. Cells were washed three times with cold PBS + 3% BSA and secondary antibodies (if applicable) diluted in PBS + 3% BSA added and incubated for 30 min at 4ºC. Cells were washed three times with cold PBS + 3% BSA and resuspended in cold PBS. Flow cytometry was performed on a CytoFLEX cytometer (Beckman Coulter) and gating

was performed on single cells and live cells before acquisition of 10,000 cells. Analysis was performed using the FlowJo software package. For soluble ligand uptake experiments, cell pellets were washed three times with cold PBS and centrifuged at 300xg for 5 min. Cells were then resuspended in cold PBS. Flow cytometry was performed on a CytoFLEX cytometer (Beckman Coulter) and gating was performed on single cells and live cells before acquisition of 10,000 cells. Analysis was performed using the FlowJo software package.

Instrument

Beckman Coulter CytoFLEX cytometer (model no. A00-1-1102)

Software

CytExpert (v 2.3.1.22) was used for data acquisition, FlowJo (v 10.8.0) was used for data analysis.

Cell population abundance

For live vs. dead, cell population abundance was determined by gating live cells using FSC and SSC area followed by doublet removal using FSC-H vs FSC-A. For CD8+ T cell isolation, cell population abundance was determined by gating on the CD8+ CD3+ population (as determined through the use of anti-CD3 and anti-CD8 primary antibodies).

Gating strategy

Gating was based on FSC and SSC area followed by doublet removal using FSC-H vs FSC-A.

☒ Tick this box to confirm that a figure exemplifying the gating strategy is provided in the Supplementary Information.
