# Supplementary figures and images for: Modular cytokine receptor-targeting chimeras for targeted degradation of cell surface and extracellular proteins
Source: Nat Biotechnol. 2022 Sep 22;41(2):273–81. doi: 10.1038/s41587-022-01456-2 (PMC9931583; doi:10.1038/s41587-022-01456-2)

Figure 1

d.

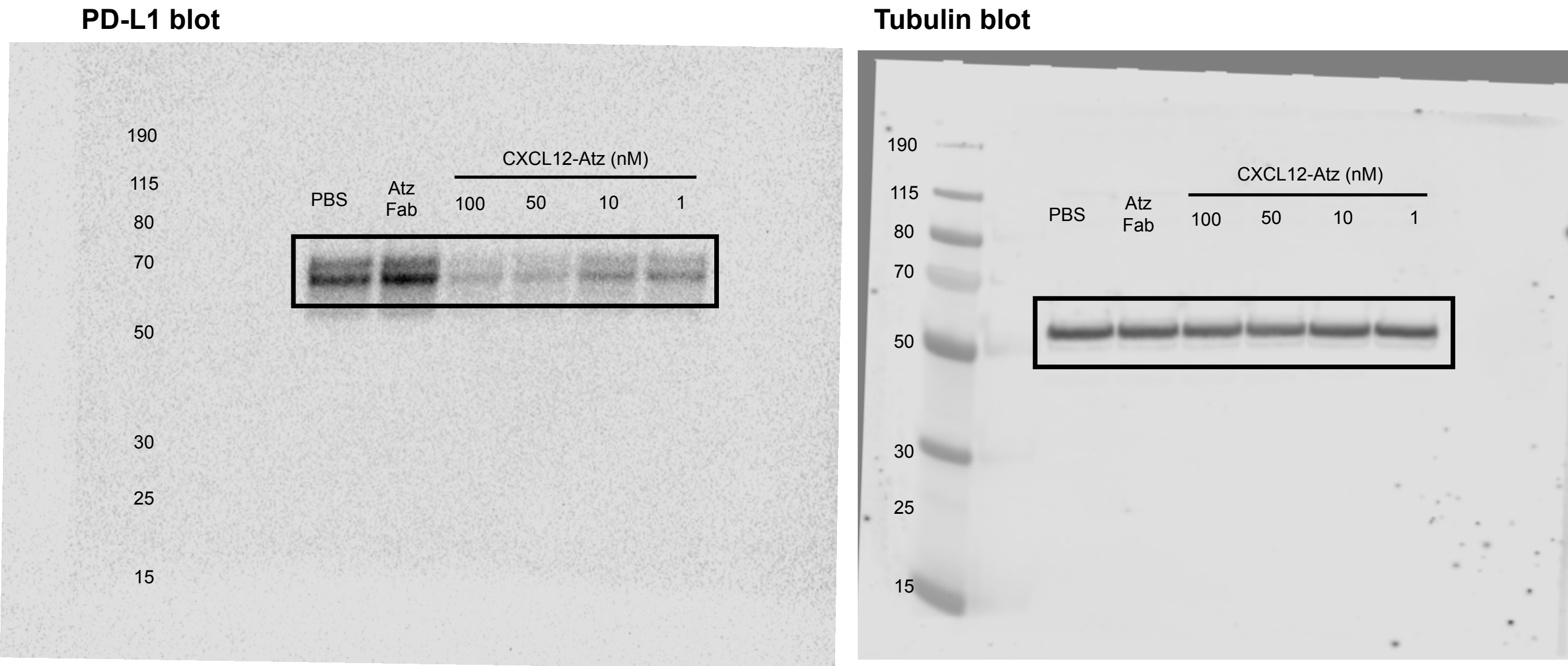

g.

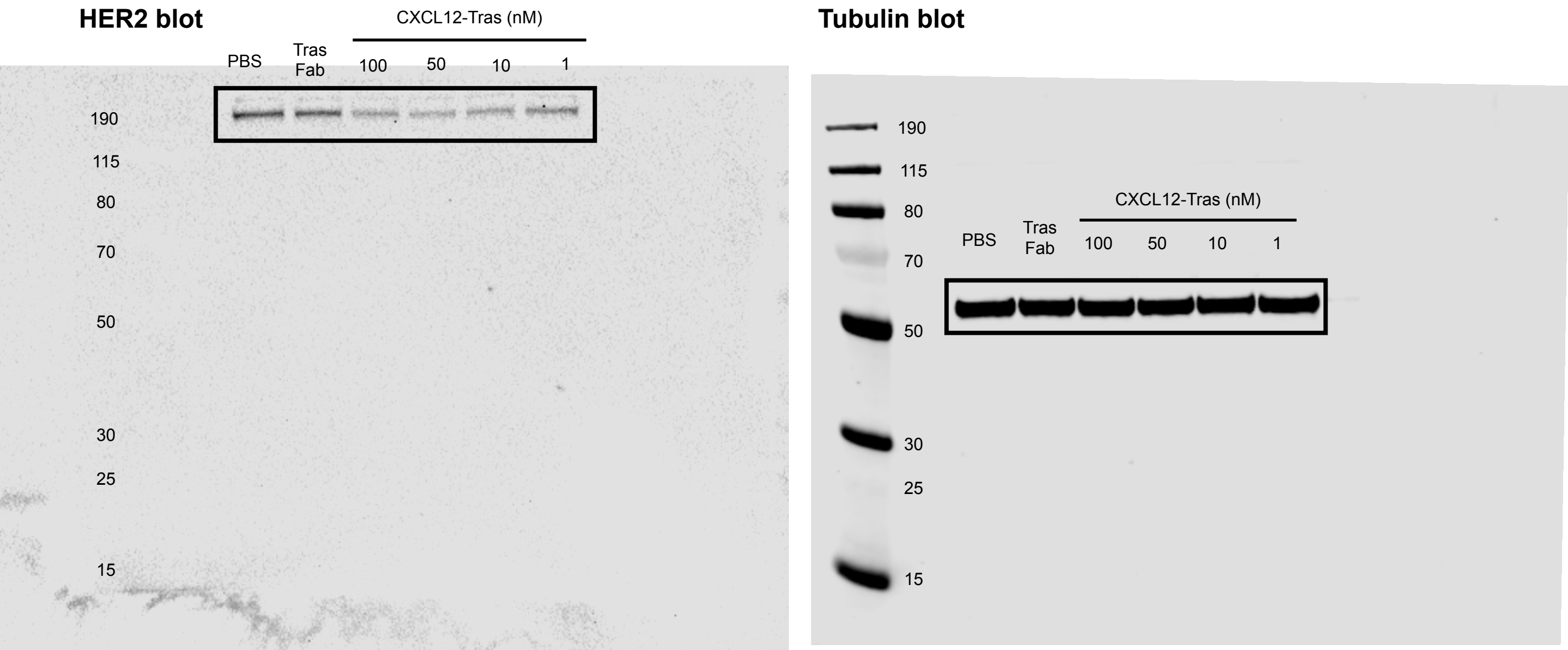

i.

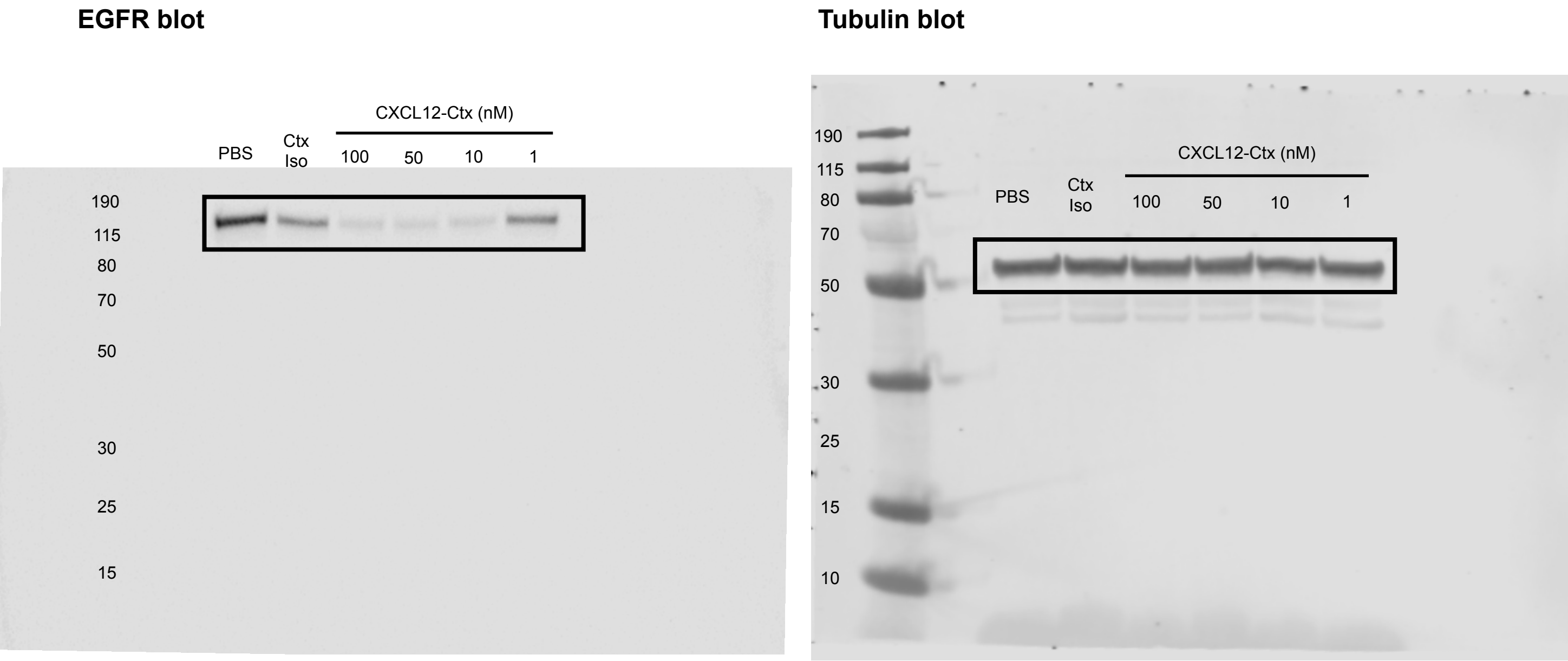

Supplement: Source Data Fig. 1 — Full-length, unprocessed gels or blots. [file 41587_2022_1456_MOESM6_ESM.pdf]

Figure 2

a.

PD-L1 blot

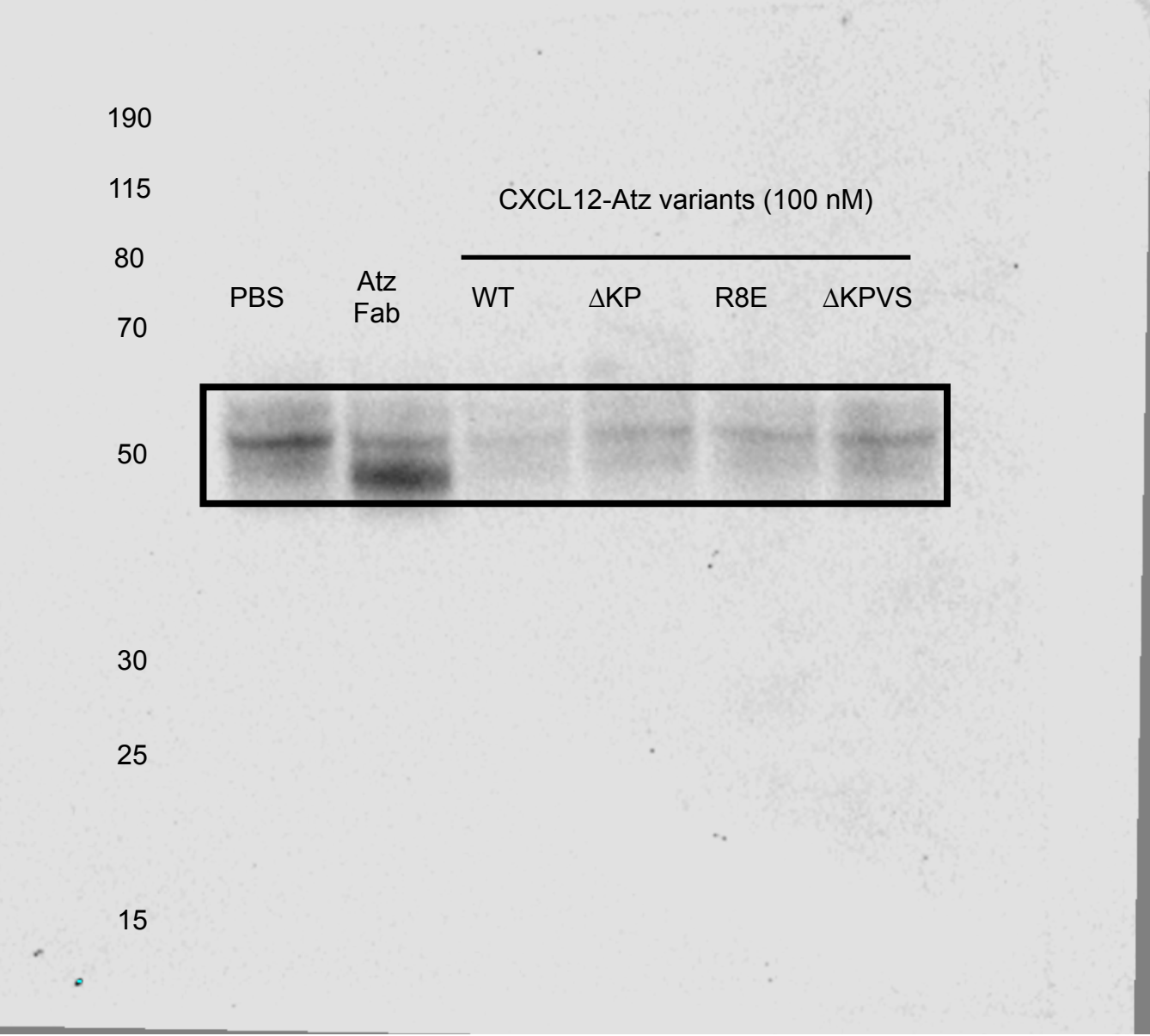

Tubulin blot

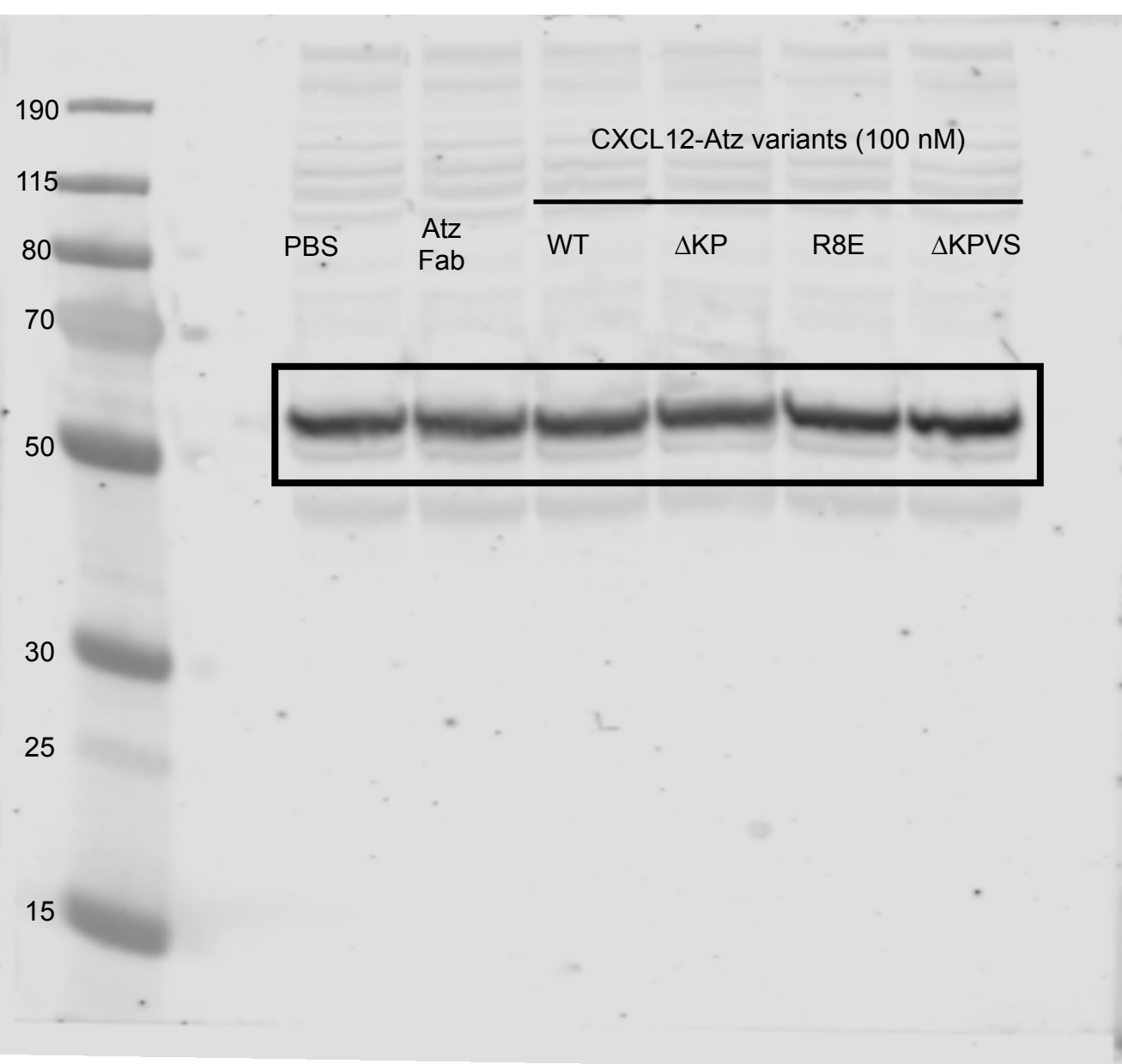

g.

PD-L1 blot

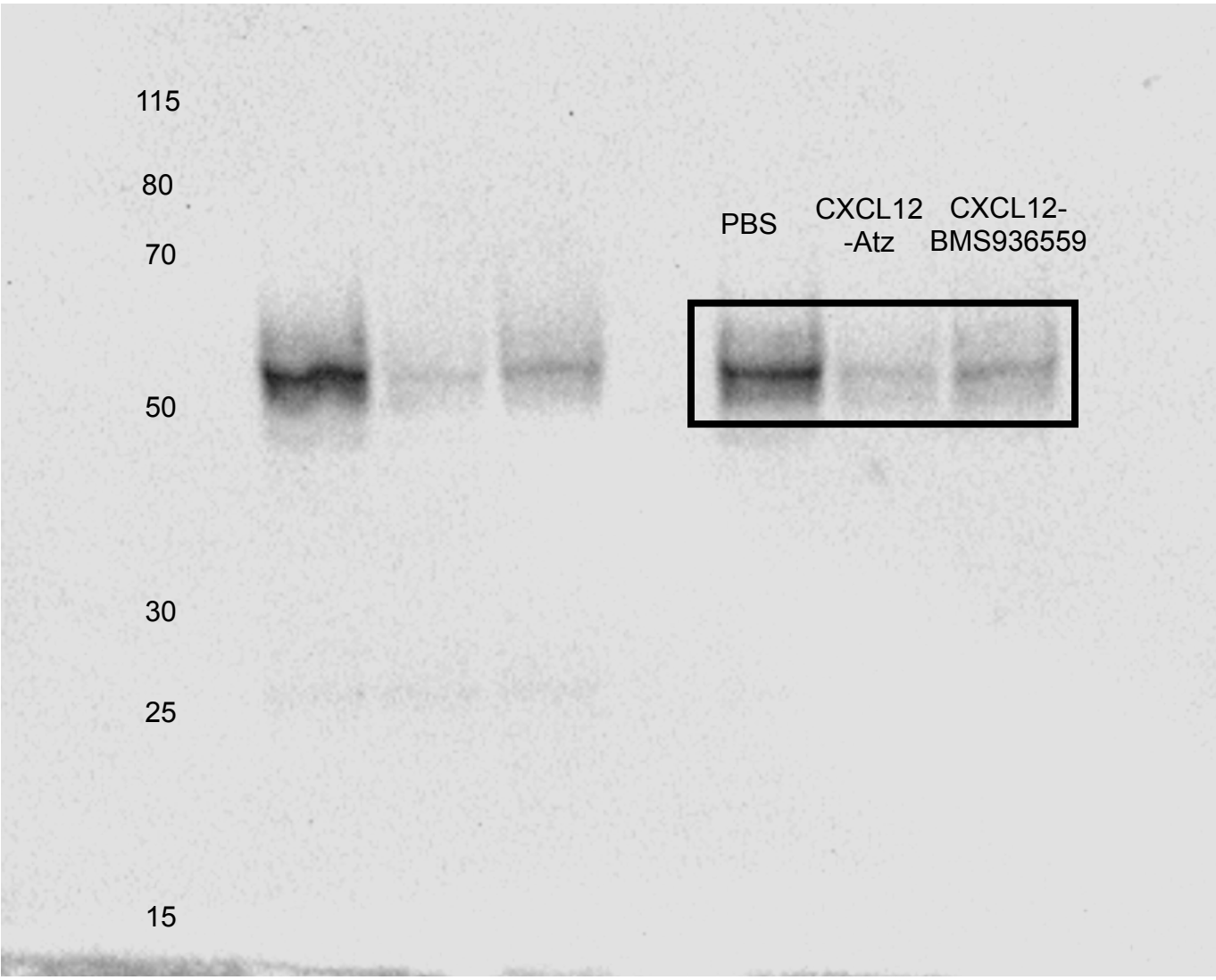

Tubulin blot

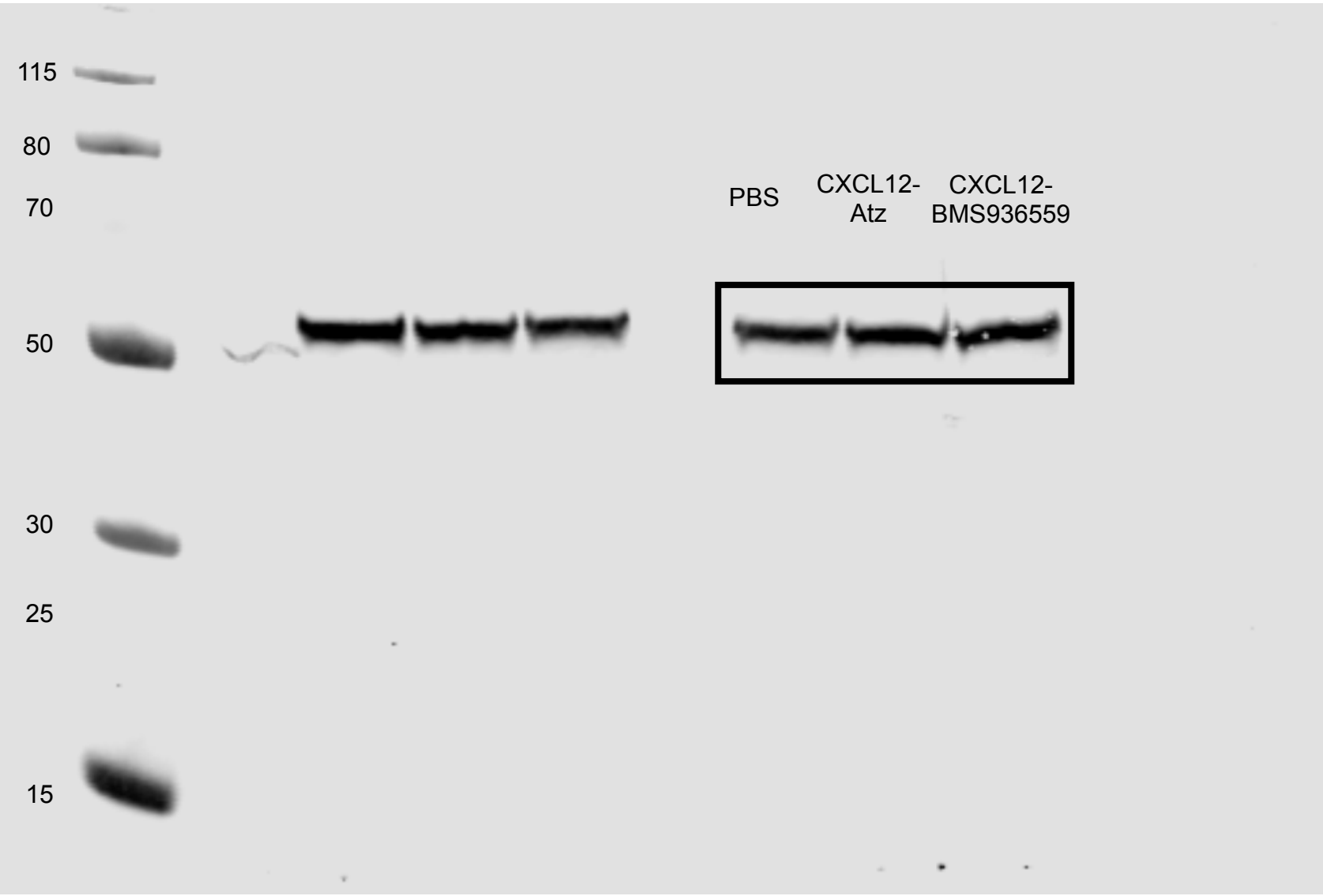

Supplement: Source Data Fig. 2 — Full-length, unprocessed gels or blots. [file 41587_2022_1456_MOESM8_ESM.pdf]

Figure 3

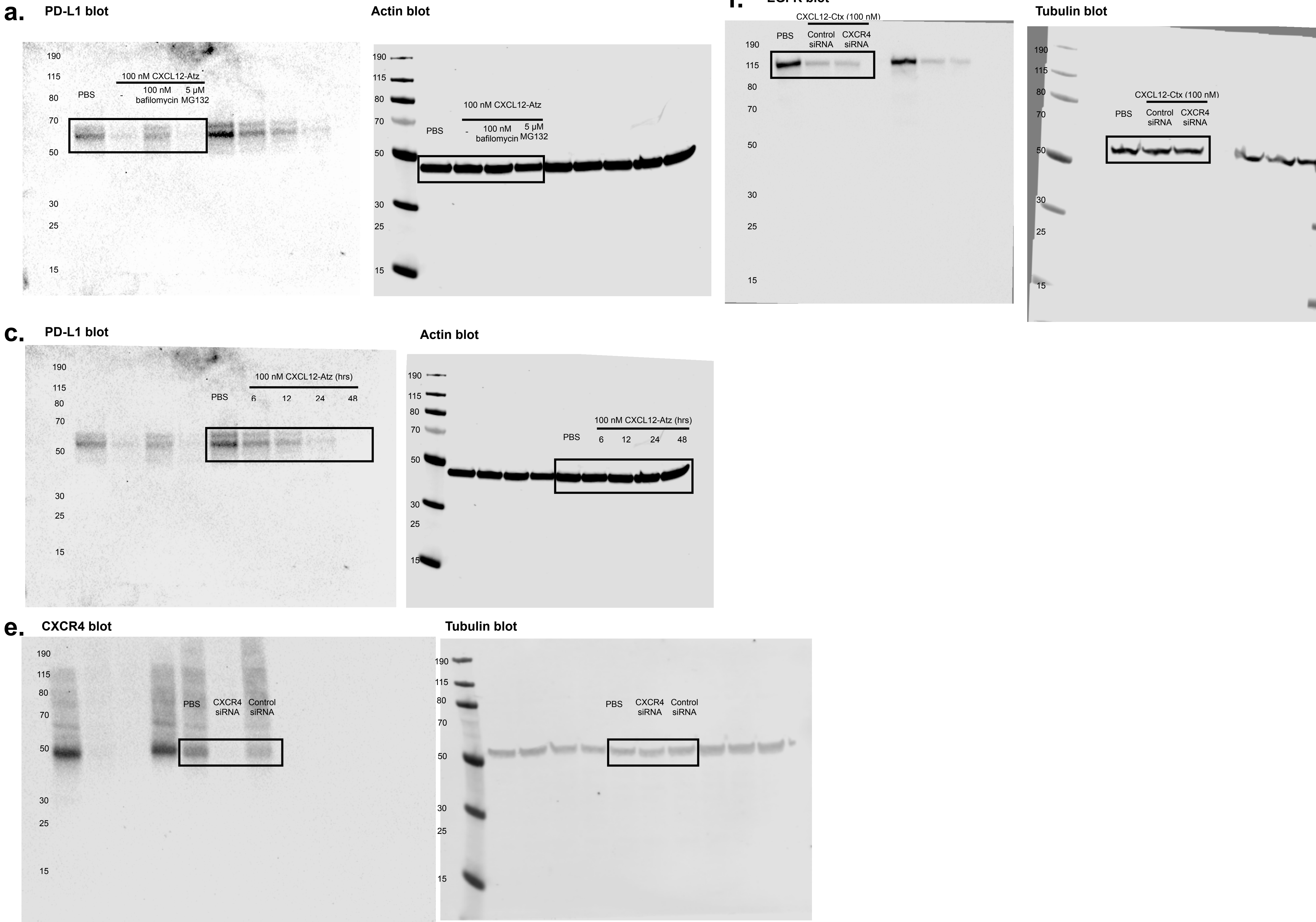

Supplement: Source Data Fig. 3 — Full-length, unprocessed gels or blots. [file 41587_2022_1456_MOESM10_ESM.pdf]

# Extended Data Figure 2

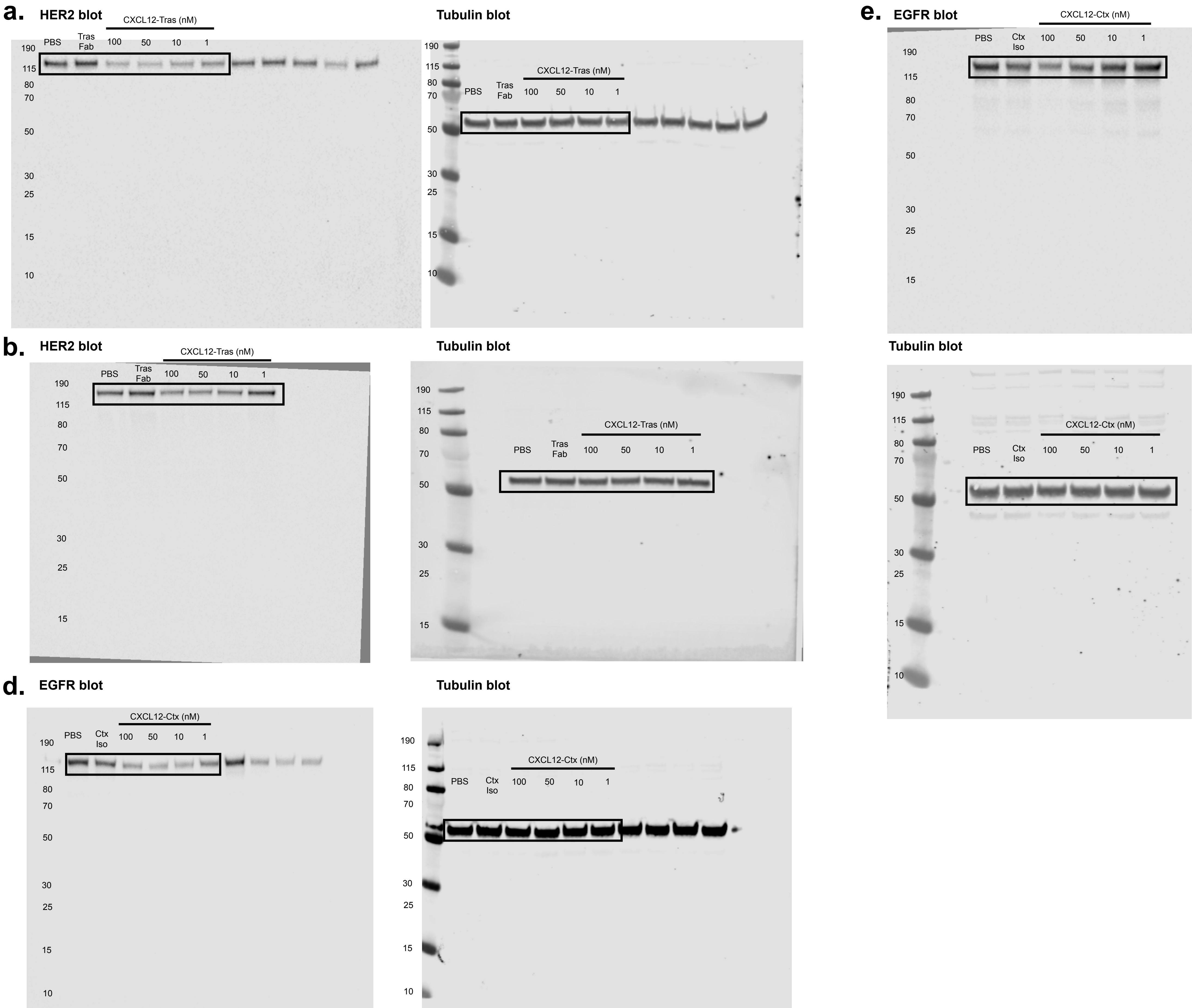

# Extended Data Figure 2

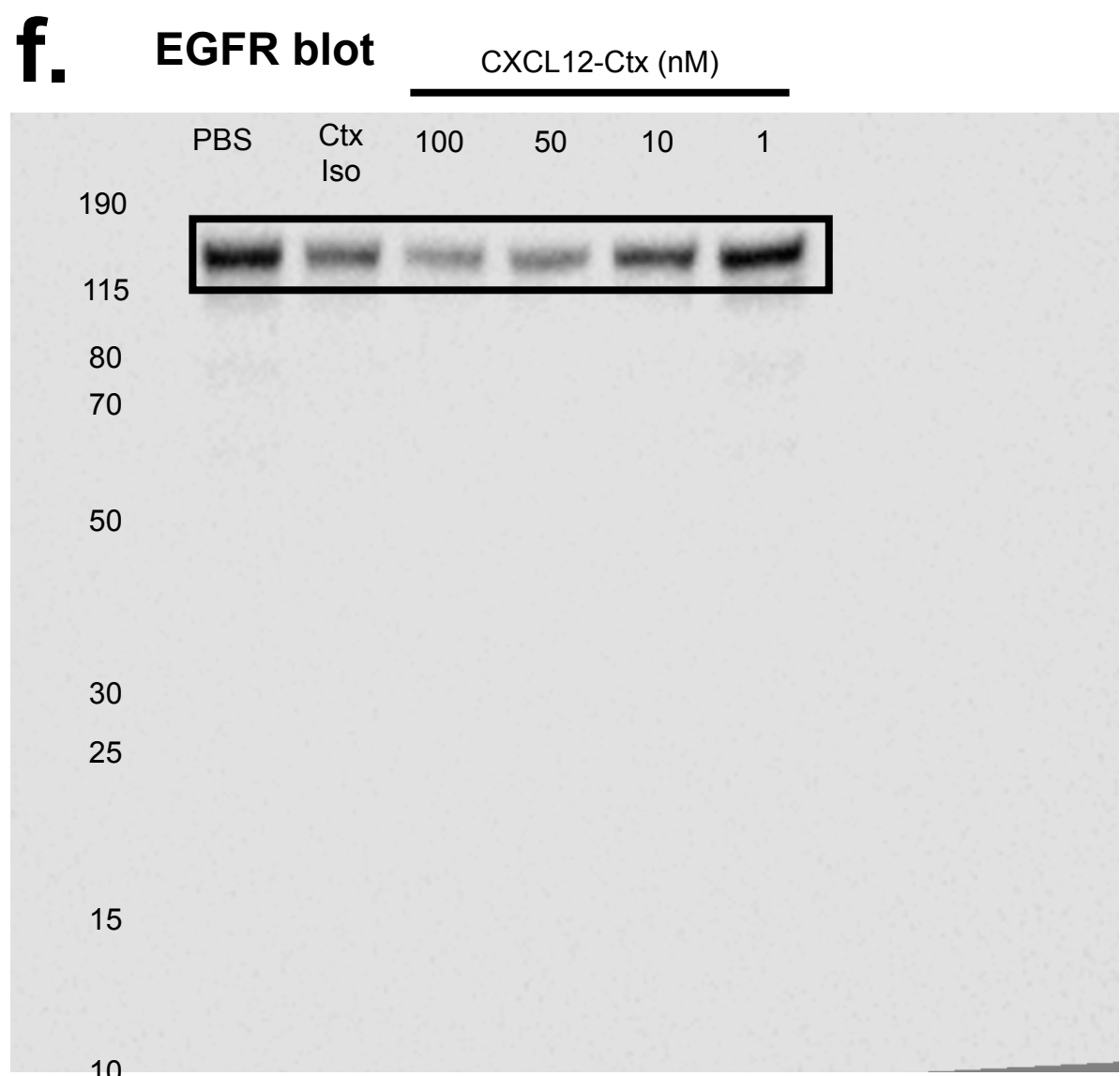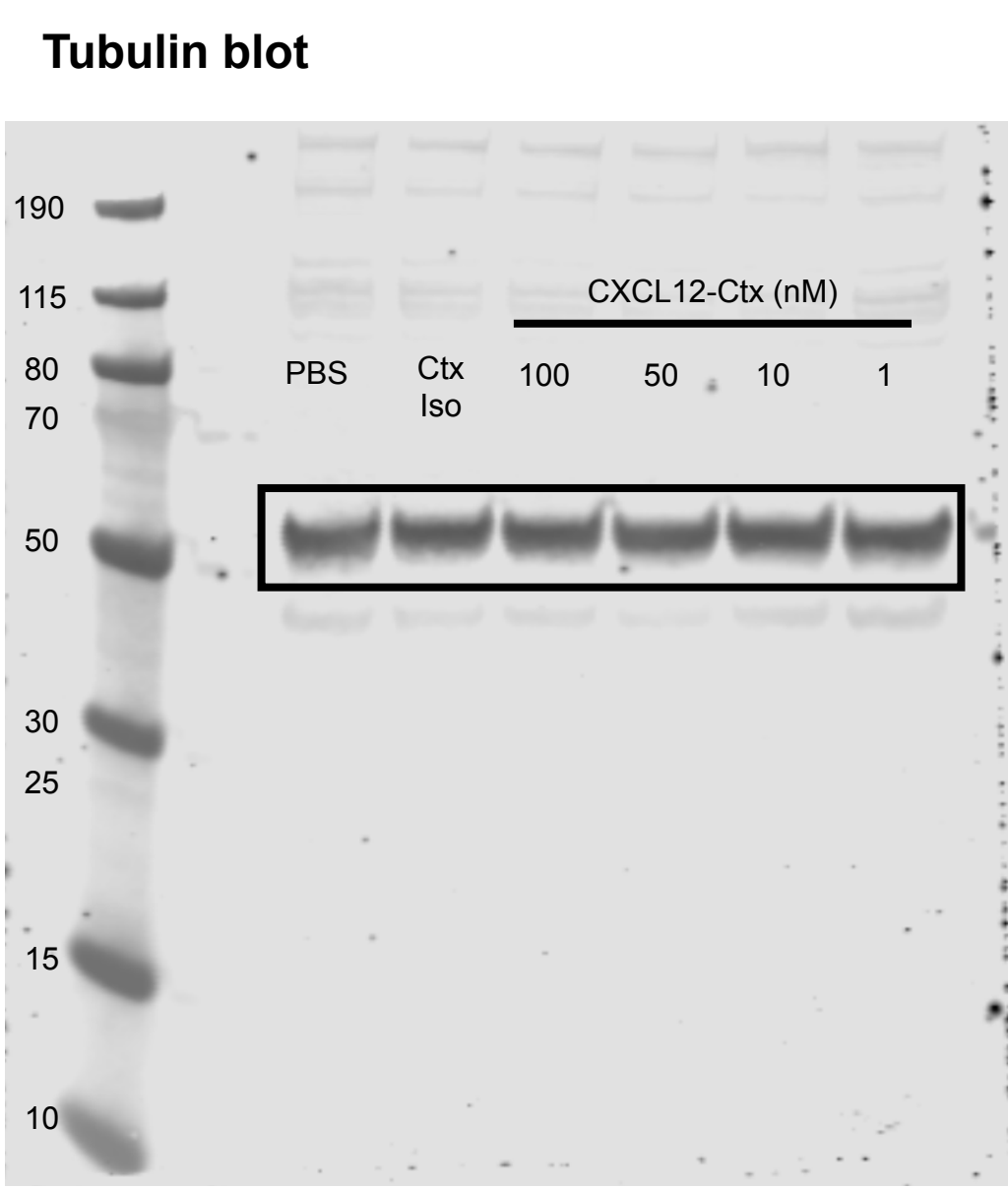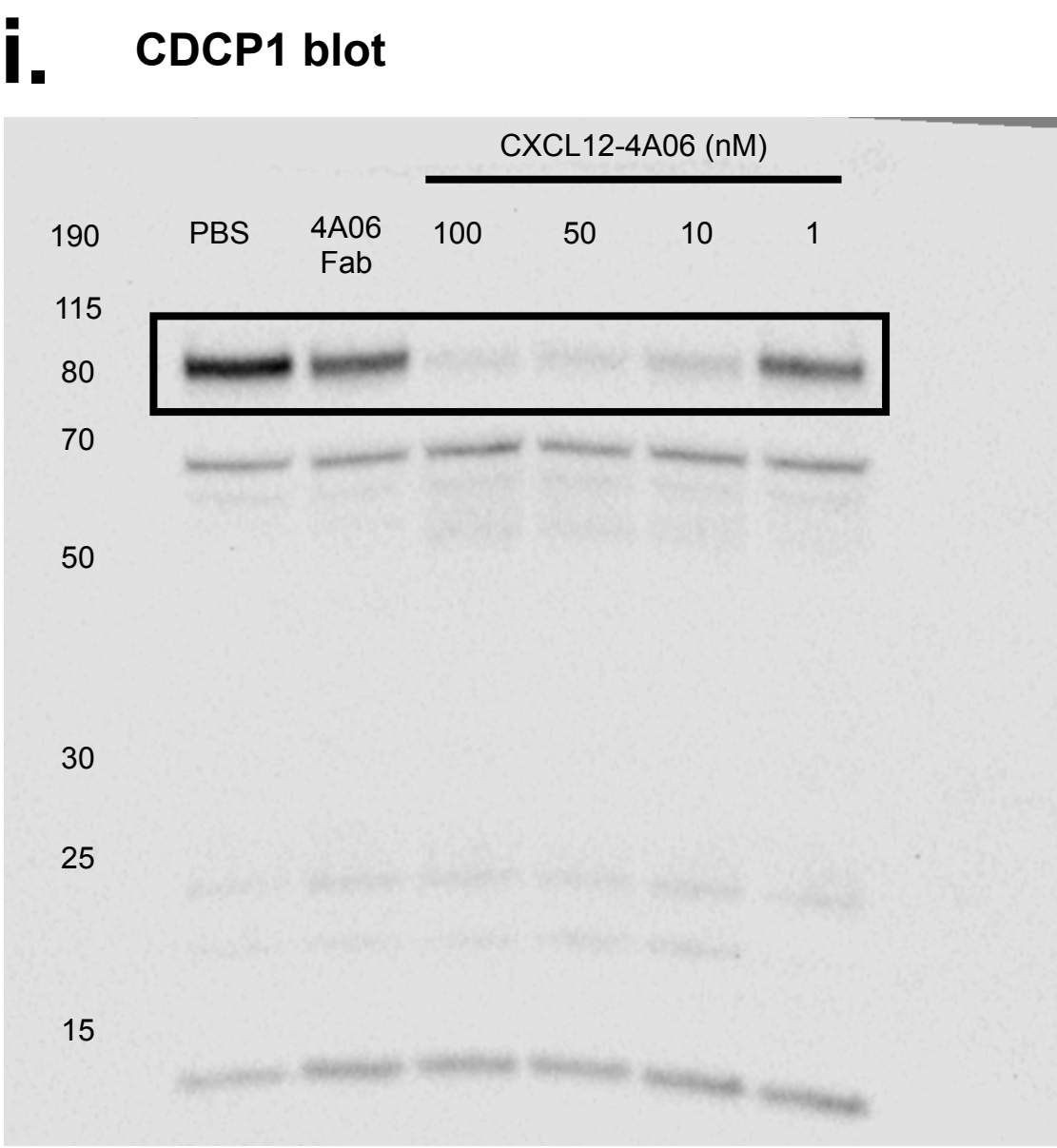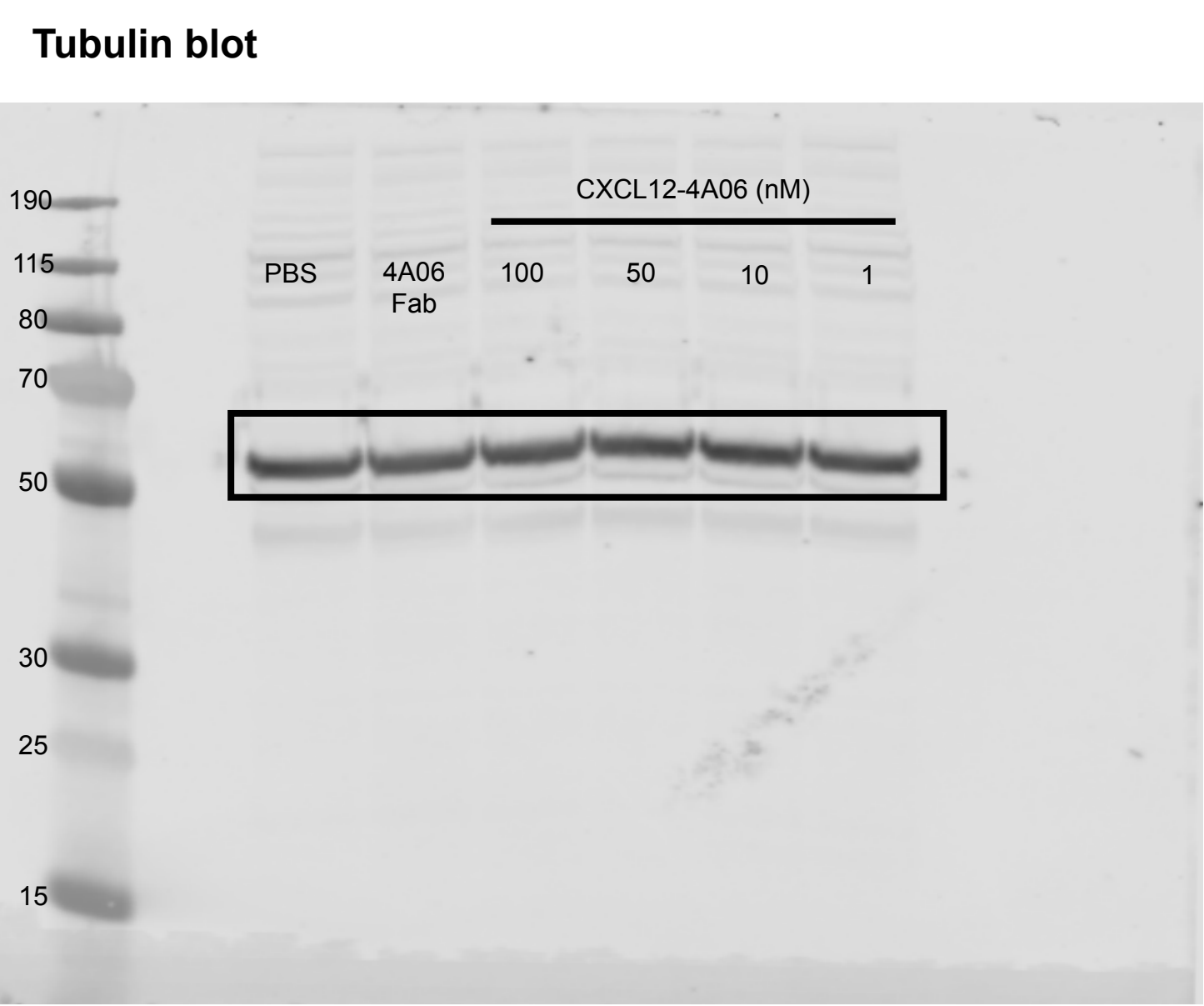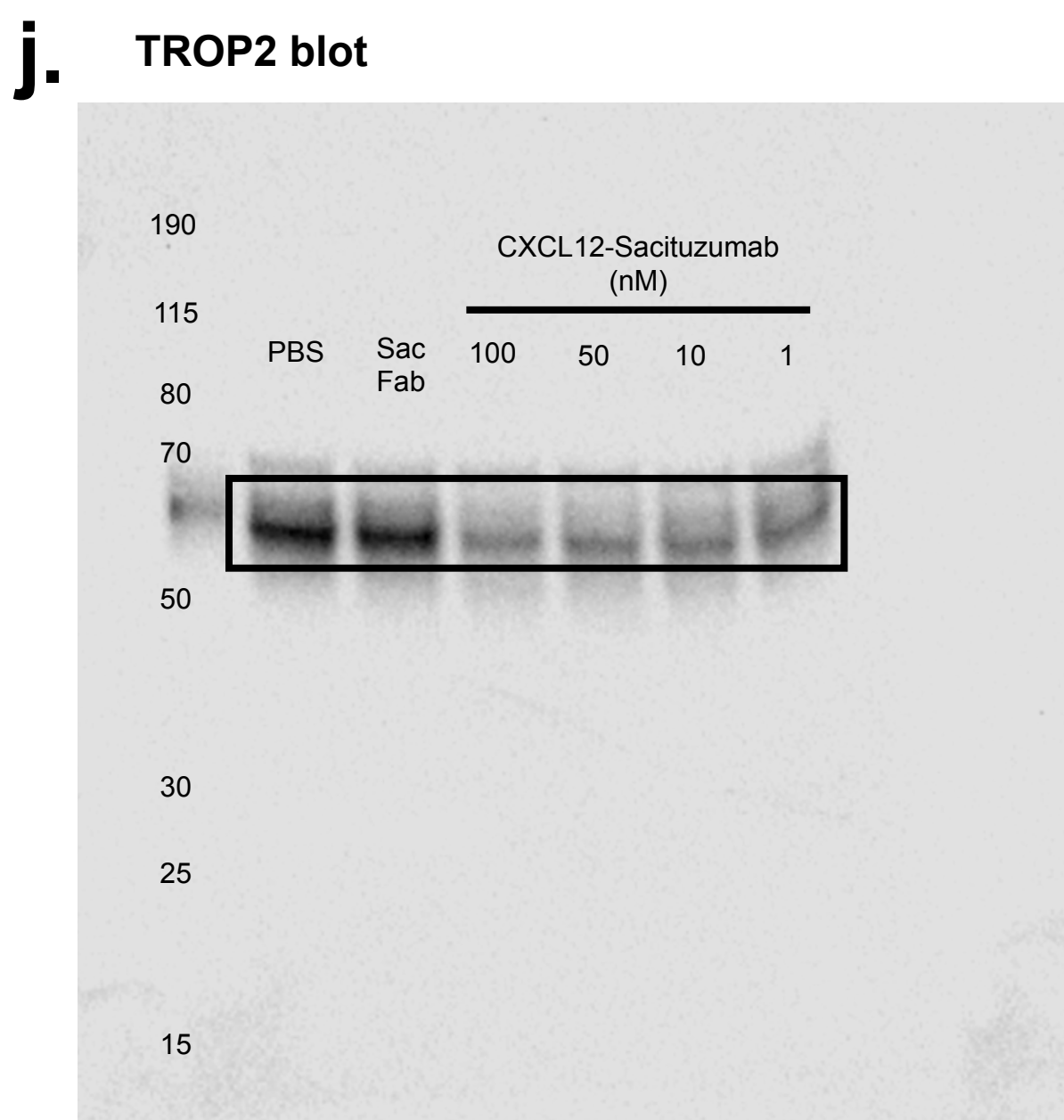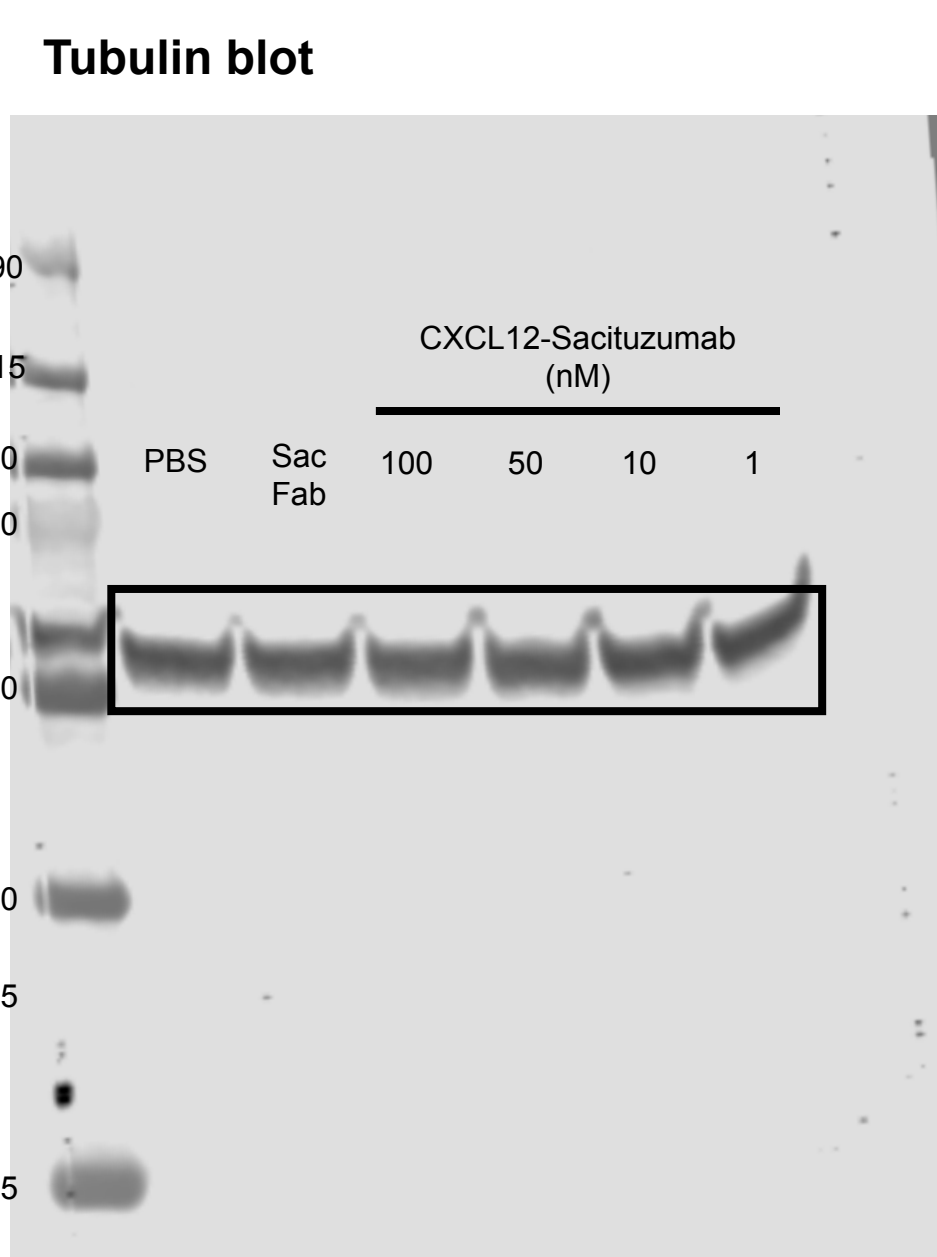

Supplement: Source Data Extended Data Fig. 2 — Full-length, unprocessed gels or blots. [file 41587_2022_1456_MOESM14_ESM.pdf]

Extended Data Figure 4

a. PD-L1 blot

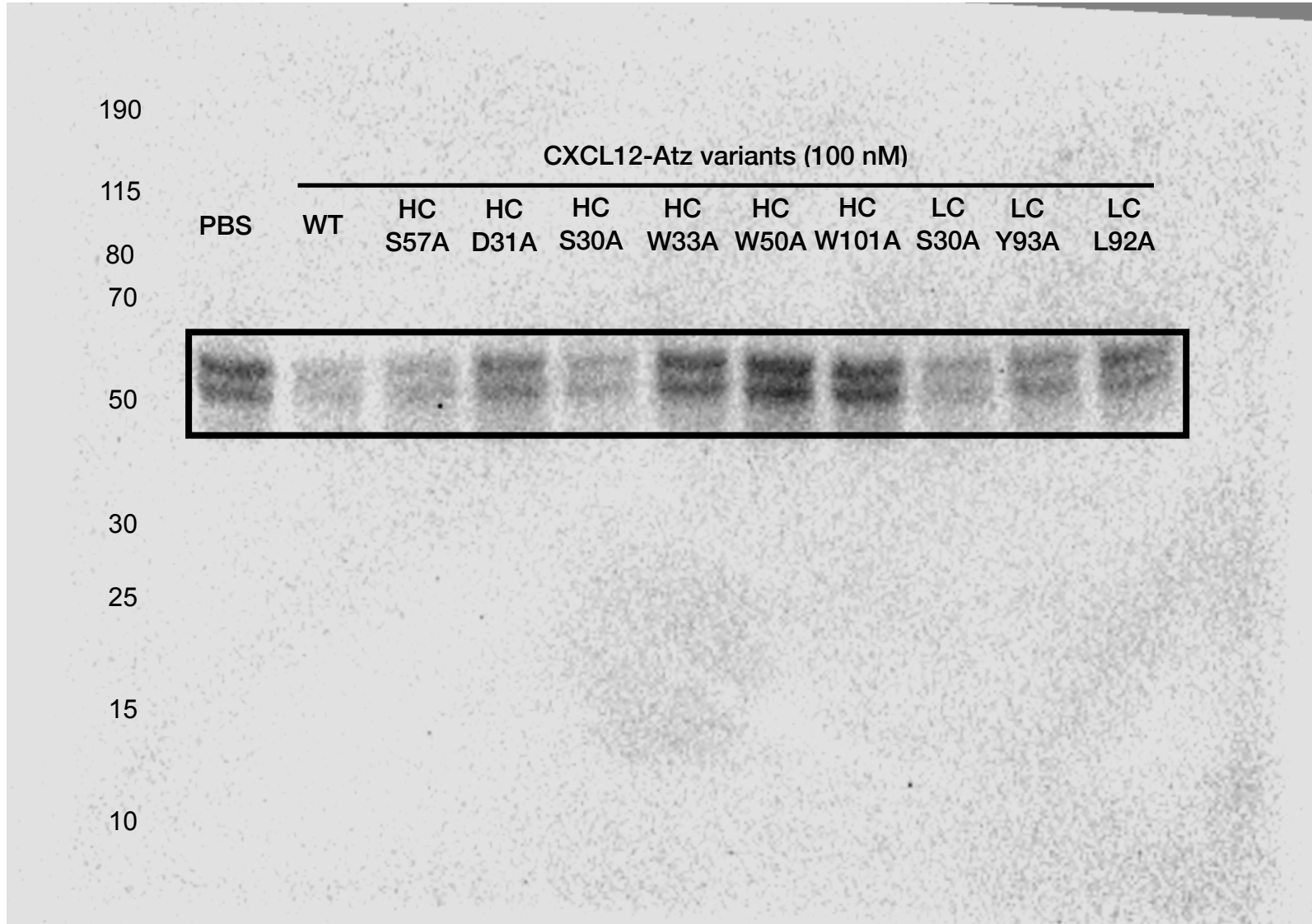

Tubulin blot

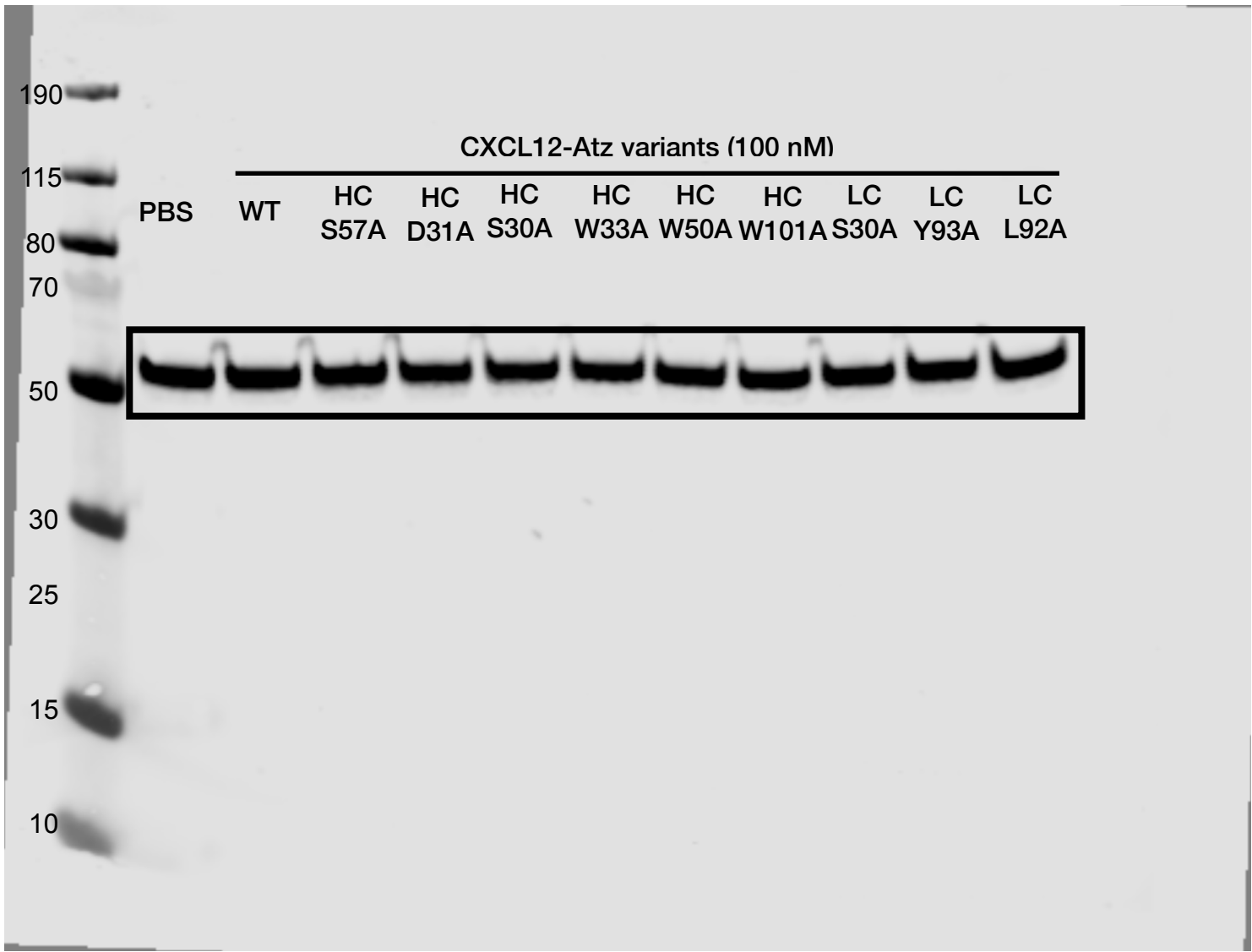

f. PD-L1 blot

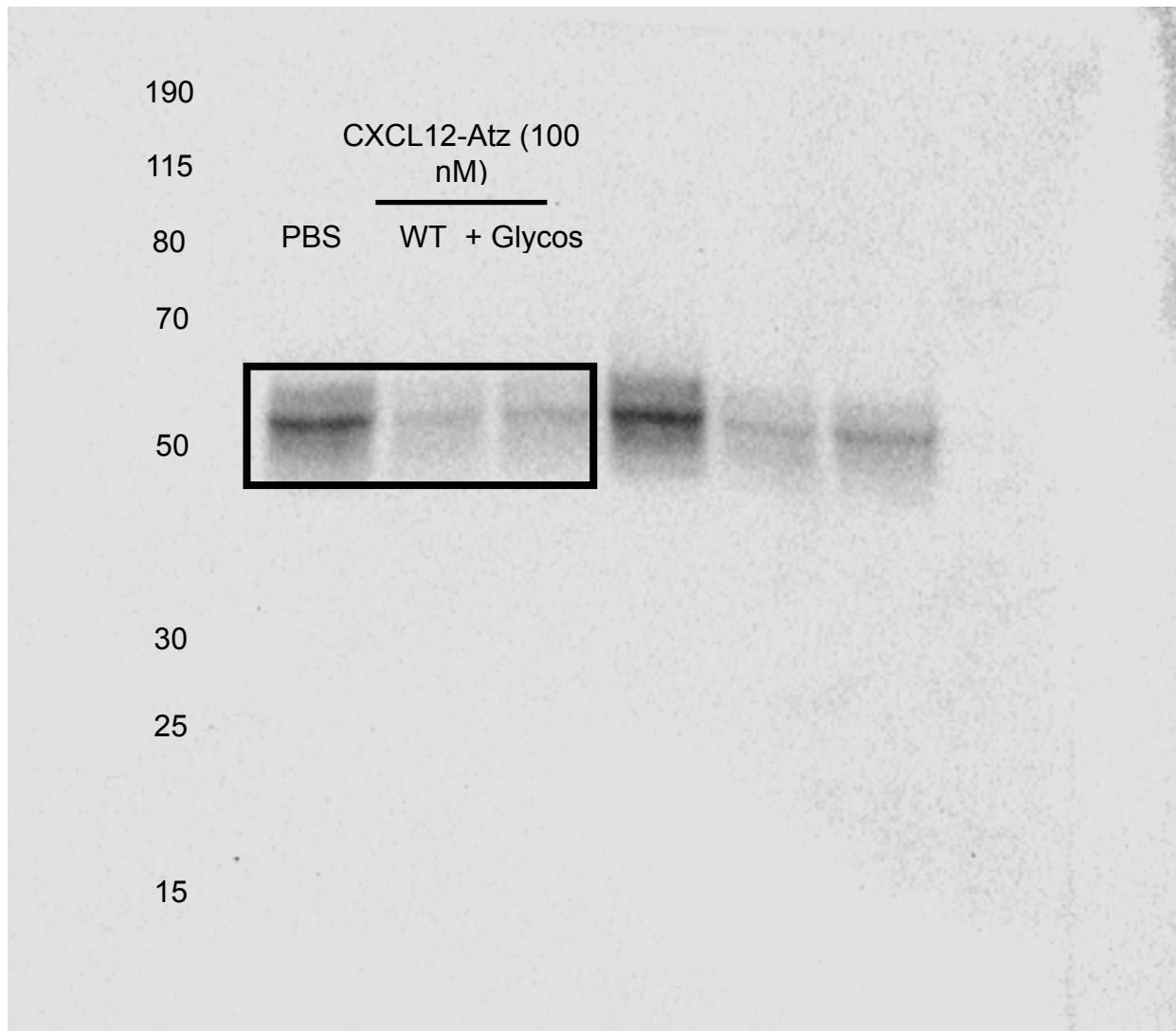

Tubulin blot

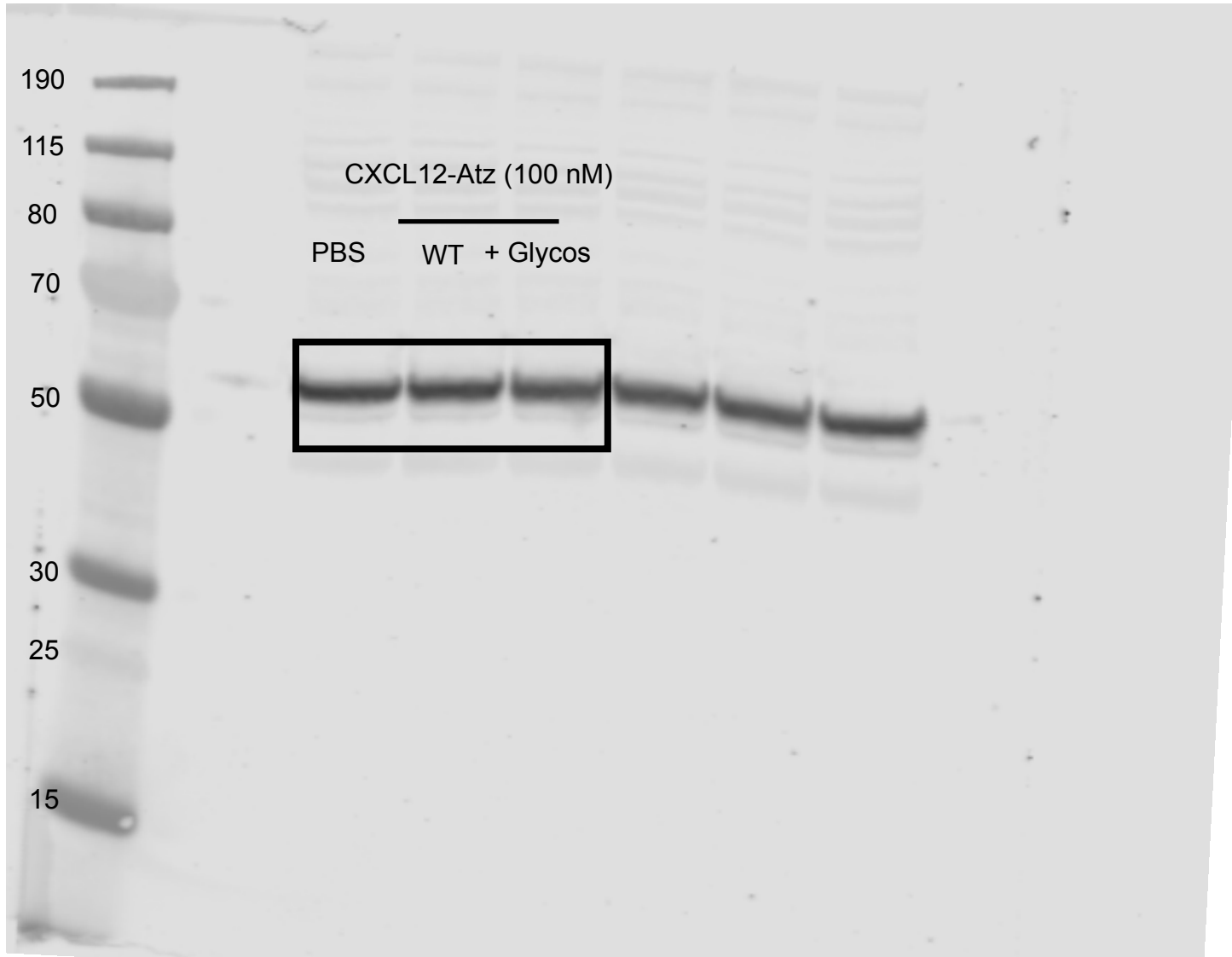

i. PD-L1 blot

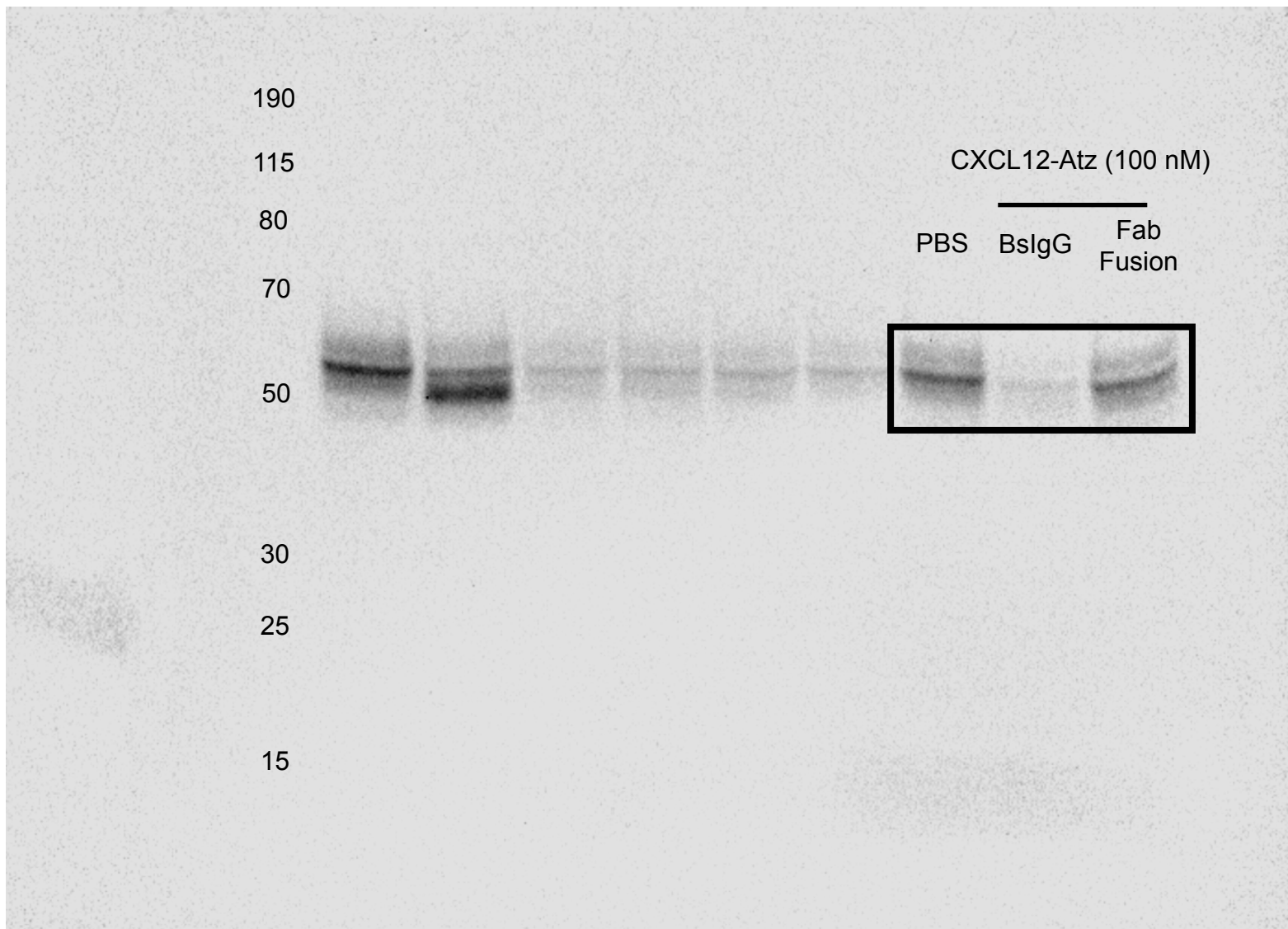

Tubulin blot

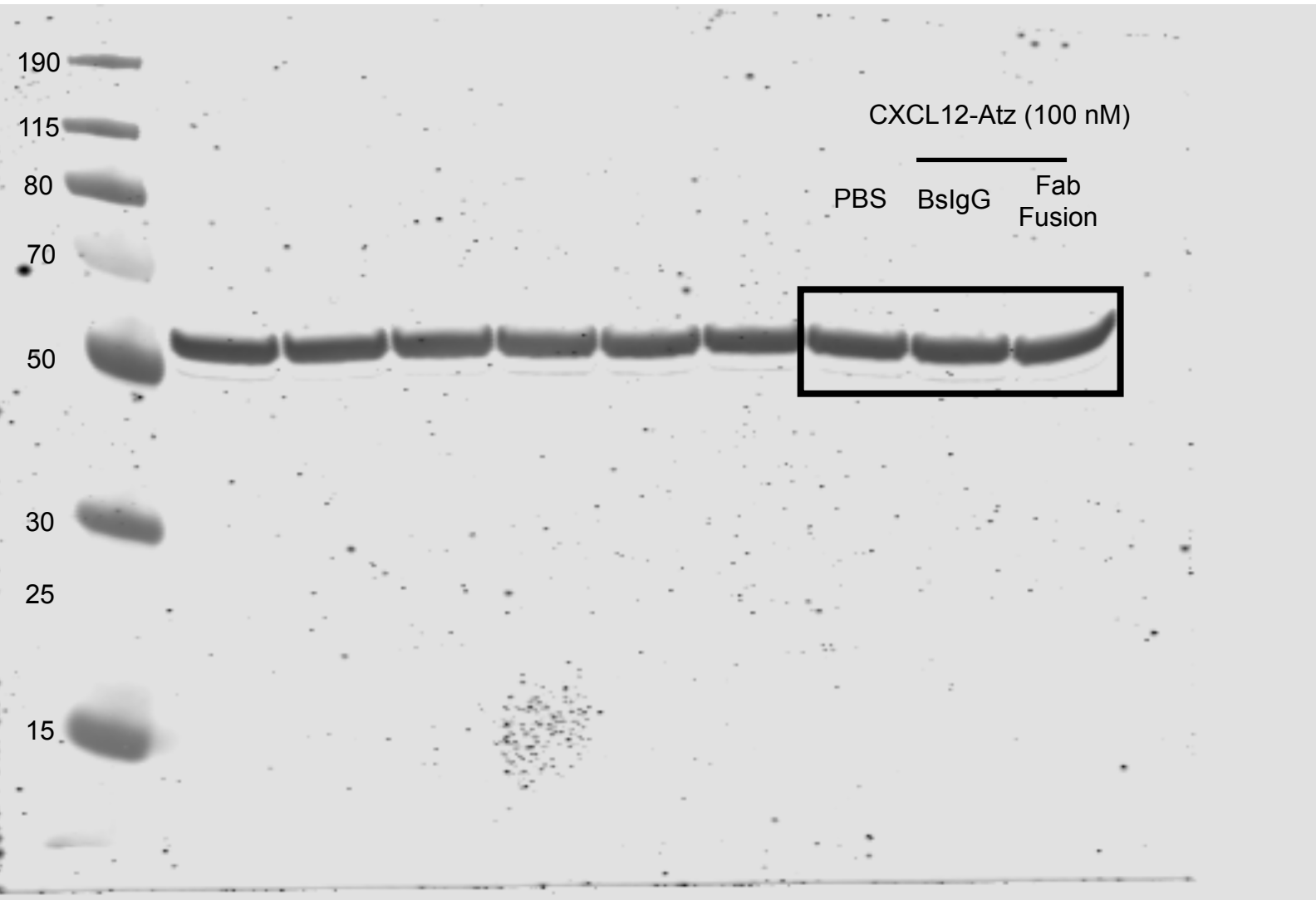

Supplement: Source Data Extended Data Fig. 4 — Full-length, unprocessed gels or blots. [file 41587_2022_1456_MOESM16_ESM.pdf]

Extended Data Figure 5

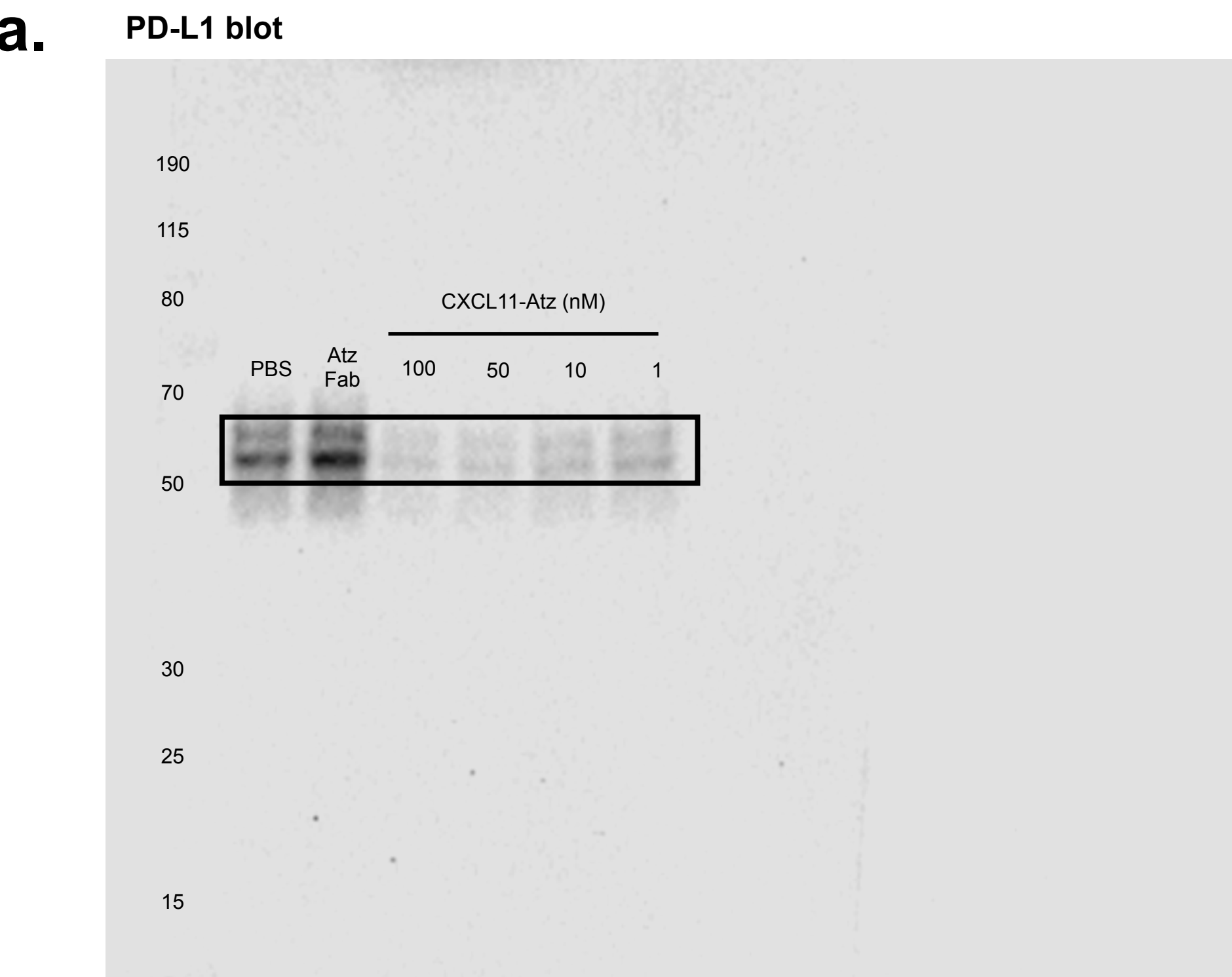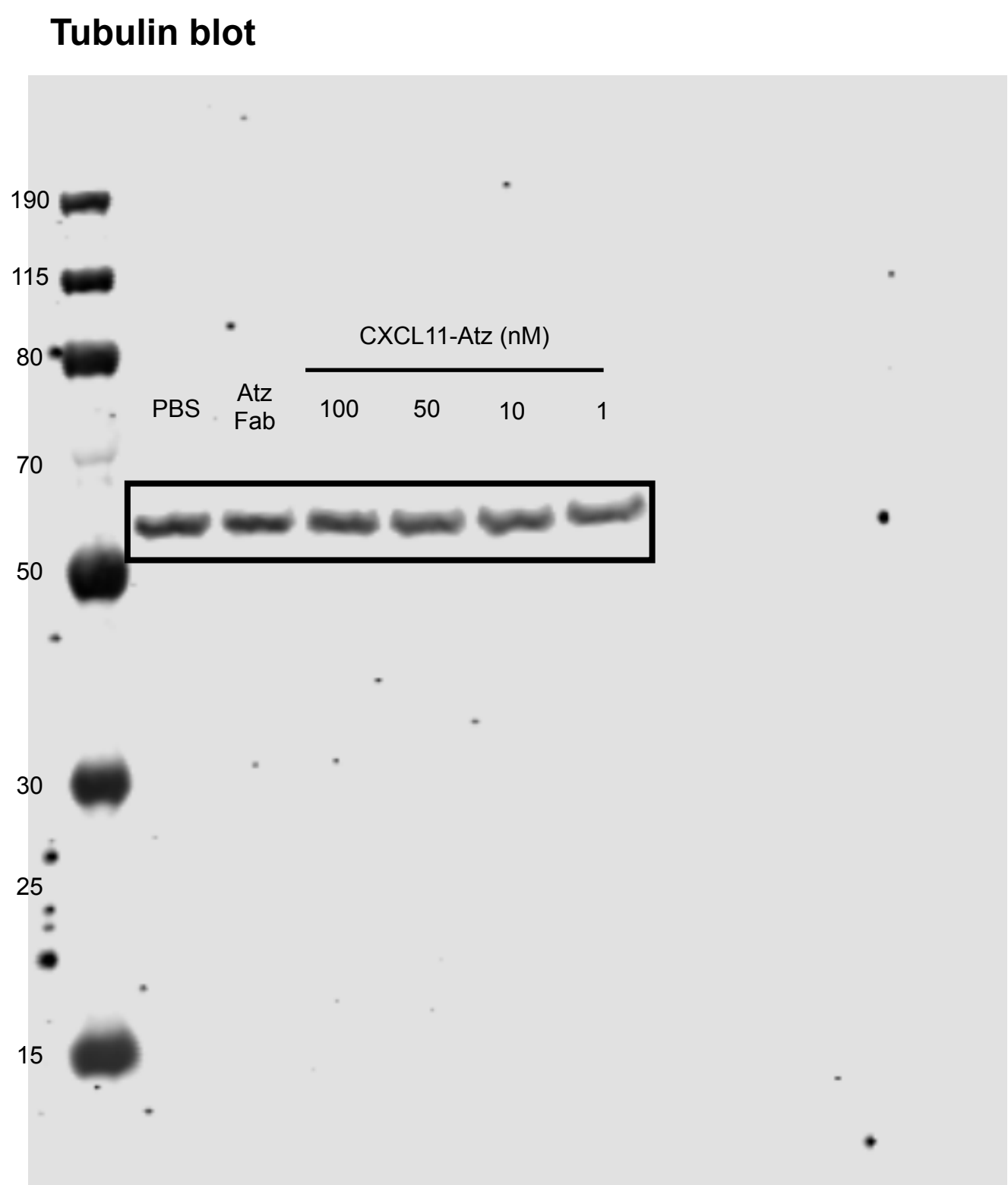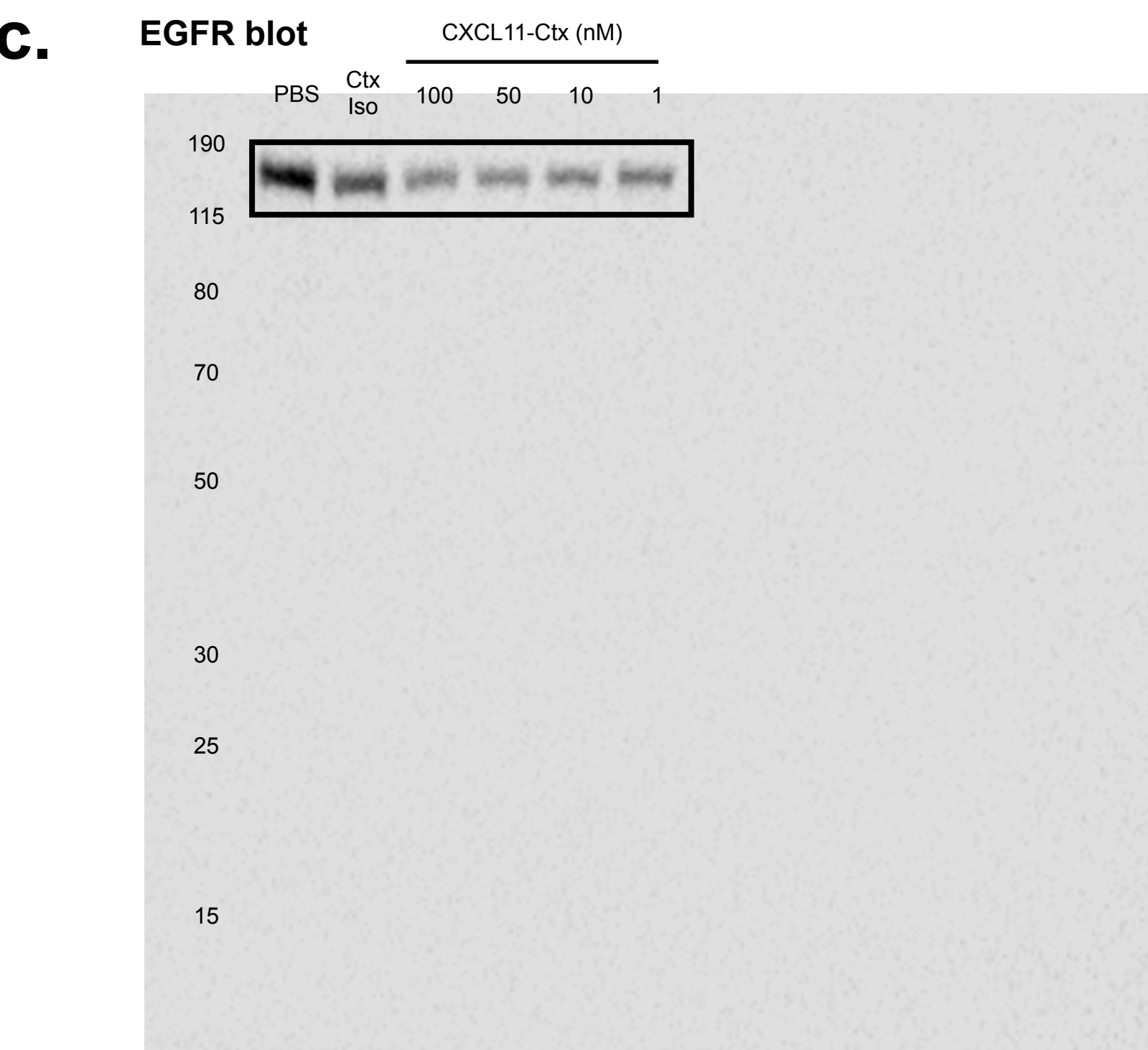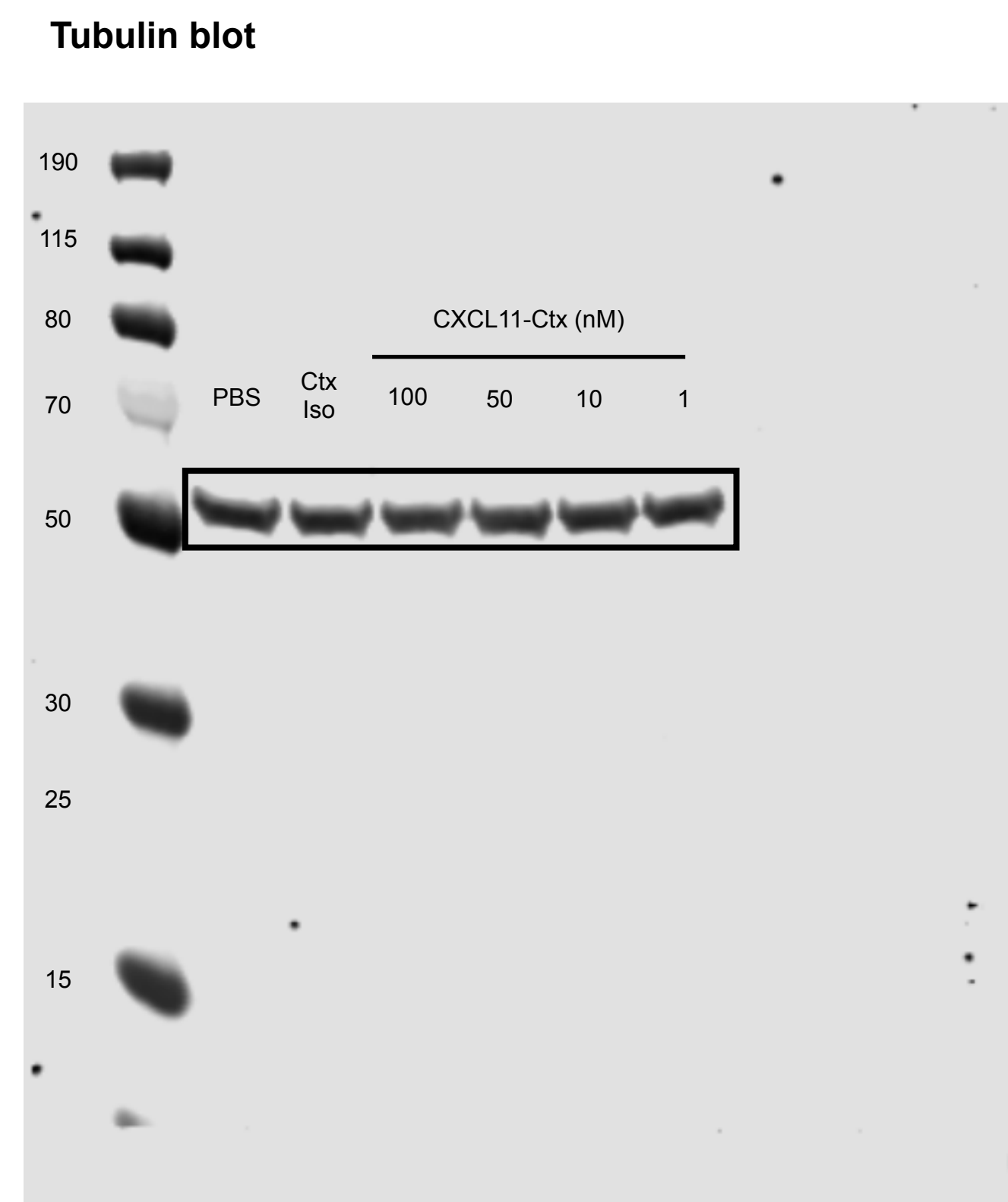

Supplement: Source Data Extended Data Fig. 5 — Full-length, unprocessed gels or blots. [file 41587_2022_1456_MOESM18_ESM.pdf]
