# Supplementary material for: Modular cytokine receptor-targeting chimeras for targeted degradation of cell surface and extracellular proteins
Source: Nat Biotechnol. 2022 Sep 22;41(2):273–81. doi: 10.1038/s41587-022-01456-2 (PMC9931583; doi:10.1038/s41587-022-01456-2)

Extended Data Figure 1

a. PD-L1 blot

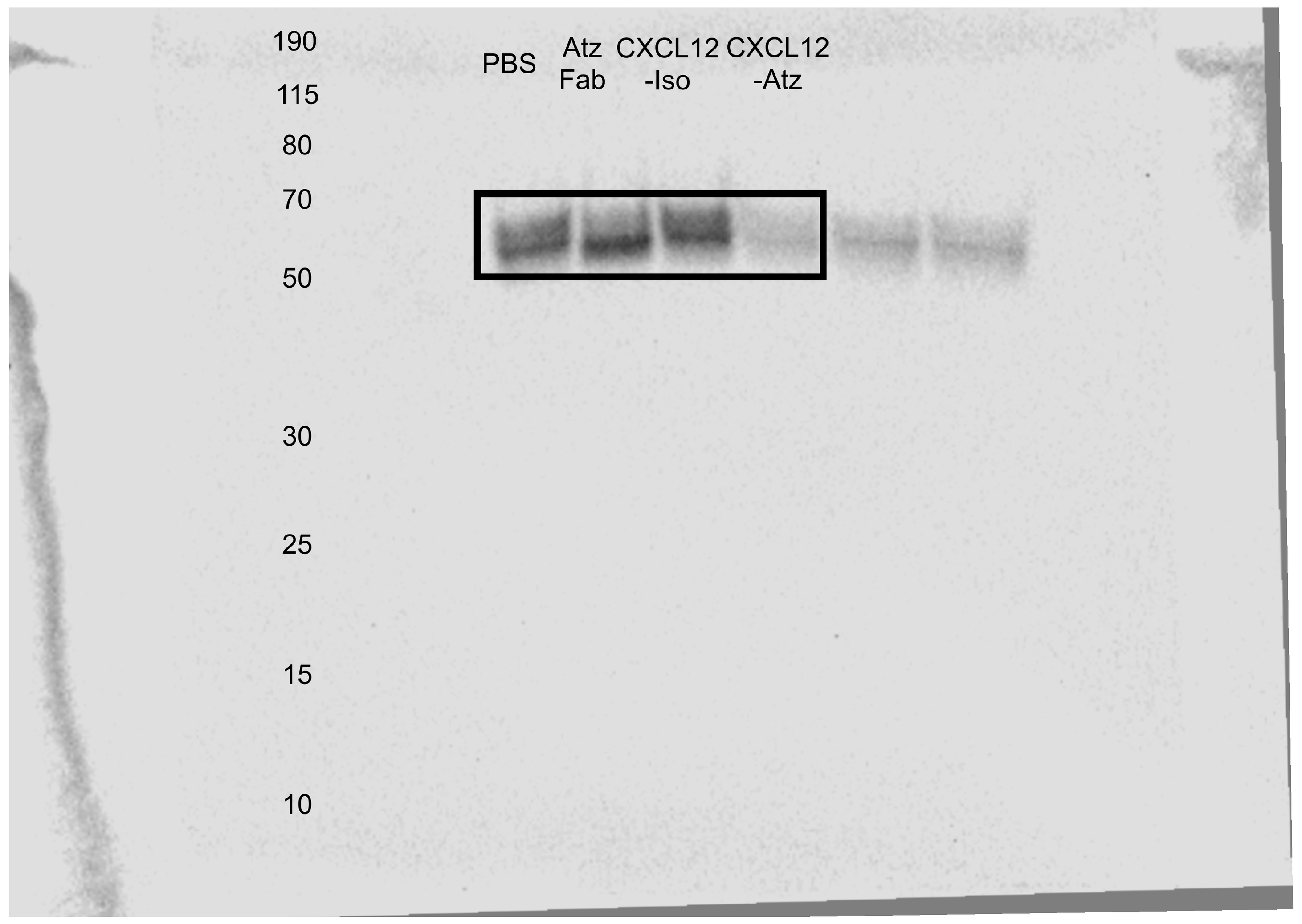

Tubulin blot

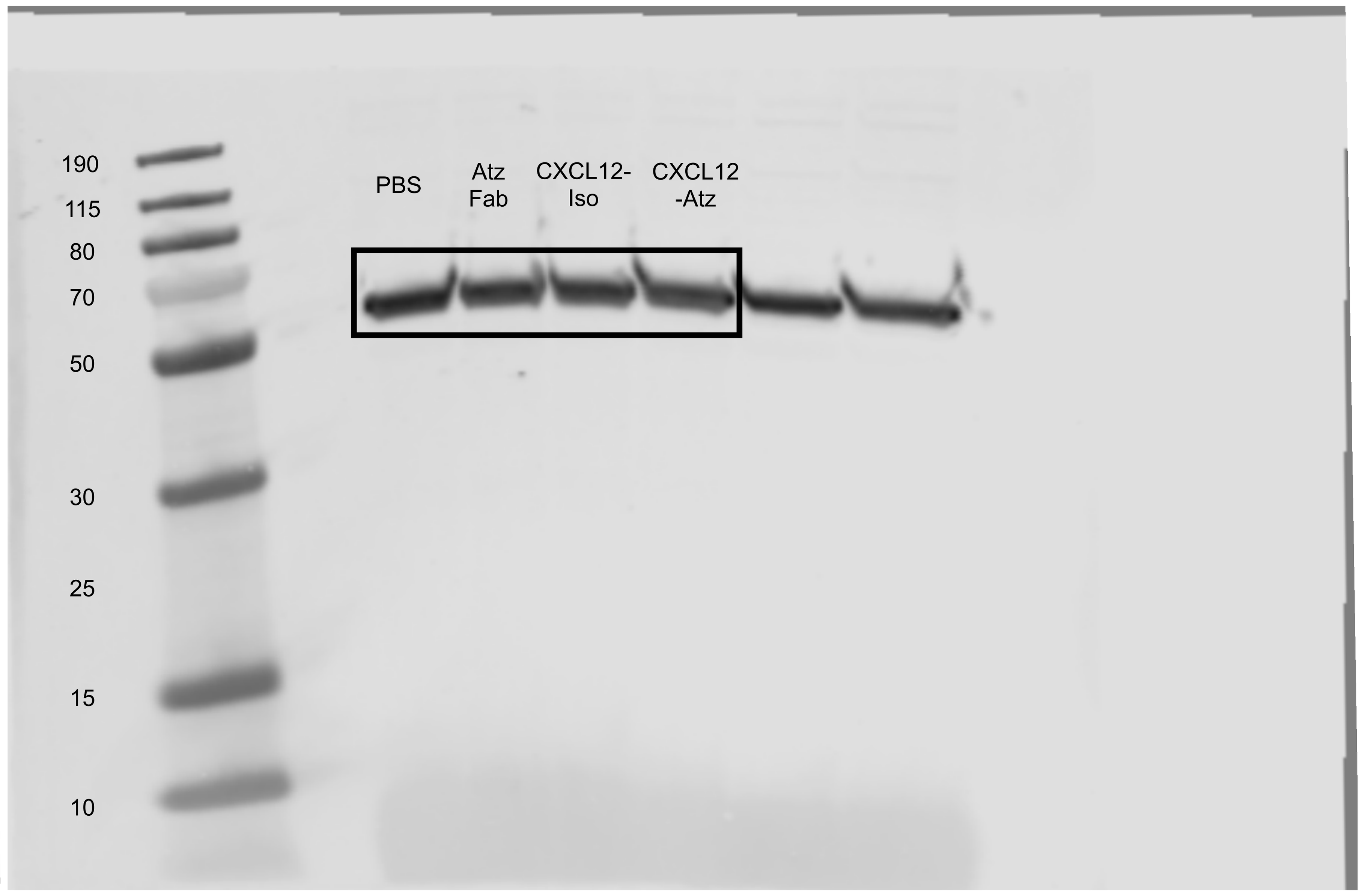

b. PD-L1 blot

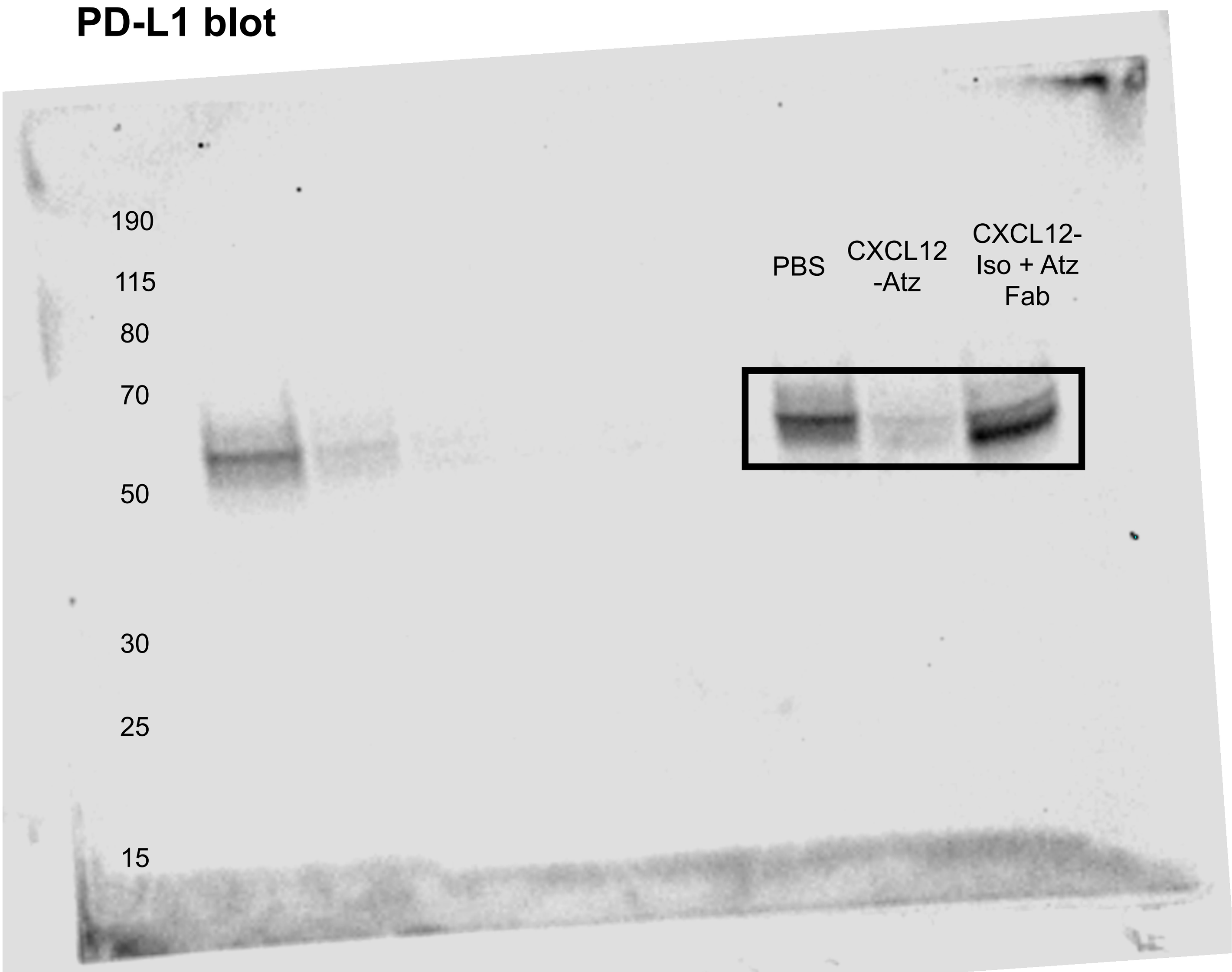

Tubulin blot

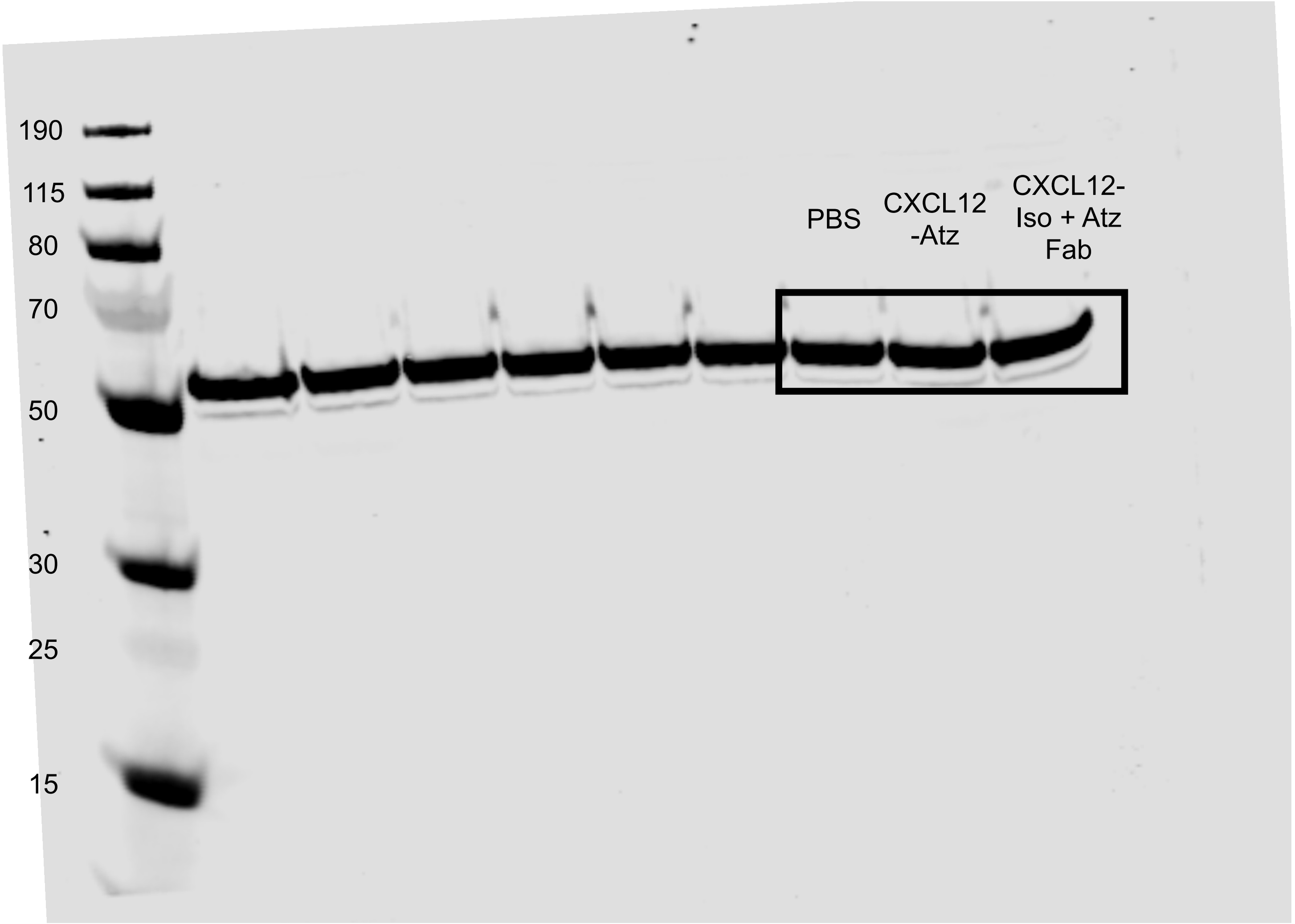

d. PD-L1 blot

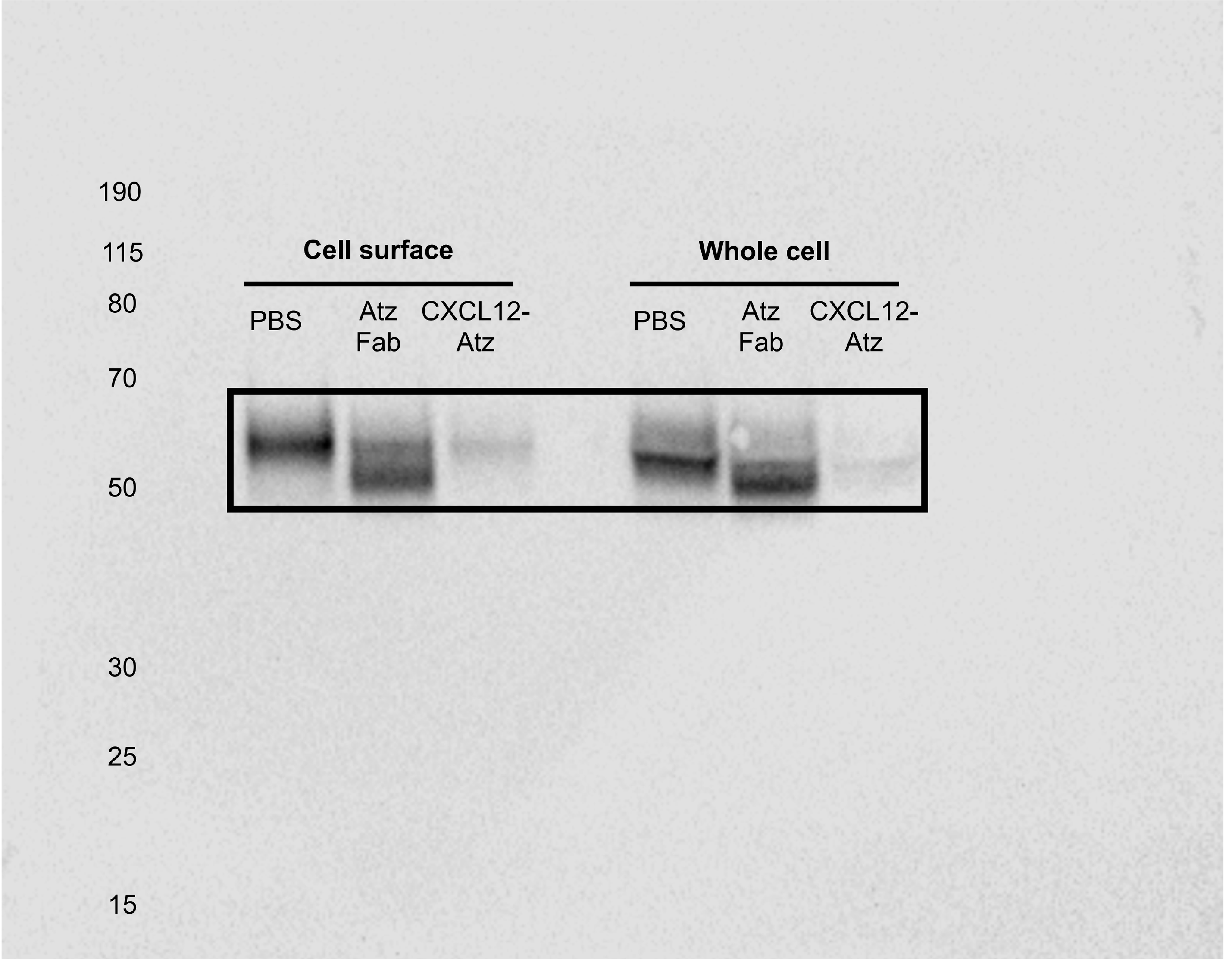

Whole proteome blot

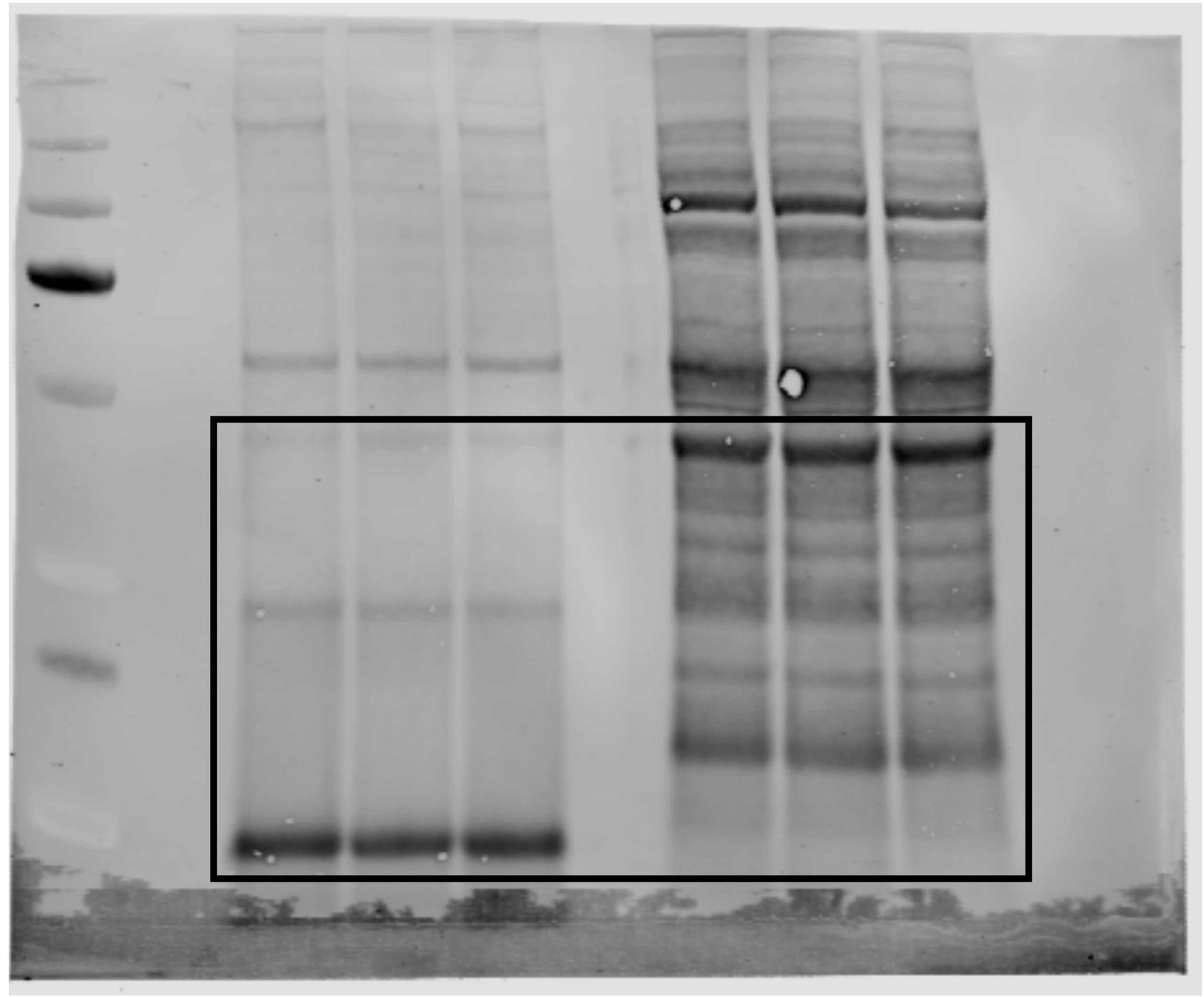

Extended Data Figure 1

e. **PD-L1 blot**

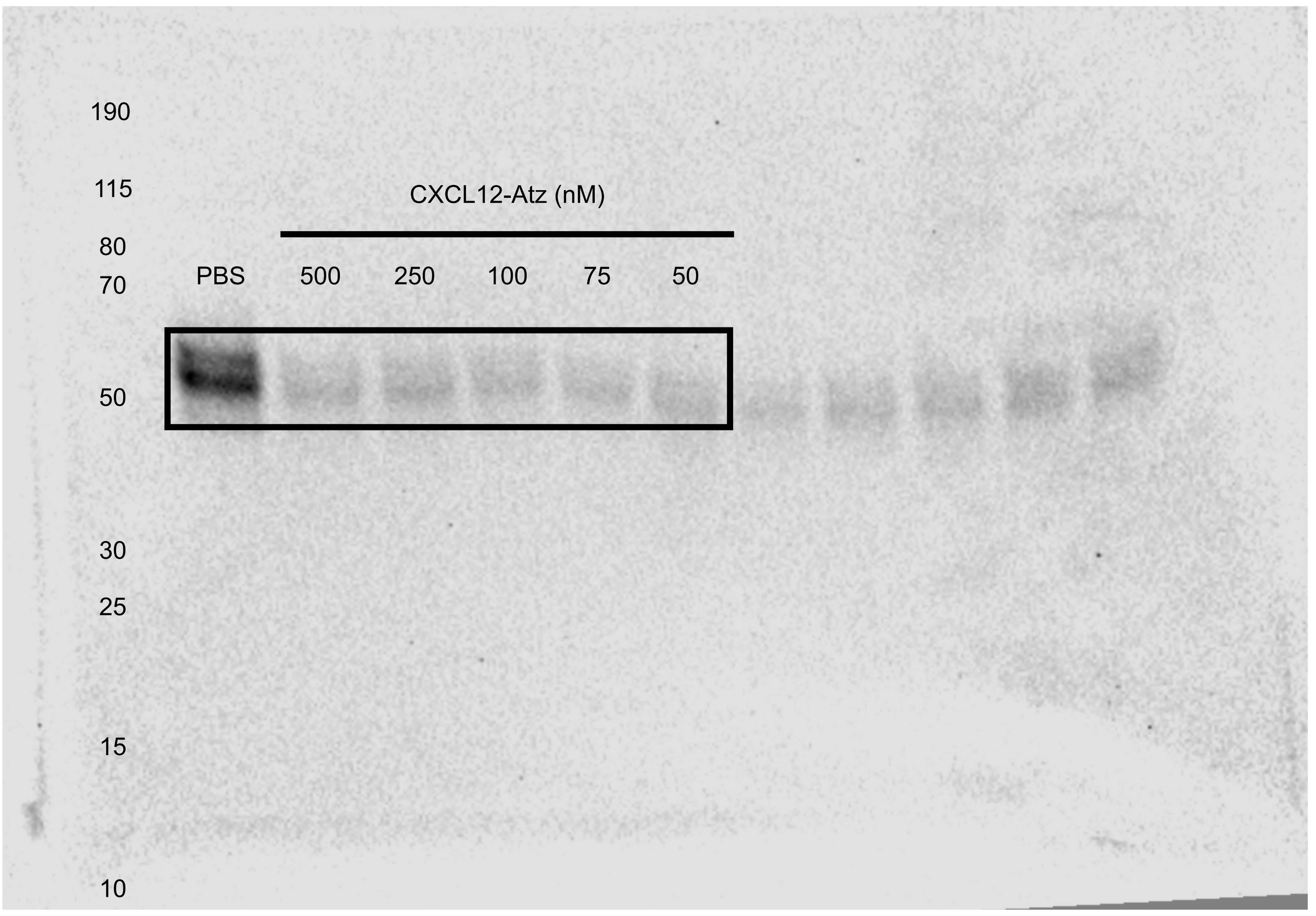

Tubulin blot

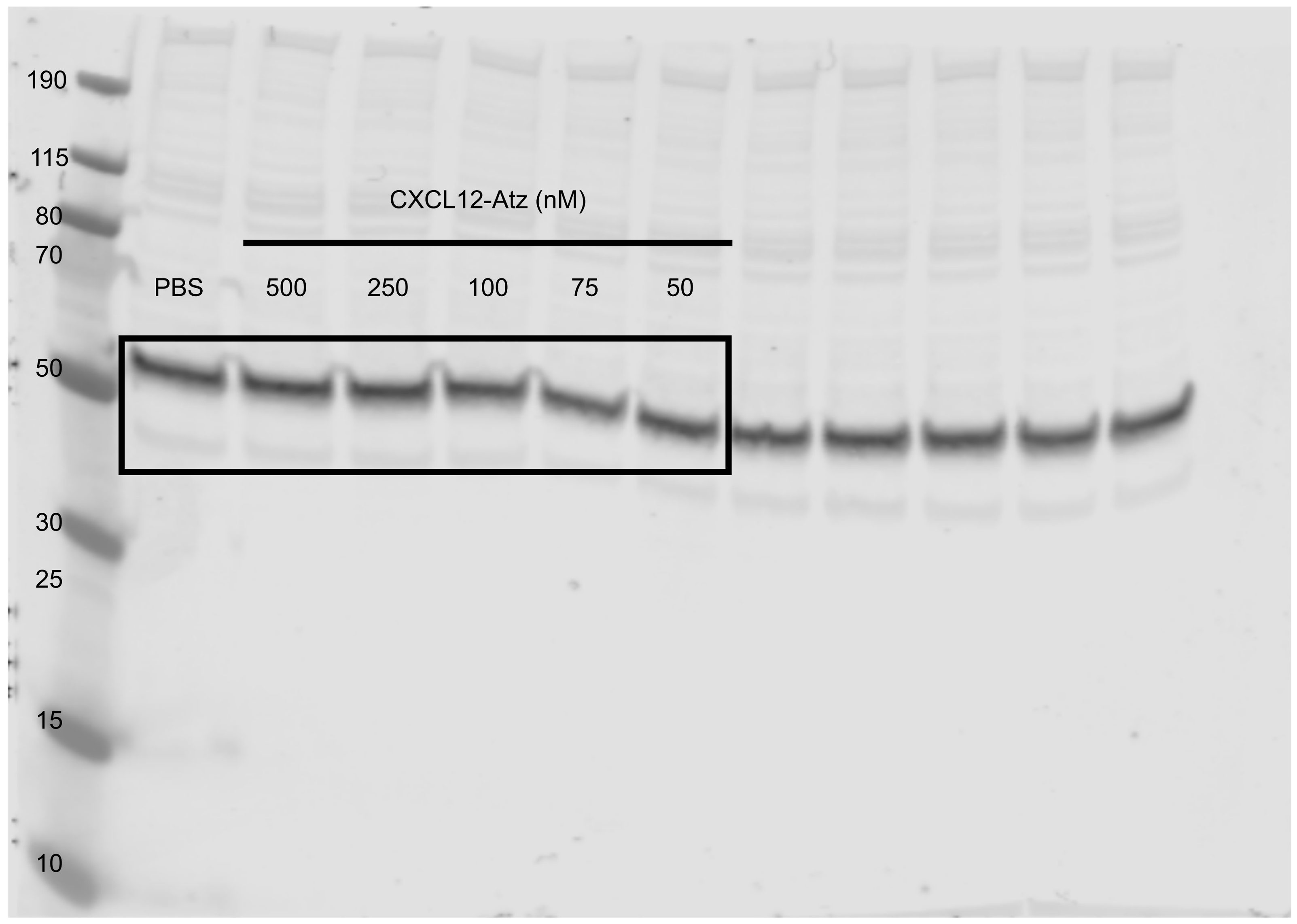

Supplement: Source Data Extended Data Fig. 1 — Full-length, unprocessed gels or blots. [file 41587_2022_1456_MOESM12_ESM.pdf]
